# Prognostic prediction and immune infiltration analysis based on ferroptosis and EMT state in hepatocellular carcinoma

**DOI:** 10.3389/fimmu.2022.1076045

**Published:** 2022-12-15

**Authors:** Zhaochen Liu, Jingju Wang, Suxin Li, Luhao Li, Lin Li, Dingyang Li, Huahu Guo, Dute Gao, Shengyan Liu, Chengshuo Ruan, Xiaowei Dang

**Affiliations:** ^1^ Department of Hepatobiliary and Pancreatic Surgery, The First Affiliated Hospital of Zhengzhou University, Zhengzhou, China; ^2^ Budd-Chiari Syndrome Diagnosis and Treatment Center of Henan Province, Zhengzhou University, Zhengzhou, China

**Keywords:** hepatocellular carcinoma, ferroptosis, emt, immune microenvironment, prognostic model, nomogram

## Abstract

**Background:**

Ferroptosis is one of the main mechanisms of sorafenib against hepatocellular carcinoma (HCC). Epithelial-mesenchymal transition (EMT) plays an important role in the heterogeneity, tumor metastasis, immunosuppressive microenvironment, and drug resistance of HCC. However, there are few studies looking into the relationship between ferroptosis and EMT and how they may affect the prognosis of HCC collectively.

**Methods:**

We downloaded gene expression and clinical data of HCC patients from the Cancer Genome Atlas (TCGA) and International Cancer Genome Consortium (ICGC) databases for prognostic model construction and validation respectively. The Least absolute shrinkage and selection operator (LASSO) Cox regression was used for model construction. The predictive ability of the model was assessed by Kaplan–Meier survival analysis and receiver operating characteristic (ROC) curve. We performed the expression profiles analysis to evaluate the ferroptosis and EMT state. CIBERSORT and single-sample Gene Set Enrichment Analysis (ssGSEA) methods were used for immune infiltration analysis.

**Results:**

A total of thirteen crucial genes were identified for ferroptosis-related and EMT-related prognostic model (FEPM) stratifying patients into two risk groups. The high-FEPM group had shorter overall survivals than the low-FEPM group (p<0.0001 in the TCGA cohort and p<0.05 in the ICGC cohort). The FEPM score was proved to be an independent prognostic risk factor (HR>1, p<0.01). Furthermore, the expression profiles analysis suggested that the high-FEPM group appeared to have a more suppressive ferroptosis status and a more active EMT status than the low- FEPM group. Immune infiltration analysis showed that the myeloid-derived suppressor cells (MDSCs), and regulatory T cells (Tregs) were highly enriched in the high-FEPM group. Finally, a nomogram enrolling FEPM score and TNM stage was constructed showing outstanding predictive capacity for the prognosis of patients in the two cohorts.

**Conclusion:**

In conclusion, we developed a ferroptosis-related and EMT-related prognostic model, which could help predict overall survival for HCC patients. It might provide a new idea for predicting the response to targeted therapies and immunotherapies in HCC patients.

## 1 Introduction

Primary liver cancer is the sixth most common cancer and the third leading cause of cancer death worldwide. As the most common pathological type, hepatocellular carcinoma (HCC) causes a significant cancer burden worldwide (1). Chronic hepatitis B or C virus infection or excessive alcohol consumption are the main causes of HCC (2). With the progress in prevention and systemic treatment of HCC, the incidence and mortality of liver cancer have gradually decreased in recent years (3). Nevertheless, high postoperative recurrence rate and low 5-year survival rate remain clinical problems (4). In-depth understanding of the molecular mechanism of HCC progression and screening out potential tumor-specific markers are of great importance for early diagnosis, treatment and especially prognostic prediction of HCC patients. Ferroptosis is a novel form of regulatory cell death mediated by iron-dependent lipid peroxidation (5), which is closely related to a variety of human diseases. Since it was first defined in 2012, ferroptosis has attracted great attention of medical researchers especially in the field of cancer treatment. Sorafenib is a first-line therapy for advanced HCC. Sorafenib, as a multi-kinase inhibitor, can not only inhibit the proliferation and angiogenesis of tumor cells by inhibiting RAF kinase and several receptor tyrosine kinases (6), but also exert cytotoxic effects by inducing HCC apoptosis (7). In addition, sorafenib is associated with ferroptosis which is considered as a new treatment mechanism (8). Studies have shown that there are multiple resistance mechanisms of ferroptosis in the treatment of HCC with sorafenib, which may lead to the decreased therapeutic effect of sorafenib (9, 10). Therefore, it is necessary to understand the ferroptosis status of different HCC patients to improve the effect of individualized targeted therapies.

EMT is known for participating in various pathophysiological processes like tissue fibrosis and cancer progression (11). With the deepening of research, it has been realized that EMT is a reversible and dynamic process in which epithelial cells acquire mesenchymal phenotype and behavior through dedifferentiation (12). In addition to increasing cell invasiveness and promoting tumor metastasis, EMT is also involved in cancer stem cells (CSC), tumor immune escape and drug resistance, which is considered as a multifunctional tool for tumor progression (13–15). EMT accounts for the complex cellular heterogeneity of HCC to some extent (12), and has been regarded as the main cause of secondary sorafenib resistance in several studies (16, 17). Targeting EMT is expected to be a new therapy to overcome drug resistance in HCC.

As two important biological processes of cancer, the potential relationship between ferroptosis and EMT has attracted more and more attention. Previous study showed that the activation of ZEB1 during EMT promoted the synthesis of polyunsaturated fatty acid (PUFA), and the metabolism of PUFA relied on GPX4. This made cancer cells vulnerable to ferroptosis (18). Subsequently, researchers used human adrenal cortical carcinoma cell line SW13 as a model to explore the link between ferroptosis and EMT (19). They found that treatment with histone deacetylase (HDAC) inhibitors could induce EMT in SW13 cells and increase their sensitivity to ferroptosis by altering intracellular iron levels. In addition, EMT-driven Discoidin Domain Receptor Tyrosine Kinase 2 (DDR2) upregulation stimulated ferroptosis susceptibility in recurrent breast cancer through the Hippo pathway (20). However, the regulatory effect of ferroptosis on EMT has been inconsistent in some studies. A single-cell RNA sequencing study found that the tendency of ferroptosis positively regulated EMT in lung adenocarcinoma (LUAD) epithelial cells (21). In contrast, ferritinophagy-mediated ferroptosis inhibited EMT in gastric cancer cells and HepG2 cells (22, 23). Exploring the crosstalk between ferroptosis and EMT could help develop novel cancer therapies (24).

Increasing evidence suggested there was a complex relationship between ferroptosis and EMT. In this study, we attempt to develop a prognostic model combining ferroptosis-related genes (FRGs) and EMT-related genes (ERGs). A new ferroptosis-related and EMT-related prognostic model (FEPM) was constructed and of great value to predict the prognosis of HCC patients.

## 2 Materials and methods

### 2.1 Data collection and genes acquisition

The level-3 mRNA expression data and corresponding clinicopathological information of HCC patients were downloaded from the Cancer Genome Atlas (TCGA) website^1^ and the International Cancer Genome Consortium (ICGC) website^2^. After excluding patients lacking crucial clinical information, the training cohort (TCGA) comprised 365 patients with HCC. According to the specific sample naming rules of TCGA database, transcriptome sequencing results of 365 tumor samples and 50 normal tissue samples were extracted from gene expression data files. The validation cohort (ICGC) included 231 patients with HCC from Japan. Accordingly, we obtained the gene expression data from 231 tumor samples and 199 normal tissue samples. **Table 1** showed the clinicopathological characteristics of the two cohorts. Log_2_ (TPM+1) was used to normalize gene expression data. 259 FRGs were derived from FerrDb database^3^. 1011 ERGs which undertake the function of encoding proteins were selected from dbEMT 2.0 database^4^.

**Table 1:** Baseline characteristics of HCC patients in the TCGA and ICGC cohorts.

	TCGA cohort	ICGC cohort
No. of patients	365	231
Age(%)		
≤60	173(47.4)	49(21.2)
>60	192(52.6)	182(78.8)
Gender(%)		
Male	246(67.4)	170(73.6)
Female	119(32.6)	61(26.4)
Tumor grade(%)		
G1-G2	230(63.0)	–
G3-G4	130(35.6)	–
NA	5(1.4)	–
AFP(%)		
≤400	213(58.3)	–
>400	63(17.3)	–
NA	89(24.4)	–
Vascular invasion(%)		
No	205(56.2)	–
Yes	106(29.0)	–
NA	54(14.8)	–
TNM stage(%)		
I-II	254(69.6)	141(61.0)
III-IV	87(23.9)	90(39.0)
NA	24(6.5)	–
Child-Pugh(%)		
A	216(59.2)	–
B-C	22(6.0)	–
NA	127(34.8)	–
Fibrosis(%)		
No	74(20.3)	–
Yes	135(37.0)	–
NA	156(42.7)	–

NA, not available.

### 2.2 Screening crucial genes for the model

To begin with, the gene expression data of TCGA dataset was analyzed by the R package “DESeq2” (25). The gene count matrix was prepared as the input file for differentially expression analysis. The comparison was about tumor samples versus normal tissue samples. The thresholds for screening differentially expressed genes (DEGs) were set at |Log2FC| > 1 and adjusted p< 0.05. As the Venn diagram showed, the overlapping genes were identified as differentially expressed FRGs and ERGs. Thereafter, prognostic genes were recognized by univariate Cox regression analysis. The normalized expression values of differentially expressed FRGs and ERGs from tumor samples were analyzed as covariates in the univariate Cox regression. To avoid multicollinearity, FRGs and ERGs with prognostic significance (p< 0.0005) passed through the LASSO regression for variable selection and shrinkage. This analysis process generated the crucial genes participating in model construction and their corresponding coefficients by means of the “glmnet” package (26). Finally, the FEPM score was obtained according to the following formula where *Coef_i_* is the coefficient and *Exp_i_* is the expression value of each crucial gene.


$$FEPM\;score=\sum_{i=1}^{\text{n}}Coef_{i}\;\times \;Exp_{i}$$

### 2.3 Functional analysis

To explore the enriched pathways of different subgroups, Gene Set Enrichment Analysis was performed in GSEA software (version 4.2.2). Significantly enriched pathways were satisfied with the following standards simultaneously: nominal p value< 0.05, false discovery rate (FDR)< 0.25, and normalized enrichment score > 1. The gene set “c2.cp.kegg.v7.5.1.symbols. gmt” was selected as a reference gene set.

The prognostic genes with statistically significance (p< 0.0005) were uploaded into the STRING database^5^, for the purpose of a protein–protein interaction (PPI) network. Cytoscape (version 3.9.0) was utilized to visualize the network. The clustering effect of the prognostic model was evaluated by principal component analysis (PCA).

### 2.4 Analysis of immune cell infiltration

The enrichment scores of immune cells were calculated according to the single-sample GSEA (ssGSEA) approach. This analysis was implemented *via* the R package “GSVA”. The phenotype feature list of immune cells was retrieved from the previous pan-cancer analysis publication (27). The immune infiltration data that were generated by the CIBERSORT algorithm can be found on the GDC website^6^. A validated leukocyte gene signature matrix (LM22) was used for the deconvolution in the CIBERSORT analysis (28).

### 2.5 Statistical analysis

R software (version 4.1.2) and IBM SPSS Statistics (version 21.0) were utilized for statistical analysis. The “survminer” package was used to perform univariate and multivariate Cox regressions. The hazard function h(t) meaning the risk of dying at survival time “t” was used as the response variable of Cox model. FEPM score and the clinical features including gender (female vs. male), age (> 60 vs.<= 60), histological grade (G3-G4 vs. G1-G2), Child-Pugh classification (B-C vs. A), liver fibrosis (Yes vs. No), α-fetoprotein (AFP > 400 vs.<= 400 ug/L), vascular invasion (Yes vs. No), and TNM stage (III-IV vs. I-II) were used as the covariates. The survival analysis for different subgroups was displayed in the Kaplan–Meier curves. The differences between the curves were compared utilizing the log-rank test. The count data were compared utilizing the Chi-square test. The significance of differences in specific gene expressions or immune cell infiltration was assessed by Wilcoxon test. The “rms” package was used for construction and verification of nomogram. The “ggDCA” package was used for decision curve analysis. The “ggplot2”, “ggpubr”, “ggbiplot”, “pheatmap” packages were applied to visualization.

## 3 Results

### 3.1 A 13-gene signature showing high accuracy of survival prediction in the TCGA cohort

**Figure 1:**
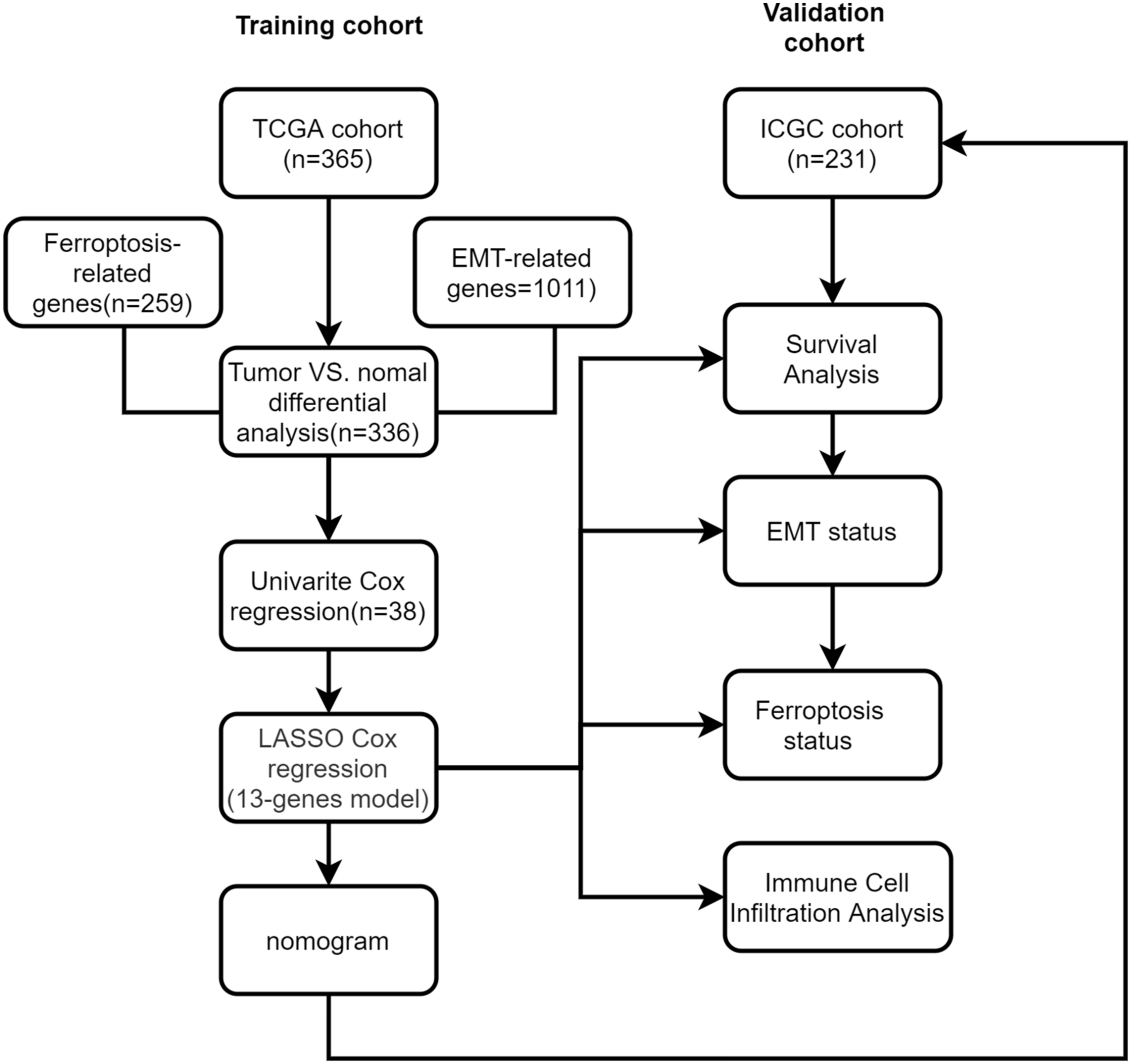
Study flow chart.

**Figure 2:**
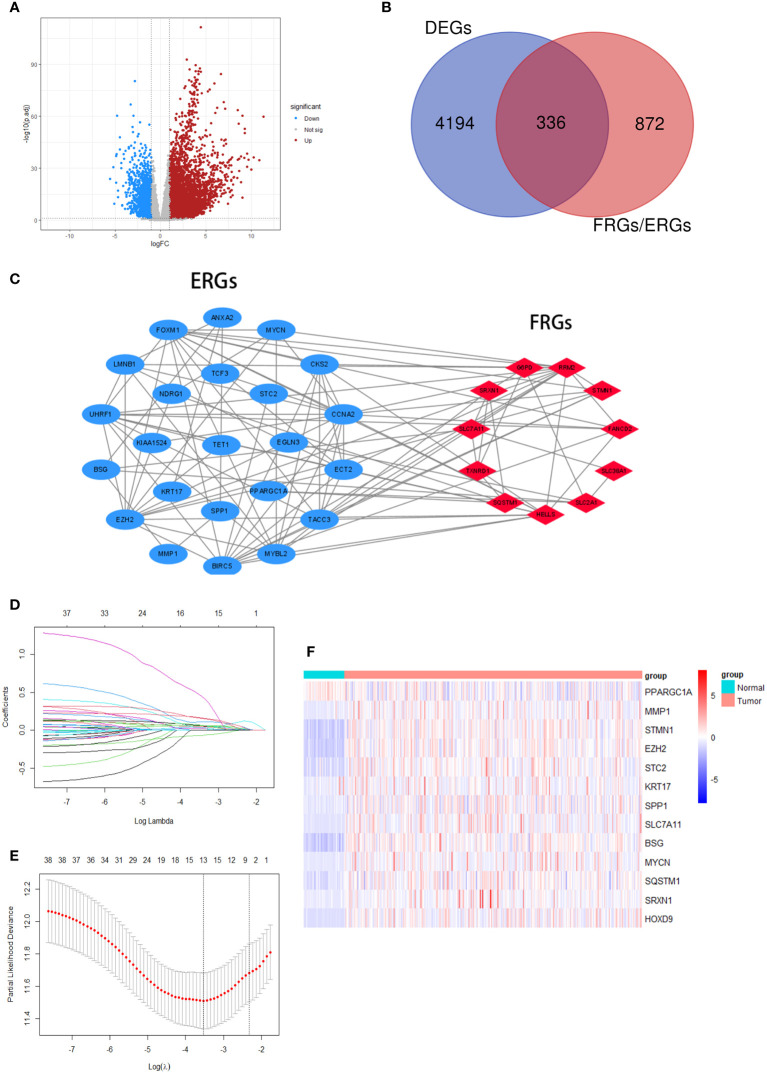
Screening crucial genes for the model. **(A)** A volcanic plot of DEGs. **(B)** A Venn diagram indicating that 336 differentially expressed FRGs and ERGs were identified in the TCGA cohort. **(C)** A PPI network suggesting the relationship between prognostic FRGs and ERGs. **(D, E)** LASSO regression was performed to generate the crucial genes. **(F)** A heatmap showing the expression of the 13 crucial genes in the tumors and normal tissues of the TCGA cohort.

**Figure 3:**
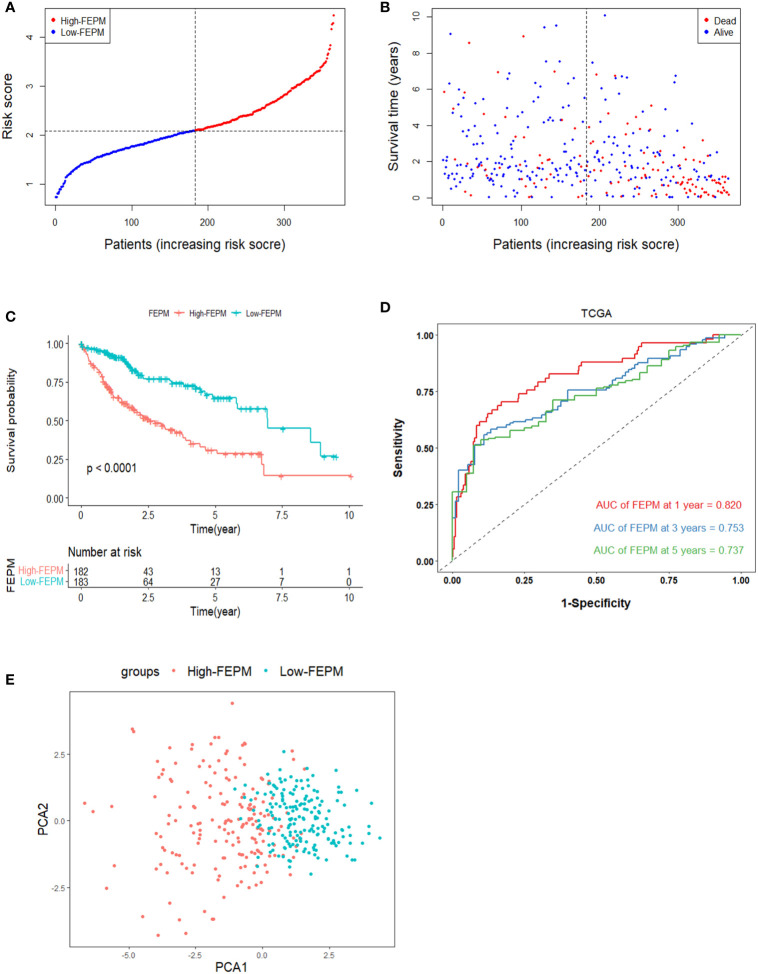
Survival analysis of FEPM in the TCGA cohort. **(A, B)** Distributions of FEPM scores and survival status of HCC patients in the TCGA cohort. **(C)**Kaplan–Meier curves showed that patients in the high-FEPM group had worse prognosis than those in the low-FEPM group in the TCGA cohort. **(D)** ROC curves of FEPM for predicting the 1/3/5-years survival in the TCGA cohort. **(E)** PCA plots of the TCGA cohort based on FEPM.

We made a flow chart to refine the main idea of this study (**Figure 1**). A total of 1208 ERGs and FRGs were included in this study, among which 336 genes were differentially expressed genes (DEGs) between tumors and normal tissues (**Figures 2A, B**). Subsequent univariate Cox regression analysis identified 38 candidate prognostic genes. PPI network of these genes showed an intricate interaction between FRGs and ERGs (**Figure 2C**). The LASSO Cox regression analysis identified 13 crucial genes for ferroptosis-related and EMT-related prognostic model (FEPM) based on the optimal value of λ (**Figures 2D, E**). The expressions of the 13 genes were as shown in the heat map (**Figure 2F**). Among them, there were 9 ERGs (PPARGC1A, MMP1, EZH2, STC2, KRT17, SPP1, BSG, MYCN and HOXD9), 3 FRGs (SLC7A11, STMN1 and SRXN1) and one gene (SQSTM1) belonging to ERGs as well as FRGs. The risk score for each patient was calculated based on the formula mentioned above. The TCGA cohort was classified as a high-FEPM group (182 patients) and a low-FEPM group (183 patients), regarding the median risk score as the cutoff value (**Figure 3A**). As illustrated in Kaplan-Meier curves, patients in the high-FEPM group had significantly shorter overall survivals (p< 0.0001) than those in the low-FEPM group (**Figure 3C**). Patients with higher risk scores died earlier and survived for a shorter time (**Figure 3B**). The area under the curve (AUC) reached 0.820 at 1 year, 0.753 at 3 years, and 0.737 at 5 years according to time-dependent ROC curves (**Figure 3D**). PCA showed that patients could be distinctly separated into two subgroups (**Figure 3E**).

### 3.2 Validation of the prognostic model in the ICGC cohort

**Figure 4:**
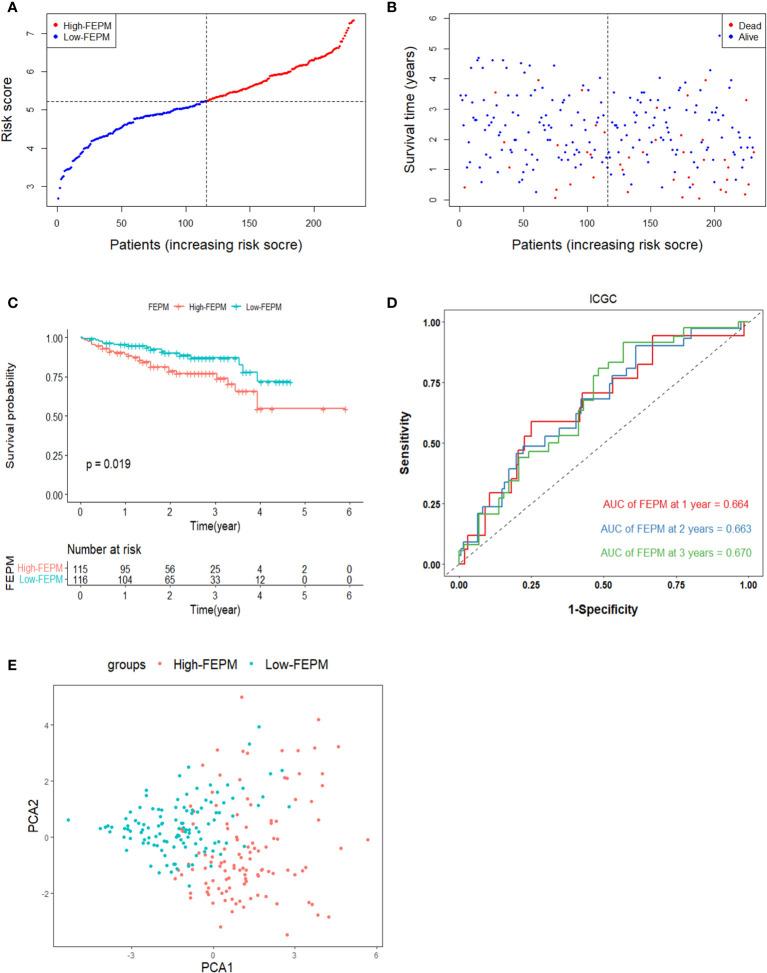
Survival analysis of FEPM in the ICGC cohort. **(A, B)** Distributions of FEPM scores and survival status of HCC patients in the ICGC cohort. **(C)**Kaplan–Meier curves showed that patients in the high-FEPM group had worse prognosis than those in the low-FEPM group in the ICGC cohort. **(D)** ROC curves of FEPM for predicting the 1/2/3-years survival in the ICGC cohort. **(E)** PCA plots of the ICGC cohort based on FEPM.

To avoid overfitting, the ICGC cohort were enrolled in the study to validate the model. Risk scores were figured out according to the same calculation in TCGA cohort. The ICGC cohort was classified as a high-FEPM group (115 patients) and a low-FEPM group (116 patients) (**Figure 4A**). Consistent with the results of the TCGA cohort, the prognosis of patients in the high-FEPM group was worse than that in the low-FEPM group (p< 0.05) (**Figure 4C**). More death events occurred in the high-FEPM group (**Figure 4B**). Because only two patients survived over 5 years, the 5-years AUC was not calculated in the ICGC cohort. The AUC reached 0.664 at 1 year, 0.663 at 2 years, and 0.670 at 3 years according to ROC curves (**Figure 4D**). PCA revealed distinct clustering of patients in the two subgroups. (**Figure 4E**).

### 3.3 FEPM score is an independent prognostic factor

**Figure 5:**
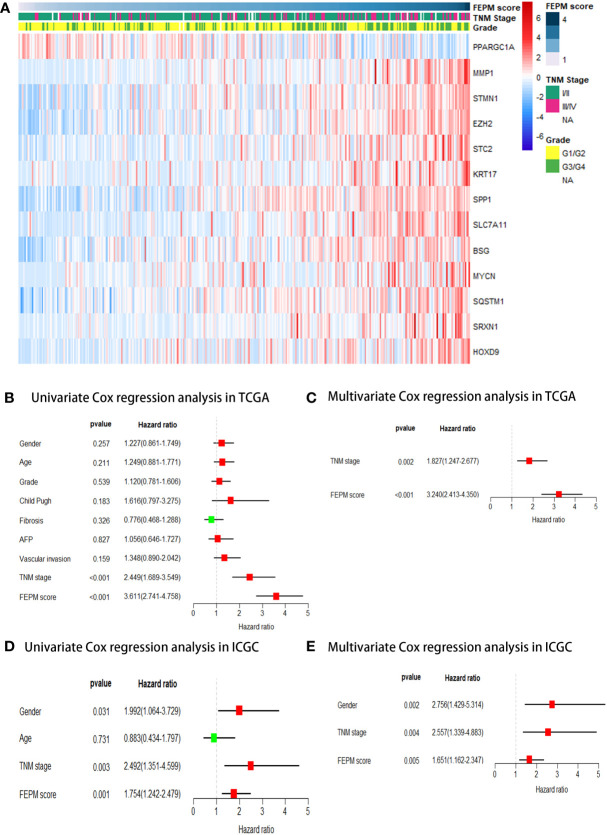
**(A)** A heatmap showed the relationship between FEPM score and clinicopathological features in the TCGA cohort. **(B-E)** Univariate and multivariate Cox regression analyses proved that FEPM score was an independent prognostic factor in the TCGA cohort and ICGC cohort.

As shown in **Table 2**, PPARGC1A was a protective factor for HCC patients (HR< 1, p< 0.001), and the rest genes were risk factors (HR > 1, p< 0.001). The heat map showed different expression levels of these genes in the two subgroups of TCGA cohort (**Figure 5A**). With the ascending of FEPM score, the expression level of PPARGC1A decreased but the expression levels of other genes increased. Moreover, it is interesting to find an evident correlation between FEPM score and clinicopathological features including TNM stage and tumor grade.

**Table 2:** The 13 genes in the LASSO model.

Gene	HR	HR.95L	HR.95H	P value
PPARGC1A	0.803969	0.715518	0.903355	0.000243
MMP1	1.382984	1.227222	1.558516	1.05E-07
EZH2	1.565198	1.314143	1.864215	5.10E-07
STC2	1.355069	1.19444	1.5373	2.36E-06
KRT17	1.257204	1.126313	1.403307	4.49E-05
SPP1	1.131773	1.07487	1.191687	2.56E-06
BSG	1.424203	1.188262	1.706992	0.00013
MYCN	1.266411	1.11247	1.441653	0.000355
HOXD9	1.320256	1.159237	1.50364	2.83E-05
SLC7A11	1.326782	1.175333	1.497746	4.82E-06
STMN1	1.398081	1.213305	1.610998	3.60E-06
SRXN1	4.416865	2.005762	9.726327	0.000226
SQSTM1	1.308961	1.129643	1.516743	0.000341

To further demonstrate the relationship between FEPM and clinicopathological features, we contrasted the baseline data between the two subgroups using the chi-square test. Patients in the high-FEPM group exhibited more advanced TNM stages (III-IV, p< 0.001), higher histological grades (G3-G4, p< 0.001), higher alpha-fetoprotein levels (> 400, p = 0.003) and higher vascular invasion possibilities (p< 0.001) in the TCGA cohort. A later TNM stage was also observed in the high-FEPM group in the ICGC cohort (III-IV, p = 0.006) (**Table 3**).

**Table 3:** Relationships between FEPM and clinicopathological features of HCC patients.

Characteristics	TCGA			ICGC		
	Low-FEPM	High-FEPM	P value	Low-FEPM	High-FEPM	P value
Total case	183	182		116	115	
Age(%)			0.270			0.654
≤60	92(50.3)	81(44.5)		26(22.4)	23(20.0)	
>60	91(49.7)	101(55.5)		90(77.6)	92(80.0)	
Gender(%)			0.710			0.315
Male	125(68.3)	121(66.5)		82(70.7)	88(76.5)	
Female	58(31.7)	61(33.5)		34(29.3)	27(23.5)	
Tumor grade(%)			<0.001			–
G1-G2	133(73.9)	97(53.9)		–	–	
G3-G4	47(26.1)	83(46.1)		–	–	
AFP			0.003			
≤400	123(84.2)	90(69.2)		–	–	
>400	23(15.8)	40(30.8)		–	–	
Vascular invasion(%)			0.001			–
No	123(74.5)	82(56.2)		–	–	
Yes	42(25.5)	64(43.8)		–	–	
TNM stage(%)			0.001			0.006
I-II	144(82.3)	110(66.3)		81(69.8)	60(52.2)	
III-IV	31(17.7)	56(33.7)		35(30.2)	55(47.8)	
Child-Pugh(%)			0.764			–
A	125(91.2)	91(90.1)		–	–	
B-C	12(8.8)	10(9.9)		–	–	
Fibrosis(%)			0.317			–
No	48(38.1)	26(31.3)		–	–	
Yes	78(61.9)	57(68.7)		–	–	

Afterwards, FEPM score and clinicopathological features were submitted to univariate Cox regression analysis as predictors. The forest plots showed that FEPM score was a significant prognostic risk factor for HCC patients, either in TCGA cohort (HR = 3.611, 95%CI: 2.741-4.758, p< 0.001) or ICGC cohort (HR = 1.754, 95%CI: 1.242-2.479, p = 0.001) (**Figures 5B, D**). Finally, the statistically significant predictors were submitted to multivariate Cox regression analysis. As shown in forest plots, FEPM score was an independent prognostic factor for HCC patients (TCGA cohort: p< 0.001; ICGC cohort: p = 0.005) (**Figures 5C, E**).

### 3.4 Analysis of ferroptosis and EMT status

**Figure 6:**
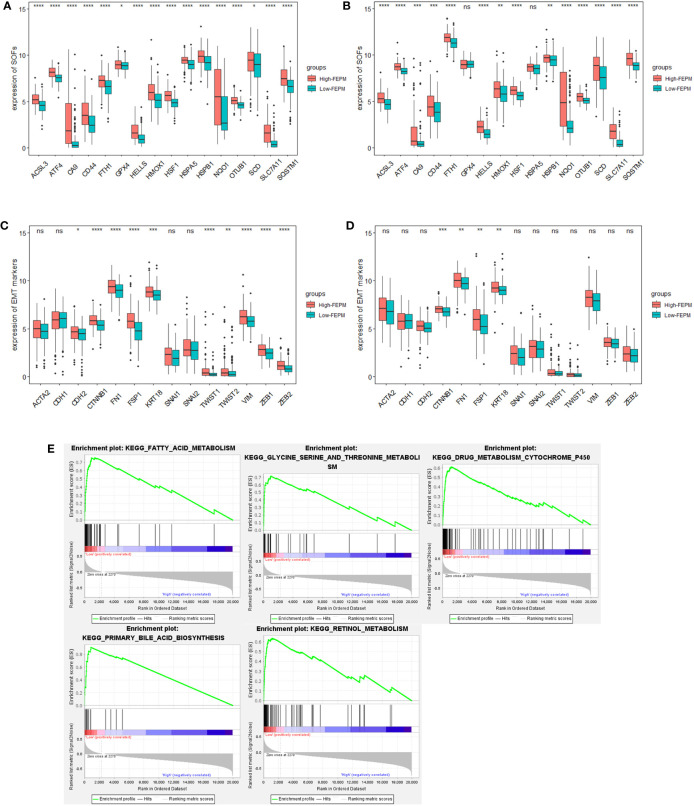
Analysis of Ferroptosis and EMT status. **(A, B)** Boxplots showing the expression profiles of SOFs in the TCGA and ICGC cohorts. **(C, D)** Boxplots showing the expression profiles of EMT markers in the TCGA and ICGC cohorts. **(E)** Pathways enriched in the low-FEPM group in the TCGA cohort. ^*^ p< 0.05; ^**^p< 0.01; ^***^p< 0.001; ^****^p< 0.0001; ns, not significant.

To analyze the ferroptosis status, we contrasted the expression profiles of suppressors of ferroptosis (SOFs) in the two subgroups. *FTH1*, *GPX4*, *HELLS*, *ATF4*, *OTUB1, CA9*, *HSPB1*, *CD44*, *HMOX1*, *SLC7A11*, *SQSTM1*, *HSF1*, *ACSL3*, *HSPA5*, *SCD*, *NQO1* are SOFs that have been thoroughly researched (29, 30). In the TCGA cohort, it was amazing to notice that the expressions of all these SOFs were significantly upregulated in the high-FEPM group (**Figure 6A**). Except for *GPX4* and *HSPA5*, SOFs also expressed at a higher level in the high-FEPM group of ICGC cohort (**Figure 6B**). In addition, the expressions of some drivers of ferroptosis (DOFs) were downregulated in the high-FEPM group, including *CDO1*, *ACO1*, *GOT1*, *MAP1LC3A* and *PEBP1* (**Supplementary Figures 1A, B**). These findings suggested that the high-FEPM group appeared to have a suppressive ferroptosis status.

GSEA analysis was accomplished in the TCGA cohort. As a result, many ferroptosis-related metabolism approaches (including primary bile acid biosynthesis, fatty acid metabolism, Glycine, serine and threonine metabolism, retinol metabolism, and cytochrome P450 activity) were significantly enriched in the low-FEPM group (**Figure 6E**).

To clarify whether FEPM could identify EMT status, we compared the expressions of a group of widely recognized epithelial markers, mesenchymal markers, and EMT transcription factors (EMT-TFs) between different groups (12, 13, 31). In the TCGA cohort, we found that the expressions of mesenchymal markers (*CDH2*, *VIM*, *FSP1*, *CTNNB1*, and *FN1*) and EMT-TFs (TWIST1, TWIST2, ZEB1, and ZEB2) were considerably increased in the high-FEPM group (**Figure 6C**). In the ICGC cohort, the high-FEPM group also showed great elevation in the expressions of mesenchymal markers (*FSP1*, *CTNNB1*, and *FN1*) (**Figure 6D**). These findings suggested that the high-FEPM group might have a more active EMT status.

### 3.5 Immunosuppressive microenvironment in the high-risk group

**Figure 7:**
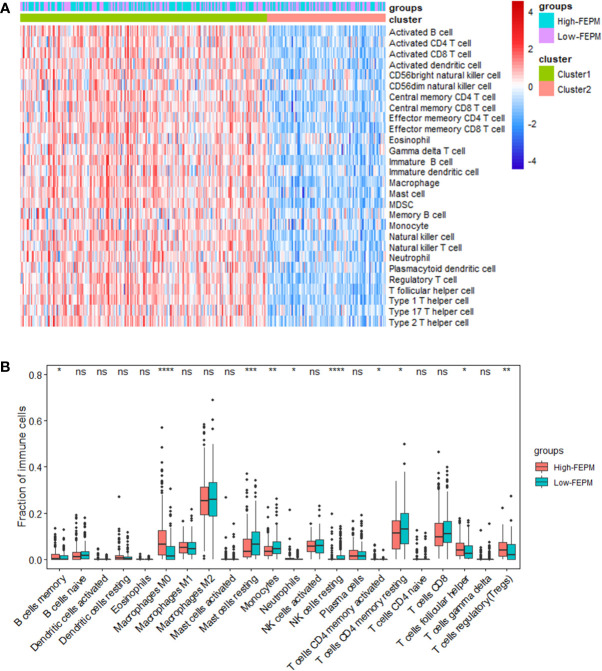
Analysis of tumor immune microenvironment. **(A)** A heatmap of ssGSEA in the TCGA cohort showing the high immune infiltration cluster (cluster 1) and the low immune infiltration cluster (cluster 2). **(B)** Comparison between the fractions of immune cells in the high-FEPM and low-FEPM groups of the TCGA cohort *via* the CIBERSORT method. ^*^ p< 0.05; ^**^p< 0.01; ^***^p< 0.001; ^****^p< 0.0001; ns, not significant.

**Figure 8:**
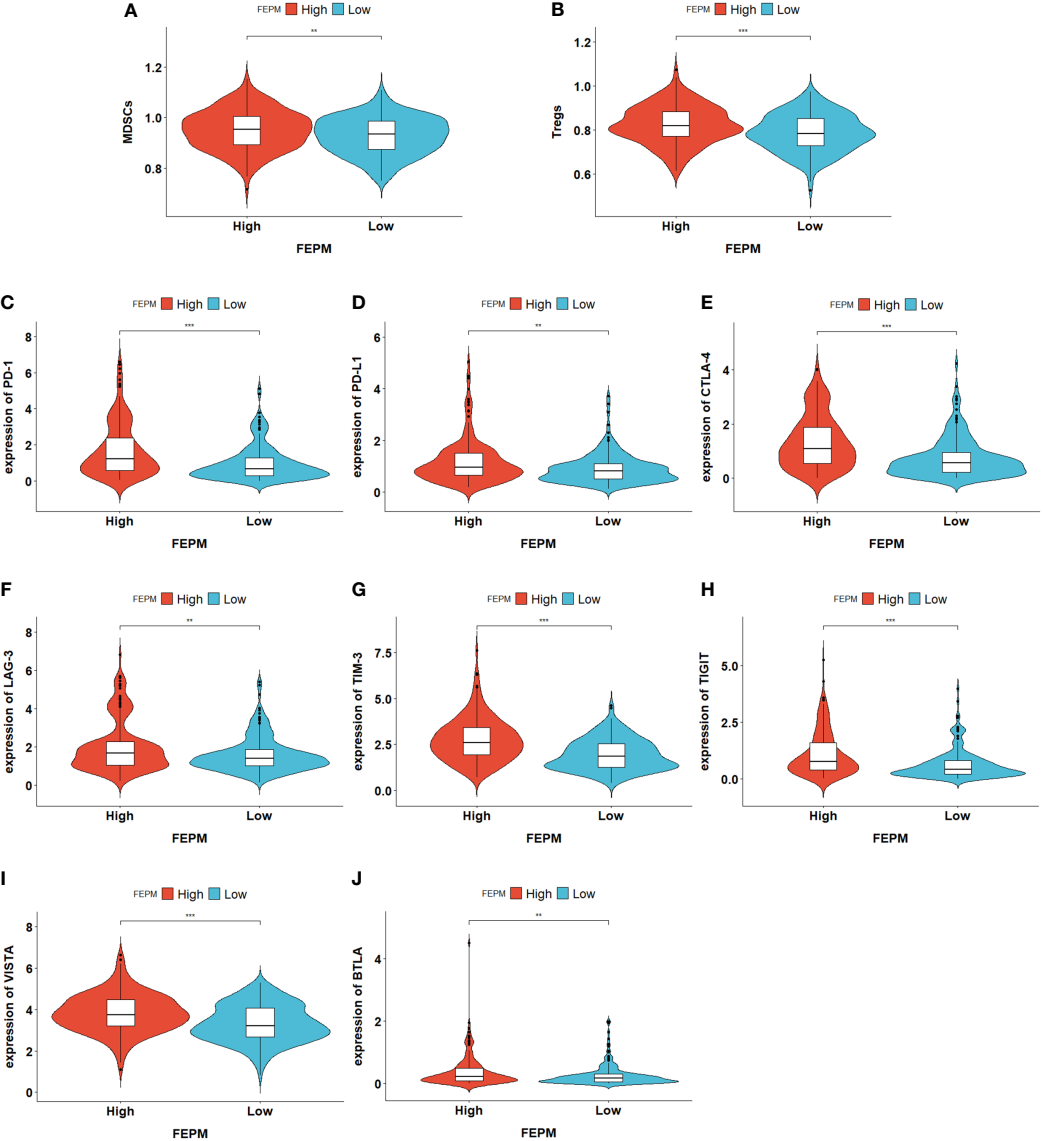
**(A, B)** Violin plots showing the upregulation of immunosuppressive cells (MDSCs and Tregs) in the high-FEPM group based on the ssGSEA enrichment scores of TCGA cohort. **(C-J)** Violin plots showing the upregulation of a group of immune checkpoints in the high-FEPM group in the TCGA cohort. ^**^p< 0.01; ^***^p< 0.001.

To find out how the FEPM reflects the tumor immune microenvironment, we computed the enrichment scores of immune cells in TCGA cohort by means of ssGSEA. Then we identified two subtypes including high immune infiltration cluster and low immune infiltration cluster *via* the method of cluster analysis (**Figure 7A**). To find out whether the high-FEPM group possessed high or low immune infiltration abundance, we performed the chi-square test. The result showed no significant statistical difference (p > 0.05) (**Supplementary Table 1**). However, we found a significant upregulation of immunosuppressive cells, such as myeloid-derived suppressor cells (MDSCs), and regulatory T cells (Tregs) (**Figures 8A, B**). Besides, a group of immune checkpoints including CTLA-4, PD-L1, PD-1, BTLA, LAG-3, TIGIT, TIM-3 and VISTA highly expressed in the high-FEPM group (**Figures 8C–J**). CIBERSORT analysis showed higher infiltration levels of neutrophils, macrophages M0, memory B cells, follicular helper T cells, regulatory T cells (Tregs), and activated memory CD4 T cells in the high-FEPM group. The infiltration levels of resting mast cells, monocytes, resting NK cells, and resting memory CD4 T cells were higher in the low-FEPM group (**Figure 7B**).

### 3.6 The nomogram performs well for prognosis prediction in the two cohorts

**Figure 9:**
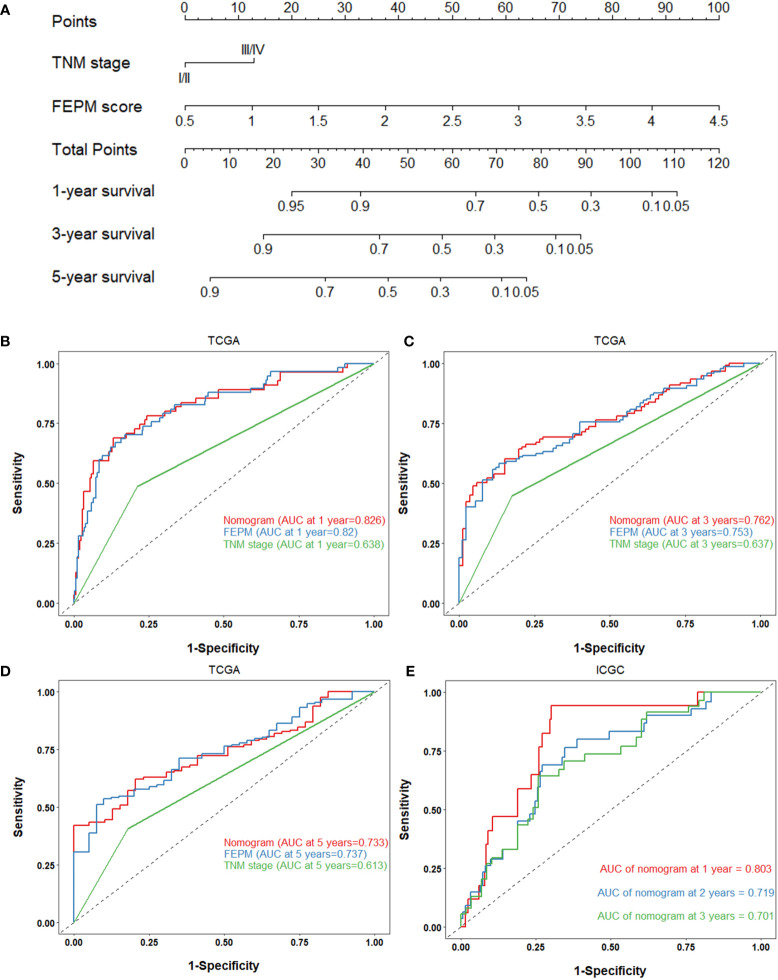
**(A)** Nomogram based on FEPM score and TNM stage. **(B-D)** ROC curves for the nomogram, FEPM score, and TNM stage in the TCGA cohort at 1, 3, and 5 years. **(E)** ROC curves for the nomogram in the ICGC cohort at 1, 2, and 3 years.

**Figure 10:**
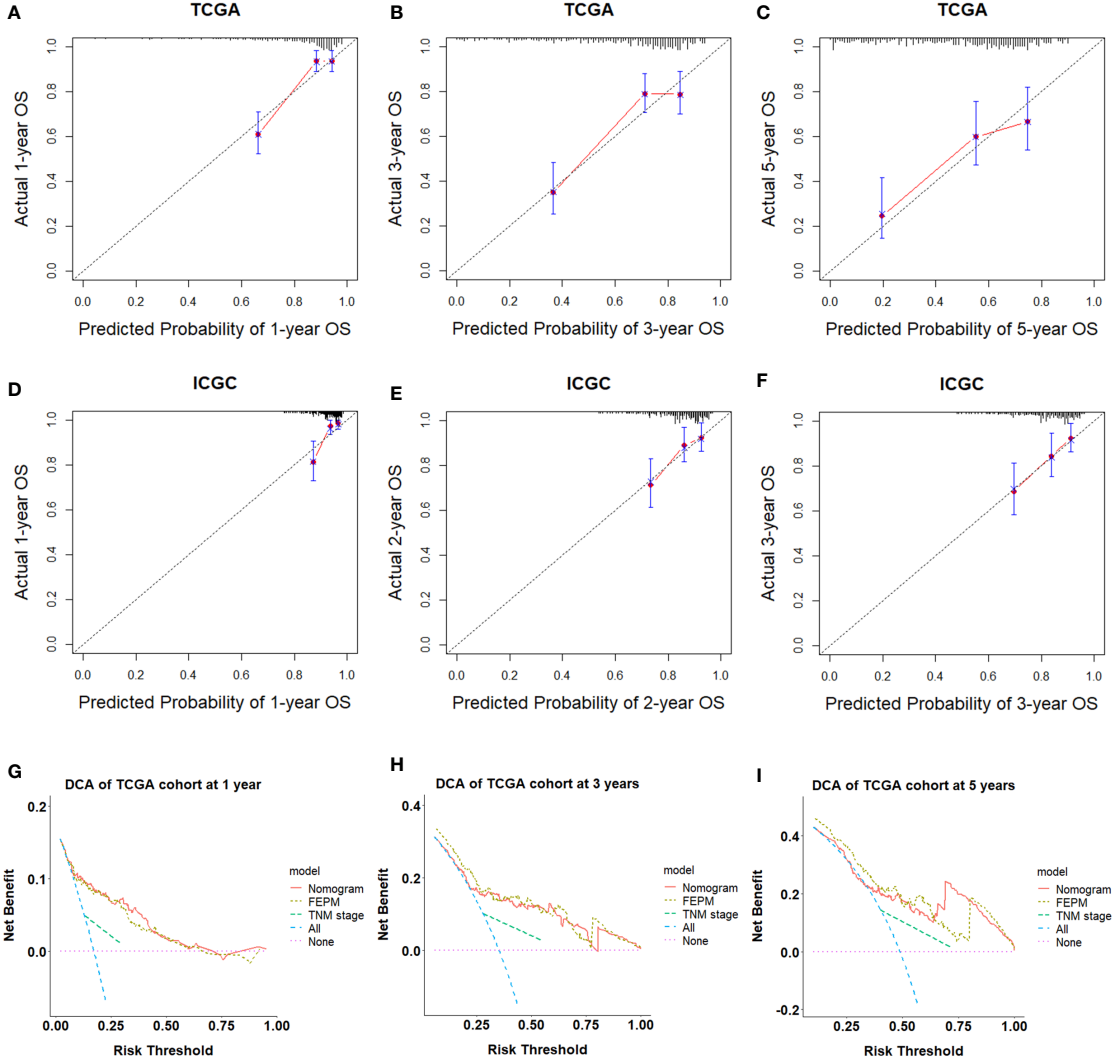
**(A-F)** Calibration curves showed the nomogram had great consistency. **(G-I)** Decision curve analysis showed that the nomogram could provide clinical benefits within a wide range of thresholds.

To improve the prediction ability and facilitate clinical application, we constructed a nomogram and appraised its performance in the TCGA and ICGC cohorts. The nomogram enrolled two independent prognostic factors including FEPM score and TNM stage (**Figure 9A**). According to the time-dependent ROC curves, AUCs of the nomogram reached 0.826 (1-year), 0.762 (3-years), and 0.733 (5-years) respectively in the TCGA cohort (**Figures 9B–D**). The AUCs at 1, 2, and 3 years reached 0.803, 0.719 and 0.701 in the ICGC cohort, indicating desirable sensitivity and specificity (**Figure 9E**). The C-indexes of TCGA cohort and ICGC cohort were respectively 0.734 (95%CI: 0.683-0.785) and 0.713 (95%CI: 0.637-0.789). Calibration curves further showed that the predictive values of the nomogram were highly consistent with the actual values (**Figures 10A–F**). Decision curve analysis (DCA) showed that the nomogram could provide clinical benefits within a wide range of thresholds (**Figures 10G–I**).

## 4 Discussion

In this study, we developed a risk prediction model combining ferroptosis and EMT. Patients in the high-risk group had shorter survival times and more advanced tumor stages. Furthermore, we conducted immune infiltration analysis and established a nomogram. ROC curves, C-indexes, calibration curves and DCA analyses showed that the nomogram had a strong predictive ability and played a certain guiding role in clinical decision-making. For high-risk patients, we can increase the frequency of follow-up to monitor tumor progression and choose more aggressive treatments to benefit patients.

Consistent with the findings of two previous studies (29, 30), we discovered that patients in the high-FEPM group displayed a suppressive ferroptosis state. HSPA5, as a promoter of unfolded protein response (UPR), is beneficial to cell survival under endoplasmic reticulum stress (32). GPX4 is a key regulator of ferroptosis and lipid peroxidation (33). Studies have proved that HSPA5 can bind to GPX4 and inhibit GPX4 protein degradation, thereby inhibiting ferroptosis in pancreatic ductal adenocarcinoma (PDAC) cells and colorectal cancer (CRC) cells (34, 35). In addition, activation of PERK/ATF4/HSPA5 pathway attenuated dihydroartemisinin-induced ferroptosis in glioma cells (36). However, no significant upregulation of HSPA5 and GPX4 was observed in the high-risk group of the ICGC cohort, suggesting that the HSPA5/GPX4 pathway might not work in subgroup stratification of the ICGC cohort. GSEA results showed that biological processes such as fatty acid metabolism, amino acid metabolism, retinol metabolism, bile acid biosynthesis, and cytochrome P450 redox reaction were significantly enriched in the low-risk group. These biological processes were closely related to lipid peroxidation and reactive oxygen species (ROS) production, suggesting that the low-FEPM group presented a more active ferroptosis status.

Based on a set of EMT molecular markers, we found a more active EMT status in the high-risk group. Although mesenchymal markers and EMT-TFs were elevated in the high-risk group, epithelial markers were not significantly downregulated but highly co-expressed. Increasing evidence proved that during cancer progression, most cancer cells developed partial EMT and expressed both epithelial and mesenchymal markers, known as the so-called hybrid E/M phenotype (37). For example, tamoxifen-induced dual recombinase lineage tracing systems have found that partial EMT cells rather than full EMT cells played a major role in the metastasis of breast cancer (38). Cytokeratin 18, despite a cytoskeletal protein of epithelial cells, critically contributed to initiating TGF-β1-induced EMT (39). Besides, the co-expression of cytokeratin and vimentin in some tumor types indicated higher invasive and metastatic potential (40).

We evaluated the tumor immune microenvironment in the TCGA cohort by ssGSEA and CIBERSORT. Recently, three immunogenomic subtypes of HCC have been identified by ssGSEA, which can distinguish the prognosis of different patients (41). In this study, we also identified two subtypes of immune infiltration by ssGSEA method, representing the high and low levels of immune infiltration abundance. To our disappointment, we found that the high-FEPM group and the low-FEPM group had no significant difference in the overall immune infiltration abundance. However, the infiltration degree of immunosuppressive cells MDSCs and Tregs, together with the expression levels of a set of immune checkpoint molecules, were prominently upregulated in the high-FEPM group. In addition, the macrophages M0 was significantly accumulated in the high-FEPM group according to the CIBERSORT result. Studies have showed that macrophages M0 can polarize into M2 phenotype under the induction of some stimuli, suppressing antitumor immunity (42). Macrophages M2 are one category of the tumor associated macrophages (TAMs). GPX4 inhibitors can promote cell death in TAMs and Tregs by inducing ferroptosis, thus reversing the immunosuppressive microenvironment (43). However, the antitumor effects of activated CD8^+^Tcells will be reduced at the same time due to their vulnerability to ferroptosis. It reminds us to utilize ferroptosis inducers cautiously after evaluating the tumor immune microenvironment.

In fact, EMT is closely bound up with tumor immune suppression and immune escape. For example, Snail promoted ovarian cancer progression by upregulating CXCR2 ligands to recruit MDSCs (44). ZEB1 induced the accumulation of MDSCs by upregulating the inflammatory cytokines in breast cancer (45). Besides, EMT-TFs increased the expression of immune checkpoint ligand PD-L1 (46, 47). On the other hand, CD4 ^+^ CD25 ^+^ Tregs promoted HCC invasion by secreting high levels of TGF-β1 to induce EMT (48). TAMs could help tumor cells migrate by secreting multiple proteases (42). The feedback loop between EMT and immune suppression promoted tumor progression (15). Hence, EMT inhibitors may be effective for the improvement of immunosuppressive microenvironment.

Over the past decade, inducing ferroptosis has exhibited inestimable therapeutic potential, which attracts great interests of many researchers. For instance, researchers have found that artesunate could cooperate with sorafenib to induce ferroptosis in HCC *via* different mechanisms (49). However, the inevitable drug resistance arising from long-term use will greatly limit the efficacy of these drugs. In addition, anti-CTLA-4 and anti-PD-L1/PD-1 antibodies are well-known immunotherapies of HCC. The EMT-induced tumor immunosuppressive microenvironment will greatly reduce the response of patients to these immune checkpoint inhibitors (ICIs) (15). In this study, we identified a high-risk group of patients with suppressive ferroptosis status and active EMT status. They might be lack of response sensitivity to immunotherapy because of their immunosuppressive microenvironment. Although the 13-gene prognostic model for HCC has been developed with a strong ability of prognostic prediction, as a retrospective study, this study indeed has some inevitable selection bias. Further prospective studies of larger sample’s queue are needed prior to its clinical application. In summary, a ferroptosis-related and EMT-related prognostic model was developed and validated in HCC. The implications of ferroptosis and EMT for immunotherapy were discussed emphatically in the study. In the future, the combination of ferroptosis inducers and EMT inhibitors may be a promising treatment for HCC.

## Data availability statement

The original contributions presented in the study are included in the article/**Supplementary Material**. Further inquiries can be directed to the corresponding author.

## Author contributions

XD and ZL contributed to the study conception, design, and the whole process of article revision. Material preparation, data collection and analysis were performed by JW, SYL, CR and DG. The first draft of the manuscript was written by JW and ZL, and all authors commented on previous versions of the manuscript. The figures were made by ZL, LHL and SXL, HG, DL, and LL critically revised the manuscript. All authors contributed to the article and approved the submitted version.

## Footnotes

1
https://portal.gdc.cancer.gov/

2
https://dcc.icgc.org/

3
http://www.zhounan.org/ferrdb/legacy/index.html

4
http://dbemt.bioinfo-minzhao.org/index.html

5
http://string-db.org

6
https://gdc.cancer.gov/about-data/publications/panimmune
