# Supplementary figures and images for: Prognostic prediction and immune infiltration analysis based on ferroptosis and EMT state in hepatocellular carcinoma
Source: Front Immunol. 2022 Dec 15;13:1076045. doi: 10.3389/fimmu.2022.1076045 (PMC9797854; doi:10.3389/fimmu.2022.1076045)

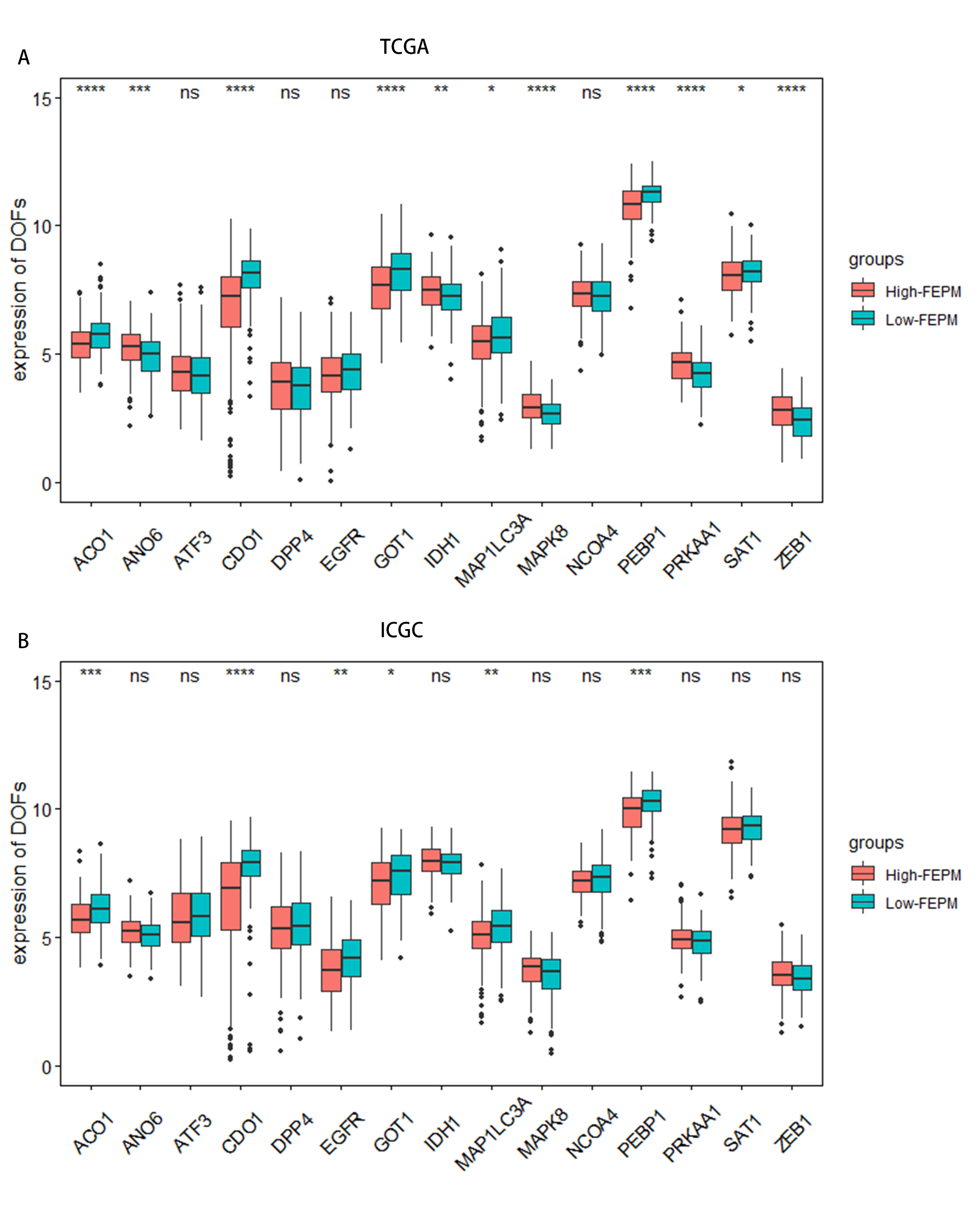

Supplement: Supplementary file 1 [file Image_1.tif]
