# Supplementary material for: Prognostic prediction and immune infiltration analysis based on ferroptosis and EMT state in hepatocellular carcinoma
Source: Front Immunol. 2022 Dec 15;13:1076045. doi: 10.3389/fimmu.2022.1076045 (PMC9797854; doi:10.3389/fimmu.2022.1076045)
Supplement: Supplementary file 2 [file Table_1.docx]

**Supplementary table 1** Chi-square test of the immune infiltration abundance in different risk groups.

| Cohort | Risk group | Immune infiltration abundance | | Total counts | Pearson Chi-Square | P value |
| --- | --- | --- | --- | --- | --- | --- |
|  |  | Low | High |  |  |  |
| TCGA | Low | 67 | 116 | 183 | 2.685 | 0.101 |
|  | High | 52 | 130 | 182 |  |  |
