# Supplementary material for: Prognostic prediction and immune infiltration analysis based on ferroptosis and EMT state in hepatocellular carcinoma
Source: Front Immunol. 2022 Dec 15;13:1076045. doi: 10.3389/fimmu.2022.1076045 (PMC9797854; doi:10.3389/fimmu.2022.1076045)
Supplement: Supplementary file 3 [file Table_2.docx]

**Supplementary table 2** The level-3 mRNA expression data files (n=424) of HCC patients from the TCGA website.

| id | links |
| --- | --- |
| 0036fcec-eaed-430b-9a23-5efb2d2cc7f2 | <https://portal.gdc.cancer.gov/files/0036fcec-eaed-430b-9a23-5efb2d2cc7f2> |
| 003ad4b1-dc1b-49bc-8ce2-d6dbd1250fee | <https://portal.gdc.cancer.gov/files/003ad4b1-dc1b-49bc-8ce2-d6dbd1250fee> |
| 00fb4b52-e6a4-4ad9-bbed-584e25851aca | <https://portal.gdc.cancer.gov/files/00fb4b52-e6a4-4ad9-bbed-584e25851aca> |
| 01e802a3-1ae8-4601-9540-d99dba62728e | https://portal.gdc.cancer.gov/files/01e802a3-1ae8-4601-9540-d99dba62728e |
| 023b8a0a-1b32-4feb-8b9d-0844dc0e7c6c | https://portal.gdc.cancer.gov/files/023b8a0a-1b32-4feb-8b9d-0844dc0e7c6c |
| 02957f01-c615-4576-8d45-473204cb284a | https://portal.gdc.cancer.gov/files/02957f01-c615-4576-8d45-473204cb284a |
| 03e3e614-324f-4471-89fc-6b134605f364 | https://portal.gdc.cancer.gov/files/03e3e614-324f-4471-89fc-6b134605f364 |
| 0444bb3d-0844-4333-97b0-e71faca95abb | https://portal.gdc.cancer.gov/files/0444bb3d-0844-4333-97b0-e71faca95abb |
| 048103cc-f5d1-4ad4-8b82-ea76715bfa53 | https://portal.gdc.cancer.gov/files/048103cc-f5d1-4ad4-8b82-ea76715bfa53 |
| 071a3c5b-9378-4cc0-ae6a-b772630822d0 | https://portal.gdc.cancer.gov/files/071a3c5b-9378-4cc0-ae6a-b772630822d0 |
| 074a7dd3-8688-4dda-9bd9-7221e32f4940 | https://portal.gdc.cancer.gov/files/074a7dd3-8688-4dda-9bd9-7221e32f4940 |
| 0829bb65-1cde-461c-b511-3649d97c62e7 | https://portal.gdc.cancer.gov/files/0829bb65-1cde-461c-b511-3649d97c62e7 |
| 08b01615-a73a-4dcb-98ec-c110b34867c3 | https://portal.gdc.cancer.gov/files/08b01615-a73a-4dcb-98ec-c110b34867c3 |
| 0940bd97-2818-48f6-a0a5-f955152409b8 | https://portal.gdc.cancer.gov/files/0940bd97-2818-48f6-a0a5-f955152409b8 |
| 095b68ff-e317-4667-b1d1-ff69254d146a | https://portal.gdc.cancer.gov/files/095b68ff-e317-4667-b1d1-ff69254d146a |
| 0bee0323-d4e5-4f61-97a3-d12e0d21c18d | https://portal.gdc.cancer.gov/files/0bee0323-d4e5-4f61-97a3-d12e0d21c18d |
| 0c11ccb8-4405-4a2a-9180-b483fb845968 | https://portal.gdc.cancer.gov/files/0c11ccb8-4405-4a2a-9180-b483fb845968 |
| 0cb37227-b328-4083-8c3c-fb8a850fc5bf | https://portal.gdc.cancer.gov/files/0cb37227-b328-4083-8c3c-fb8a850fc5bf |
| 0cf6ddfa-58d3-47b6-b2ae-885749b456c5 | https://portal.gdc.cancer.gov/files/0cf6ddfa-58d3-47b6-b2ae-885749b456c5 |
| 0d7d8f96-9010-4f69-b86a-83e8e70cdedc | https://portal.gdc.cancer.gov/files/0d7d8f96-9010-4f69-b86a-83e8e70cdedc |
| 0dbea4cc-32b6-42af-b4f0-fa0a67b05a7f | https://portal.gdc.cancer.gov/files/0dbea4cc-32b6-42af-b4f0-fa0a67b05a7f |
| 0f2270a9-bd32-4834-82f1-24197db62398 | https://portal.gdc.cancer.gov/files/0f2270a9-bd32-4834-82f1-24197db62398 |
| 0f6b6a94-1881-45a2-a6ea-a70483f379e6 | https://portal.gdc.cancer.gov/files/0f6b6a94-1881-45a2-a6ea-a70483f379e6 |
| 1080a7ed-8a78-43fc-a962-35127d9db655 | https://portal.gdc.cancer.gov/files/1080a7ed-8a78-43fc-a962-35127d9db655 |
| 1081e397-a17c-42f5-9f04-b937361441df | https://portal.gdc.cancer.gov/files/1081e397-a17c-42f5-9f04-b937361441df |
| 1086061d-5910-4b35-be67-c69836917d43 | https://portal.gdc.cancer.gov/files/1086061d-5910-4b35-be67-c69836917d43 |
| 11e8ec14-c489-4c66-b387-fea14555a3f3 | https://portal.gdc.cancer.gov/files/11e8ec14-c489-4c66-b387-fea14555a3f3 |
| 1314e20d-d48f-4c6c-b208-897bbf4cc147 | https://portal.gdc.cancer.gov/files/1314e20d-d48f-4c6c-b208-897bbf4cc147 |
| 15be9837-f3e7-45eb-8822-e98341e80362 | https://portal.gdc.cancer.gov/files/15be9837-f3e7-45eb-8822-e98341e80362 |
| 15fcbb62-7e0c-4b85-b233-643a178c1b5c | https://portal.gdc.cancer.gov/files/15fcbb62-7e0c-4b85-b233-643a178c1b5c |
| 18ec5ded-6efc-4514-bbbe-c6df897b3e08 | https://portal.gdc.cancer.gov/files/18ec5ded-6efc-4514-bbbe-c6df897b3e08 |
| 19533c53-3be7-4ede-bf65-d805e82aaf2b | https://portal.gdc.cancer.gov/files/19533c53-3be7-4ede-bf65-d805e82aaf2b |
| 19697a4b-519a-4b54-8bbe-74e9a2d28df2 | https://portal.gdc.cancer.gov/files/19697a4b-519a-4b54-8bbe-74e9a2d28df2 |
| 197e7163-1a47-4712-990a-645bd39507fd | https://portal.gdc.cancer.gov/files/197e7163-1a47-4712-990a-645bd39507fd |
| 1994b978-6d9f-4f05-b88d-a6cebeabd19c | https://portal.gdc.cancer.gov/files/1994b978-6d9f-4f05-b88d-a6cebeabd19c |
| 1c6335e8-4ab5-483e-a3b2-b1c7e9ffdb93 | https://portal.gdc.cancer.gov/files/1c6335e8-4ab5-483e-a3b2-b1c7e9ffdb93 |
| 1c6ab128-b7bd-4a6c-8e5d-8cd3d154f802 | https://portal.gdc.cancer.gov/files/1c6ab128-b7bd-4a6c-8e5d-8cd3d154f802 |
| 1cc916ad-8d93-4be4-a6a5-95c411cc57a9 | https://portal.gdc.cancer.gov/files/1cc916ad-8d93-4be4-a6a5-95c411cc57a9 |
| 1ce43195-215f-41ac-87d3-146e942bceae | https://portal.gdc.cancer.gov/files/1ce43195-215f-41ac-87d3-146e942bceae |
| 1d046beb-d3fc-474b-aedd-04249e061e71 | https://portal.gdc.cancer.gov/files/1d046beb-d3fc-474b-aedd-04249e061e71 |
| 1e1587e8-8938-4578-8529-0831573a9fe1 | https://portal.gdc.cancer.gov/files/1e1587e8-8938-4578-8529-0831573a9fe1 |
| 1e65110a-b37b-42e6-8f99-b96116fdac71 | https://portal.gdc.cancer.gov/files/1e65110a-b37b-42e6-8f99-b96116fdac71 |
| 1f63ae9f-5b3d-4367-aba8-d6222cc05667 | https://portal.gdc.cancer.gov/files/1f63ae9f-5b3d-4367-aba8-d6222cc05667 |
| 206524fb-7569-4503-a482-f41e9137d856 | https://portal.gdc.cancer.gov/files/206524fb-7569-4503-a482-f41e9137d856 |
| 2157c1f3-890e-4f10-a48d-dbe80e021ecc | https://portal.gdc.cancer.gov/files/2157c1f3-890e-4f10-a48d-dbe80e021ecc |
| 21c5a84a-877e-42ac-aabf-30eb7d6259de | https://portal.gdc.cancer.gov/files/21c5a84a-877e-42ac-aabf-30eb7d6259de |
| 21f8d42a-a743-49d4-a39b-473ad03f5ce8 | https://portal.gdc.cancer.gov/files/21f8d42a-a743-49d4-a39b-473ad03f5ce8 |
| 228b84dd-0200-4c42-99de-cc9246428d80 | https://portal.gdc.cancer.gov/files/228b84dd-0200-4c42-99de-cc9246428d80 |
| 23814830-d159-432d-adbc-d834cc5eb4fd | https://portal.gdc.cancer.gov/files/23814830-d159-432d-adbc-d834cc5eb4fd |
| 263e5d51-be7b-453e-9e83-e8f72fe8e103 | https://portal.gdc.cancer.gov/files/263e5d51-be7b-453e-9e83-e8f72fe8e103 |
| 26572c76-a5c1-40d8-988c-126df7987e14 | https://portal.gdc.cancer.gov/files/26572c76-a5c1-40d8-988c-126df7987e14 |
| 2675b7a5-87fb-481c-a09f-00116663a0a3 | https://portal.gdc.cancer.gov/files/2675b7a5-87fb-481c-a09f-00116663a0a3 |
| 2689508f-8a07-4819-b5d0-e955e176a843 | https://portal.gdc.cancer.gov/files/2689508f-8a07-4819-b5d0-e955e176a843 |
| 28211bba-af07-4e9e-abcd-fcf7cc19ba02 | https://portal.gdc.cancer.gov/files/28211bba-af07-4e9e-abcd-fcf7cc19ba02 |
| 283e4191-59da-4caf-99a3-385a3ea74607 | https://portal.gdc.cancer.gov/files/283e4191-59da-4caf-99a3-385a3ea74607 |
| 28c03921-6a64-4ce6-9627-8734812e4809 | https://portal.gdc.cancer.gov/files/28c03921-6a64-4ce6-9627-8734812e4809 |
| 290ba806-3ade-4706-9c9b-10d571765187 | https://portal.gdc.cancer.gov/files/290ba806-3ade-4706-9c9b-10d571765187 |
| 293f1d34-2368-4382-809a-3b04083329ca | https://portal.gdc.cancer.gov/files/293f1d34-2368-4382-809a-3b04083329ca |
| 2958c735-c75b-4cc0-a968-ffad46dc352b | https://portal.gdc.cancer.gov/files/2958c735-c75b-4cc0-a968-ffad46dc352b |
| 2a816d7c-2cec-4c58-81c0-6fd32d3c3886 | https://portal.gdc.cancer.gov/files/2a816d7c-2cec-4c58-81c0-6fd32d3c3886 |
| 2a986418-6f8a-4c4c-93a1-5e1abf69057b | https://portal.gdc.cancer.gov/files/2a986418-6f8a-4c4c-93a1-5e1abf69057b |
| 2af41f58-7487-493b-9839-cc8719adf8d0 | https://portal.gdc.cancer.gov/files/2af41f58-7487-493b-9839-cc8719adf8d0 |
| 2c2f0616-1fee-4f6d-adf0-64d5557b2d7a | https://portal.gdc.cancer.gov/files/2c2f0616-1fee-4f6d-adf0-64d5557b2d7a |
| 2c4002ec-6a46-4ca1-a227-e656ad54a631 | https://portal.gdc.cancer.gov/files/2c4002ec-6a46-4ca1-a227-e656ad54a631 |
| 2d17c5ed-0229-4dc4-a876-67c8a2f7745a | https://portal.gdc.cancer.gov/files/2d17c5ed-0229-4dc4-a876-67c8a2f7745a |
| 2e678e78-d136-41a2-9a33-b00a10fc5fa8 | https://portal.gdc.cancer.gov/files/2e678e78-d136-41a2-9a33-b00a10fc5fa8 |
| 2ea242f5-b89d-464f-a786-da61b83d4b6f | https://portal.gdc.cancer.gov/files/2ea242f5-b89d-464f-a786-da61b83d4b6f |
| 2fa3c211-314a-4a2d-a15a-02335262bbc4 | https://portal.gdc.cancer.gov/files/2fa3c211-314a-4a2d-a15a-02335262bbc4 |
| 3084a8bb-af8b-43b0-9c1e-fc3e190ed782 | https://portal.gdc.cancer.gov/files/3084a8bb-af8b-43b0-9c1e-fc3e190ed782 |
| 30bec809-3ad4-4a01-a7e4-7846643d1a06 | https://portal.gdc.cancer.gov/files/30bec809-3ad4-4a01-a7e4-7846643d1a06 |
| 31855350-9ddc-4279-a0af-c5564a12e1d3 | https://portal.gdc.cancer.gov/files/31855350-9ddc-4279-a0af-c5564a12e1d3 |
| 32168e04-7f7d-4be9-9e68-cd6d04bcebd6 | https://portal.gdc.cancer.gov/files/32168e04-7f7d-4be9-9e68-cd6d04bcebd6 |
| 33a40e22-a6df-4406-aaab-c1a04f0dfc66 | https://portal.gdc.cancer.gov/files/33a40e22-a6df-4406-aaab-c1a04f0dfc66 |
| 33e6cf0b-5046-4ad4-afb2-c0efe3dbe562 | https://portal.gdc.cancer.gov/files/33e6cf0b-5046-4ad4-afb2-c0efe3dbe562 |
| 34580e9c-83f1-4252-9e71-5b6767d75699 | https://portal.gdc.cancer.gov/files/34580e9c-83f1-4252-9e71-5b6767d75699 |
| 3483feb0-267e-49c8-a186-d2a10d8b95d6 | https://portal.gdc.cancer.gov/files/3483feb0-267e-49c8-a186-d2a10d8b95d6 |
| 34e4aff7-ee34-4534-8eac-285fde1f2eab | https://portal.gdc.cancer.gov/files/34e4aff7-ee34-4534-8eac-285fde1f2eab |
| 35b21093-a92a-45e4-bdb2-a82fb400c6ea | https://portal.gdc.cancer.gov/files/35b21093-a92a-45e4-bdb2-a82fb400c6ea |
| 35b3a2f1-d918-465e-b307-c06a71f31410 | https://portal.gdc.cancer.gov/files/35b3a2f1-d918-465e-b307-c06a71f31410 |
| 35c358f0-4afc-4bd3-a0f4-2741134d6f35 | https://portal.gdc.cancer.gov/files/35c358f0-4afc-4bd3-a0f4-2741134d6f35 |
| 35f067e5-fde1-4793-8d6b-d9ddd951b7c5 | https://portal.gdc.cancer.gov/files/35f067e5-fde1-4793-8d6b-d9ddd951b7c5 |
| 365068be-2415-462b-b3a4-418e4191274c | https://portal.gdc.cancer.gov/files/365068be-2415-462b-b3a4-418e4191274c |
| 369e78e8-f553-4fe0-88d2-908b624b6f8b | https://portal.gdc.cancer.gov/files/369e78e8-f553-4fe0-88d2-908b624b6f8b |
| 3700df53-8d95-4c95-9fea-64ea67e106b7 | https://portal.gdc.cancer.gov/files/3700df53-8d95-4c95-9fea-64ea67e106b7 |
| 370b35d8-24c2-4b73-94a2-48bef7b70ac9 | https://portal.gdc.cancer.gov/files/370b35d8-24c2-4b73-94a2-48bef7b70ac9 |
| 37610f08-0c05-425f-a5a6-967bfb02aa23 | https://portal.gdc.cancer.gov/files/37610f08-0c05-425f-a5a6-967bfb02aa23 |
| 37af3733-6068-4867-a545-39eef514dc12 | https://portal.gdc.cancer.gov/files/37af3733-6068-4867-a545-39eef514dc12 |
| 37de7b75-c885-44c8-9227-6600d37438df | https://portal.gdc.cancer.gov/files/37de7b75-c885-44c8-9227-6600d37438df |
| 381f27e4-5056-4fae-9f32-1e5d210f5d33 | https://portal.gdc.cancer.gov/files/381f27e4-5056-4fae-9f32-1e5d210f5d33 |
| 3903fc44-5102-44ab-a18e-774108846f70 | https://portal.gdc.cancer.gov/files/3903fc44-5102-44ab-a18e-774108846f70 |
| 39ceaa8f-92b2-43a5-b149-a3da8971cf57 | https://portal.gdc.cancer.gov/files/39ceaa8f-92b2-43a5-b149-a3da8971cf57 |
| 3a4353b6-a5ad-4950-be98-c0900e196144 | https://portal.gdc.cancer.gov/files/3a4353b6-a5ad-4950-be98-c0900e196144 |
| 3a826c9e-9966-4bbf-8ddb-e34f3c71381f | https://portal.gdc.cancer.gov/files/3a826c9e-9966-4bbf-8ddb-e34f3c71381f |
| 3a872621-c164-4e4a-8271-a637a726d63b | https://portal.gdc.cancer.gov/files/3a872621-c164-4e4a-8271-a637a726d63b |
| 3b16f864-d1ea-4ecf-b28d-cbce29b9bb47 | https://portal.gdc.cancer.gov/files/3b16f864-d1ea-4ecf-b28d-cbce29b9bb47 |
| 3b6b049d-65c4-4020-9ec5-5c6172ea4c4b | https://portal.gdc.cancer.gov/files/3b6b049d-65c4-4020-9ec5-5c6172ea4c4b |
| 3bb50e4a-840c-45e3-a312-b6d4a4bbf297 | https://portal.gdc.cancer.gov/files/3bb50e4a-840c-45e3-a312-b6d4a4bbf297 |
| 3c077b2a-3a89-4f08-93fa-ecf50f1f7e73 | https://portal.gdc.cancer.gov/files/3c077b2a-3a89-4f08-93fa-ecf50f1f7e73 |
| 3caa8628-d3d6-40d0-bfd1-0de2b2e9ed61 | https://portal.gdc.cancer.gov/files/3caa8628-d3d6-40d0-bfd1-0de2b2e9ed61 |
| 3e7b8842-d629-4fac-9da3-5d1c04c2a97e | https://portal.gdc.cancer.gov/files/3e7b8842-d629-4fac-9da3-5d1c04c2a97e |
| 3f1f1922-1263-4e5d-819b-2c89e8b5548d | https://portal.gdc.cancer.gov/files/3f1f1922-1263-4e5d-819b-2c89e8b5548d |
| 3f848440-1cde-4fc4-898c-088e47d48627 | https://portal.gdc.cancer.gov/files/3f848440-1cde-4fc4-898c-088e47d48627 |
| 4016e261-2cd4-4651-af30-de2cbf038a8a | https://portal.gdc.cancer.gov/files/4016e261-2cd4-4651-af30-de2cbf038a8a |
| 4075cc2e-ed70-4b2e-9520-542d18f4b797 | https://portal.gdc.cancer.gov/files/4075cc2e-ed70-4b2e-9520-542d18f4b797 |
| 40b00d5b-5a07-45c8-94d0-26fd8724dd93 | https://portal.gdc.cancer.gov/files/40b00d5b-5a07-45c8-94d0-26fd8724dd93 |
| 417b01fc-a6ad-4cff-b36d-99a6e4f349a1 | https://portal.gdc.cancer.gov/files/417b01fc-a6ad-4cff-b36d-99a6e4f349a1 |
| 426b0e75-593f-4992-b355-b751160e05a0 | https://portal.gdc.cancer.gov/files/426b0e75-593f-4992-b355-b751160e05a0 |
| 42fa0391-2d81-4453-a579-479ed12c9941 | https://portal.gdc.cancer.gov/files/42fa0391-2d81-4453-a579-479ed12c9941 |
| 4341af2d-dee1-4b4a-ad27-00858396c886 | https://portal.gdc.cancer.gov/files/4341af2d-dee1-4b4a-ad27-00858396c886 |
| 4471dc96-3a94-410f-ba82-b3846aedea59 | https://portal.gdc.cancer.gov/files/4471dc96-3a94-410f-ba82-b3846aedea59 |
| 448a4db4-c17f-4838-b864-7a776e4b5505 | https://portal.gdc.cancer.gov/files/448a4db4-c17f-4838-b864-7a776e4b5505 |
| 4718e60a-dc0a-4f8c-8af1-7067dba3aac3 | https://portal.gdc.cancer.gov/files/4718e60a-dc0a-4f8c-8af1-7067dba3aac3 |
| 472cb976-5b95-4130-bda1-243e24e2bd80 | https://portal.gdc.cancer.gov/files/472cb976-5b95-4130-bda1-243e24e2bd80 |
| 476c323f-4a20-41a3-91b3-7c9a81b4fbc1 | https://portal.gdc.cancer.gov/files/476c323f-4a20-41a3-91b3-7c9a81b4fbc1 |
| 477b84d7-1ae0-42f9-91d5-3d63f89d10d2 | https://portal.gdc.cancer.gov/files/477b84d7-1ae0-42f9-91d5-3d63f89d10d2 |
| 48d0a941-117d-472c-8a5e-533aeb9f03f2 | https://portal.gdc.cancer.gov/files/48d0a941-117d-472c-8a5e-533aeb9f03f2 |
| 4a217865-bbc4-4d8d-ba04-cb53bcd6d208 | https://portal.gdc.cancer.gov/files/4a217865-bbc4-4d8d-ba04-cb53bcd6d208 |
| 4a39b419-4c93-4c65-bdc3-075c1c067a41 | https://portal.gdc.cancer.gov/files/4a39b419-4c93-4c65-bdc3-075c1c067a41 |
| 4afc190c-2ce8-4eba-ad64-7337d187546b | https://portal.gdc.cancer.gov/files/4afc190c-2ce8-4eba-ad64-7337d187546b |
| 4bc85c3a-2187-474d-9fc7-874bad1eb196 | https://portal.gdc.cancer.gov/files/4bc85c3a-2187-474d-9fc7-874bad1eb196 |
| 4c09e3cb-39e1-4712-aba1-3cf8cdeade1b | https://portal.gdc.cancer.gov/files/4c09e3cb-39e1-4712-aba1-3cf8cdeade1b |
| 4c1beb5d-13a1-435f-bf1d-ea8015d486ff | https://portal.gdc.cancer.gov/files/4c1beb5d-13a1-435f-bf1d-ea8015d486ff |
| 4c9f5934-7014-4f10-bacb-758d02cd6584 | https://portal.gdc.cancer.gov/files/4c9f5934-7014-4f10-bacb-758d02cd6584 |
| 4d6f675d-ce11-4faf-ae1f-15cfbc2b5351 | https://portal.gdc.cancer.gov/files/4d6f675d-ce11-4faf-ae1f-15cfbc2b5351 |
| 4e34e3c0-7b98-4a4f-8889-489f87383ba4 | https://portal.gdc.cancer.gov/files/4e34e3c0-7b98-4a4f-8889-489f87383ba4 |
| 4e7b0b50-fe61-46d5-94bd-e8544f5c85d3 | https://portal.gdc.cancer.gov/files/4e7b0b50-fe61-46d5-94bd-e8544f5c85d3 |
| 4ec68bc8-3f5d-4d30-99e6-d5ddbdf9585a | https://portal.gdc.cancer.gov/files/4ec68bc8-3f5d-4d30-99e6-d5ddbdf9585a |
| 4f20bbeb-a2a5-48ef-9daf-fb41b729ccd1 | https://portal.gdc.cancer.gov/files/4f20bbeb-a2a5-48ef-9daf-fb41b729ccd1 |
| 5028edd3-a821-4511-99bf-d669c9d61723 | https://portal.gdc.cancer.gov/files/5028edd3-a821-4511-99bf-d669c9d61723 |
| 50510cac-4961-4b8c-b24b-06bfbfead355 | https://portal.gdc.cancer.gov/files/50510cac-4961-4b8c-b24b-06bfbfead355 |
| 513f448a-7c38-46d0-9f61-fec9416334cd | https://portal.gdc.cancer.gov/files/513f448a-7c38-46d0-9f61-fec9416334cd |
| 5180b82d-ce78-4d0f-8624-f7678732344b | https://portal.gdc.cancer.gov/files/5180b82d-ce78-4d0f-8624-f7678732344b |
| 51c45ad0-3a73-4561-9a2c-7a4c00de52c5 | https://portal.gdc.cancer.gov/files/51c45ad0-3a73-4561-9a2c-7a4c00de52c5 |
| 51d8982b-ade8-4cd7-83c2-bb32eeff4c8a | https://portal.gdc.cancer.gov/files/51d8982b-ade8-4cd7-83c2-bb32eeff4c8a |
| 520e80a8-33c2-4bbb-9134-67c767bd526f | https://portal.gdc.cancer.gov/files/520e80a8-33c2-4bbb-9134-67c767bd526f |
| 541260c2-5f9f-459d-9a49-df55b62fba90 | https://portal.gdc.cancer.gov/files/541260c2-5f9f-459d-9a49-df55b62fba90 |
| 5460eb0c-4844-4c74-95c7-519184822a5a | https://portal.gdc.cancer.gov/files/5460eb0c-4844-4c74-95c7-519184822a5a |
| 559f7bde-209c-4ca8-8bd7-dbac076e91d0 | https://portal.gdc.cancer.gov/files/559f7bde-209c-4ca8-8bd7-dbac076e91d0 |
| 56a82d59-00e0-4d30-8df0-8232d59e6728 | https://portal.gdc.cancer.gov/files/56a82d59-00e0-4d30-8df0-8232d59e6728 |
| 56f31145-6c4f-40f2-9f1b-2c428f1c6f6d | https://portal.gdc.cancer.gov/files/56f31145-6c4f-40f2-9f1b-2c428f1c6f6d |
| 572d387f-648a-4547-871a-f0be87a9d3ee | https://portal.gdc.cancer.gov/files/572d387f-648a-4547-871a-f0be87a9d3ee |
| 5771eb80-eb56-4014-bf86-0212b686330e | https://portal.gdc.cancer.gov/files/5771eb80-eb56-4014-bf86-0212b686330e |
| 58e4283a-b744-4871-81ec-6d18baa1a8e5 | https://portal.gdc.cancer.gov/files/58e4283a-b744-4871-81ec-6d18baa1a8e5 |
| 599b4be8-eafd-4f0a-b075-da3e7215b8f0 | https://portal.gdc.cancer.gov/files/599b4be8-eafd-4f0a-b075-da3e7215b8f0 |
| 59c0ae99-4212-4ba3-ae15-53e41b6681b6 | https://portal.gdc.cancer.gov/files/59c0ae99-4212-4ba3-ae15-53e41b6681b6 |
| 5a0d958d-f9c1-41ef-93cc-bfffb8df1f1b | https://portal.gdc.cancer.gov/files/5a0d958d-f9c1-41ef-93cc-bfffb8df1f1b |
| 5a1259f8-620d-42be-900f-65aacb6a6b43 | https://portal.gdc.cancer.gov/files/5a1259f8-620d-42be-900f-65aacb6a6b43 |
| 5a46660d-4eb2-42de-92e7-8b55f9d07a44 | https://portal.gdc.cancer.gov/files/5a46660d-4eb2-42de-92e7-8b55f9d07a44 |
| 5a9a239f-82d6-4875-970e-bc57558691b4 | https://portal.gdc.cancer.gov/files/5a9a239f-82d6-4875-970e-bc57558691b4 |
| 5b4d459d-ee3d-46a0-be38-cafa1a5fad66 | https://portal.gdc.cancer.gov/files/5b4d459d-ee3d-46a0-be38-cafa1a5fad66 |
| 5cbb4d1e-10f5-447f-b1e0-18b45c86bb98 | https://portal.gdc.cancer.gov/files/5cbb4d1e-10f5-447f-b1e0-18b45c86bb98 |
| 5d9881ac-703a-4ac6-8d77-4e995725e4a5 | https://portal.gdc.cancer.gov/files/5d9881ac-703a-4ac6-8d77-4e995725e4a5 |
| 5faa2c8d-32d4-47c3-9689-8841e48e4eff | https://portal.gdc.cancer.gov/files/5faa2c8d-32d4-47c3-9689-8841e48e4eff |
| 60332171-425b-46f5-8164-e3b1fb882036 | https://portal.gdc.cancer.gov/files/60332171-425b-46f5-8164-e3b1fb882036 |
| 606f2957-6536-46ae-9f62-d8dd7e2856d9 | https://portal.gdc.cancer.gov/files/606f2957-6536-46ae-9f62-d8dd7e2856d9 |
| 6080f1ab-a146-4ba7-8ca2-99204039f8e5 | https://portal.gdc.cancer.gov/files/6080f1ab-a146-4ba7-8ca2-99204039f8e5 |
| 6191b274-9874-4661-8b76-3e775595fc69 | https://portal.gdc.cancer.gov/files/6191b274-9874-4661-8b76-3e775595fc69 |
| 61ad124d-5315-447b-8557-49444ef18f49 | https://portal.gdc.cancer.gov/files/61ad124d-5315-447b-8557-49444ef18f49 |
| 61f028d1-e7ba-423d-b610-9cd1229d110e | https://portal.gdc.cancer.gov/files/61f028d1-e7ba-423d-b610-9cd1229d110e |
| 6226ffe4-bd87-4f0f-8b1b-c2f2baa0d59c | https://portal.gdc.cancer.gov/files/6226ffe4-bd87-4f0f-8b1b-c2f2baa0d59c |
| 634cd164-4eb6-4bb5-baef-80d23bc02cfb | https://portal.gdc.cancer.gov/files/634cd164-4eb6-4bb5-baef-80d23bc02cfb |
| 63870086-6129-4ae1-a3c4-e9ace85f1df0 | https://portal.gdc.cancer.gov/files/63870086-6129-4ae1-a3c4-e9ace85f1df0 |
| 6499e9dc-8755-47c0-845d-c570da6f908e | https://portal.gdc.cancer.gov/files/6499e9dc-8755-47c0-845d-c570da6f908e |
| 64b69a94-60cb-4888-b5a8-1076aaad827f | https://portal.gdc.cancer.gov/files/64b69a94-60cb-4888-b5a8-1076aaad827f |
| 654d7741-6628-43d7-bfbf-238e8aa87be0 | https://portal.gdc.cancer.gov/files/654d7741-6628-43d7-bfbf-238e8aa87be0 |
| 65ea5c74-595e-468f-8dc5-134a8c3ae543 | https://portal.gdc.cancer.gov/files/65ea5c74-595e-468f-8dc5-134a8c3ae543 |
| 66228099-939e-4131-8d31-98d18c51c9e9 | https://portal.gdc.cancer.gov/files/66228099-939e-4131-8d31-98d18c51c9e9 |
| 667c2d08-7a5d-44fc-adb3-31dd2dbea87f | https://portal.gdc.cancer.gov/files/667c2d08-7a5d-44fc-adb3-31dd2dbea87f |
| 66cf470a-ec42-4f85-b4b2-bf7e8055a55b | https://portal.gdc.cancer.gov/files/66cf470a-ec42-4f85-b4b2-bf7e8055a55b |
| 67078084-0785-4601-993d-b76631d58a88 | https://portal.gdc.cancer.gov/files/67078084-0785-4601-993d-b76631d58a88 |
| 678a19cb-a88c-4093-b6b5-07c63237c42b | https://portal.gdc.cancer.gov/files/678a19cb-a88c-4093-b6b5-07c63237c42b |
| 67993fc6-feaf-4468-927c-0daf9ca72aed | https://portal.gdc.cancer.gov/files/67993fc6-feaf-4468-927c-0daf9ca72aed |
| 6983f3b9-c9ba-433a-8f13-6429122ca395 | https://portal.gdc.cancer.gov/files/6983f3b9-c9ba-433a-8f13-6429122ca395 |
| 6a40d8bb-f30a-4802-8043-431551c2ca51 | https://portal.gdc.cancer.gov/files/6a40d8bb-f30a-4802-8043-431551c2ca51 |
| 6a598923-a466-455f-a6b6-3f468a1e76cb | https://portal.gdc.cancer.gov/files/6a598923-a466-455f-a6b6-3f468a1e76cb |
| 6a7615b4-460b-48ca-93de-345711844462 | https://portal.gdc.cancer.gov/files/6a7615b4-460b-48ca-93de-345711844462 |
| 6b50634f-49fe-4832-a8f5-093bedaed19c | https://portal.gdc.cancer.gov/files/6b50634f-49fe-4832-a8f5-093bedaed19c |
| 6d1cf7fd-b7e2-4770-b0ce-5823de7ba817 | https://portal.gdc.cancer.gov/files/6d1cf7fd-b7e2-4770-b0ce-5823de7ba817 |
| 6d4ab014-ce19-42f0-9ce8-e0b03ba47a86 | https://portal.gdc.cancer.gov/files/6d4ab014-ce19-42f0-9ce8-e0b03ba47a86 |
| 6e36807b-e15a-416b-bded-7202a9dab757 | https://portal.gdc.cancer.gov/files/6e36807b-e15a-416b-bded-7202a9dab757 |
| 6e42eeef-4aa8-4e27-8f2d-57c3d8401602 | https://portal.gdc.cancer.gov/files/6e42eeef-4aa8-4e27-8f2d-57c3d8401602 |
| 6e812349-35e7-44e7-ac7c-a4bc19d81fd0 | https://portal.gdc.cancer.gov/files/6e812349-35e7-44e7-ac7c-a4bc19d81fd0 |
| 6f5328d3-7a56-4da1-b2b3-fae1bc627626 | https://portal.gdc.cancer.gov/files/6f5328d3-7a56-4da1-b2b3-fae1bc627626 |
| 6f98bcbf-b39e-4c18-ba5e-3505adaac6b5 | https://portal.gdc.cancer.gov/files/6f98bcbf-b39e-4c18-ba5e-3505adaac6b5 |
| 704376d7-9bd9-4097-bb4e-60ee92241481 | https://portal.gdc.cancer.gov/files/704376d7-9bd9-4097-bb4e-60ee92241481 |
| 70aed113-9cee-4797-bd82-476a84b4b7e4 | https://portal.gdc.cancer.gov/files/70aed113-9cee-4797-bd82-476a84b4b7e4 |
| 715b5ec9-5c2c-4dac-b9f6-ba459743a377 | https://portal.gdc.cancer.gov/files/715b5ec9-5c2c-4dac-b9f6-ba459743a377 |
| 71eec830-b5e6-4a18-8b28-c8961fef01c9 | https://portal.gdc.cancer.gov/files/71eec830-b5e6-4a18-8b28-c8961fef01c9 |
| 7214d789-2919-4fa4-b8fc-660c5c160202 | https://portal.gdc.cancer.gov/files/7214d789-2919-4fa4-b8fc-660c5c160202 |
| 72b4e1c9-0c5b-4110-9650-0505cc5fce3a | https://portal.gdc.cancer.gov/files/72b4e1c9-0c5b-4110-9650-0505cc5fce3a |
| 73857f48-b515-4c00-911e-b0338b3336fa | https://portal.gdc.cancer.gov/files/73857f48-b515-4c00-911e-b0338b3336fa |
| 740bc272-2179-48e2-a801-932a3e90b6fe | https://portal.gdc.cancer.gov/files/740bc272-2179-48e2-a801-932a3e90b6fe |
| 741f648e-87f2-4c61-b1d5-41579841aa58 | https://portal.gdc.cancer.gov/files/741f648e-87f2-4c61-b1d5-41579841aa58 |
| 7445bd44-47b4-4a4d-b38f-2873def428f9 | https://portal.gdc.cancer.gov/files/7445bd44-47b4-4a4d-b38f-2873def428f9 |
| 757821dd-1fce-428d-985e-beb33d18842e | https://portal.gdc.cancer.gov/files/757821dd-1fce-428d-985e-beb33d18842e |
| 767226e6-cc1b-449d-b697-4d3ec2660006 | https://portal.gdc.cancer.gov/files/767226e6-cc1b-449d-b697-4d3ec2660006 |
| 772bdab0-f31c-441e-b79e-240fd622079b | https://portal.gdc.cancer.gov/files/772bdab0-f31c-441e-b79e-240fd622079b |
| 780b79cd-5502-455d-9df3-d20c96a26649 | https://portal.gdc.cancer.gov/files/780b79cd-5502-455d-9df3-d20c96a26649 |
| 78a0f8f9-e010-4a10-978c-94c8bb9157cd | https://portal.gdc.cancer.gov/files/78a0f8f9-e010-4a10-978c-94c8bb9157cd |
| 79087a10-f5df-4e07-9c80-ac4a43f9cc55 | https://portal.gdc.cancer.gov/files/79087a10-f5df-4e07-9c80-ac4a43f9cc55 |
| 7936688f-fdd9-4e83-adfc-785fd93559f6 | https://portal.gdc.cancer.gov/files/7936688f-fdd9-4e83-adfc-785fd93559f6 |
| 79524c66-28e9-47c9-9934-d64fe6e0ea92 | https://portal.gdc.cancer.gov/files/79524c66-28e9-47c9-9934-d64fe6e0ea92 |
| 79a6beab-c0ad-41b1-b264-e8869e284b26 | https://portal.gdc.cancer.gov/files/79a6beab-c0ad-41b1-b264-e8869e284b26 |
| 79ab15c3-648d-4e4d-8b06-5bca7fb64683 | https://portal.gdc.cancer.gov/files/79ab15c3-648d-4e4d-8b06-5bca7fb64683 |
| 7b541fbd-a378-4d59-a035-7898348807fd | https://portal.gdc.cancer.gov/files/7b541fbd-a378-4d59-a035-7898348807fd |
| 7b90b9fd-0015-47b9-9148-f040c1cfcb5a | https://portal.gdc.cancer.gov/files/7b90b9fd-0015-47b9-9148-f040c1cfcb5a |
| 7b929596-4646-4aa4-9705-d10519ef23c0 | https://portal.gdc.cancer.gov/files/7b929596-4646-4aa4-9705-d10519ef23c0 |
| 7c134e70-c906-42f8-8169-a6281ece4bf0 | https://portal.gdc.cancer.gov/files/7c134e70-c906-42f8-8169-a6281ece4bf0 |
| 7d22b54a-47ae-49de-bc78-909ac1bf4c63 | https://portal.gdc.cancer.gov/files/7d22b54a-47ae-49de-bc78-909ac1bf4c63 |
| 7da0d21d-971c-420b-9fcd-e9c86134d129 | https://portal.gdc.cancer.gov/files/7da0d21d-971c-420b-9fcd-e9c86134d129 |
| 7ddcf3fa-7d26-46e1-a710-6831df9f2e66 | https://portal.gdc.cancer.gov/files/7ddcf3fa-7d26-46e1-a710-6831df9f2e66 |
| 7e03a043-3837-476b-8183-77b9b5cebaa0 | https://portal.gdc.cancer.gov/files/7e03a043-3837-476b-8183-77b9b5cebaa0 |
| 7e63467a-79da-41b7-93f6-ad4096b87914 | https://portal.gdc.cancer.gov/files/7e63467a-79da-41b7-93f6-ad4096b87914 |
| 7fb95d95-2359-471e-aac2-edf70dae1574 | https://portal.gdc.cancer.gov/files/7fb95d95-2359-471e-aac2-edf70dae1574 |
| 8088cd3e-c3dc-45dc-8b66-637ada8ef440 | https://portal.gdc.cancer.gov/files/8088cd3e-c3dc-45dc-8b66-637ada8ef440 |
| 8177645a-a3a1-4f95-b6a1-0d2a3fd37b3c | https://portal.gdc.cancer.gov/files/8177645a-a3a1-4f95-b6a1-0d2a3fd37b3c |
| 81d640c5-ad25-4f27-881d-83195de5116a | https://portal.gdc.cancer.gov/files/81d640c5-ad25-4f27-881d-83195de5116a |
| 82dd1ddd-d629-492f-8f9c-14382a82de96 | https://portal.gdc.cancer.gov/files/82dd1ddd-d629-492f-8f9c-14382a82de96 |
| 8336ea51-7b0f-4fc5-b684-94c03d640da9 | https://portal.gdc.cancer.gov/files/8336ea51-7b0f-4fc5-b684-94c03d640da9 |
| 83fae235-032f-428c-850c-1e5bda92339c | https://portal.gdc.cancer.gov/files/83fae235-032f-428c-850c-1e5bda92339c |
| 845b36d6-842d-4467-a59e-b7d9272a47b1 | https://portal.gdc.cancer.gov/files/845b36d6-842d-4467-a59e-b7d9272a47b1 |
| 84f52c89-db34-4a63-9491-a11cebe8fcde | https://portal.gdc.cancer.gov/files/84f52c89-db34-4a63-9491-a11cebe8fcde |
| 853874dd-4482-4dd2-aa8f-a0a43562928d | https://portal.gdc.cancer.gov/files/853874dd-4482-4dd2-aa8f-a0a43562928d |
| 858fa98b-752a-4005-a292-1c5c7153ceb5 | https://portal.gdc.cancer.gov/files/858fa98b-752a-4005-a292-1c5c7153ceb5 |
| 86170815-fb54-494b-95e7-4657946f2d17 | https://portal.gdc.cancer.gov/files/86170815-fb54-494b-95e7-4657946f2d17 |
| 866b6563-f889-4dfe-8965-092c7b925af8 | https://portal.gdc.cancer.gov/files/866b6563-f889-4dfe-8965-092c7b925af8 |
| 86c7f61d-ad00-429f-9343-300105243838 | https://portal.gdc.cancer.gov/files/86c7f61d-ad00-429f-9343-300105243838 |
| 8710b79a-f5be-4426-82ea-3827099c2c54 | https://portal.gdc.cancer.gov/files/8710b79a-f5be-4426-82ea-3827099c2c54 |
| 8842358a-057c-4a49-8d0c-a2c72e2a042b | https://portal.gdc.cancer.gov/files/8842358a-057c-4a49-8d0c-a2c72e2a042b |
| 886fbdb4-b0a8-481b-9721-5e63956caebd | https://portal.gdc.cancer.gov/files/886fbdb4-b0a8-481b-9721-5e63956caebd |
| 8956b891-ff72-4207-a509-866e7bb62cce | https://portal.gdc.cancer.gov/files/8956b891-ff72-4207-a509-866e7bb62cce |
| 89a6bdeb-5056-4d6c-8c60-11763bc29da6 | https://portal.gdc.cancer.gov/files/89a6bdeb-5056-4d6c-8c60-11763bc29da6 |
| 8a284492-4c97-40cc-974c-0f1941ff0fe9 | https://portal.gdc.cancer.gov/files/8a284492-4c97-40cc-974c-0f1941ff0fe9 |
| 8a76a1a8-dbe1-4058-a6d3-fceb2d325b16 | https://portal.gdc.cancer.gov/files/8a76a1a8-dbe1-4058-a6d3-fceb2d325b16 |
| 8c410ea8-1847-4c05-9f5c-33771f62c498 | https://portal.gdc.cancer.gov/files/8c410ea8-1847-4c05-9f5c-33771f62c498 |
| 8c797f41-9dea-4121-8dc3-8b1fa10503a7 | https://portal.gdc.cancer.gov/files/8c797f41-9dea-4121-8dc3-8b1fa10503a7 |
| 8ca35f8b-9a11-4f07-933d-91d5feb0a069 | https://portal.gdc.cancer.gov/files/8ca35f8b-9a11-4f07-933d-91d5feb0a069 |
| 8d5d54ce-a265-4de5-86c7-73c38ecb2ff7 | https://portal.gdc.cancer.gov/files/8d5d54ce-a265-4de5-86c7-73c38ecb2ff7 |
| 8db54cb1-0635-4ce2-b51a-f5e9b29c5f4f | https://portal.gdc.cancer.gov/files/8db54cb1-0635-4ce2-b51a-f5e9b29c5f4f |
| 8f2fb555-9a2a-43a6-9b28-91cfed089bb8 | https://portal.gdc.cancer.gov/files/8f2fb555-9a2a-43a6-9b28-91cfed089bb8 |
| 8ff0d081-c3b0-4d26-9ace-e7903a9b16eb | https://portal.gdc.cancer.gov/files/8ff0d081-c3b0-4d26-9ace-e7903a9b16eb |
| 902a4484-1b5a-4dd6-bbf3-ad57b6663f5a | https://portal.gdc.cancer.gov/files/902a4484-1b5a-4dd6-bbf3-ad57b6663f5a |
| 9055c145-8bca-4056-b43e-7899539333f6 | https://portal.gdc.cancer.gov/files/9055c145-8bca-4056-b43e-7899539333f6 |
| 918ddfe3-82a0-4b47-869e-d249e99d8ee4 | https://portal.gdc.cancer.gov/files/918ddfe3-82a0-4b47-869e-d249e99d8ee4 |
| 9196e9a3-dc4e-45b3-9c6a-6efb38f1a029 | https://portal.gdc.cancer.gov/files/9196e9a3-dc4e-45b3-9c6a-6efb38f1a029 |
| 91a6115e-5869-43b2-a7ff-059ad0235071 | https://portal.gdc.cancer.gov/files/91a6115e-5869-43b2-a7ff-059ad0235071 |
| 922f342f-27af-4934-9760-79d7fa0f04ce | https://portal.gdc.cancer.gov/files/922f342f-27af-4934-9760-79d7fa0f04ce |
| 92b4c0e6-a0e0-4bd9-998d-422da266e6fd | https://portal.gdc.cancer.gov/files/92b4c0e6-a0e0-4bd9-998d-422da266e6fd |
| 932a3fef-7955-4246-88c7-c6579691501d | https://portal.gdc.cancer.gov/files/932a3fef-7955-4246-88c7-c6579691501d |
| 95158561-ffa4-4cef-bc12-9ef0368c1f10 | https://portal.gdc.cancer.gov/files/95158561-ffa4-4cef-bc12-9ef0368c1f10 |
| 951d3bd1-e7d7-46f0-a08a-e6214d2f5910 | https://portal.gdc.cancer.gov/files/951d3bd1-e7d7-46f0-a08a-e6214d2f5910 |
| 954f3c93-7105-4ace-9c1a-f8a650179ec0 | https://portal.gdc.cancer.gov/files/954f3c93-7105-4ace-9c1a-f8a650179ec0 |
| 95b4b176-edc6-4c7a-90a3-f3a957e7d77a | https://portal.gdc.cancer.gov/files/95b4b176-edc6-4c7a-90a3-f3a957e7d77a |
| 96a14578-77fc-44f0-ba46-9cba2a214a8e | https://portal.gdc.cancer.gov/files/96a14578-77fc-44f0-ba46-9cba2a214a8e |
| 970ec9cb-0e84-44b7-adf0-006f5957793a | https://portal.gdc.cancer.gov/files/970ec9cb-0e84-44b7-adf0-006f5957793a |
| 97549552-8532-4593-a0dc-44d64f417bbb | https://portal.gdc.cancer.gov/files/97549552-8532-4593-a0dc-44d64f417bbb |
| 9784c055-2da9-4720-8df8-115b5f7c4928 | https://portal.gdc.cancer.gov/files/9784c055-2da9-4720-8df8-115b5f7c4928 |
| 97a1f82c-7e86-441e-9f95-6a8e30161e51 | https://portal.gdc.cancer.gov/files/97a1f82c-7e86-441e-9f95-6a8e30161e51 |
| 991558d8-6ea1-4689-a3fc-ba6172b21828 | https://portal.gdc.cancer.gov/files/991558d8-6ea1-4689-a3fc-ba6172b21828 |
| 9967f8e8-1445-46bc-9c4f-c32f43705546 | https://portal.gdc.cancer.gov/files/9967f8e8-1445-46bc-9c4f-c32f43705546 |
| 9973d6b0-942f-4726-8ac3-ed0ba00f6bd3 | https://portal.gdc.cancer.gov/files/9973d6b0-942f-4726-8ac3-ed0ba00f6bd3 |
| 99871313-a35c-4b0b-b22d-a429a38a7fe5 | https://portal.gdc.cancer.gov/files/99871313-a35c-4b0b-b22d-a429a38a7fe5 |
| 9a37d6a0-6635-4746-928e-78e0d92fd060 | https://portal.gdc.cancer.gov/files/9a37d6a0-6635-4746-928e-78e0d92fd060 |
| 9a764e34-3125-4e05-876a-0b7ca4ce4e62 | https://portal.gdc.cancer.gov/files/9a764e34-3125-4e05-876a-0b7ca4ce4e62 |
| 9a82faa3-37c0-4951-ac43-2c04f2bbf437 | https://portal.gdc.cancer.gov/files/9a82faa3-37c0-4951-ac43-2c04f2bbf437 |
| 9bc77be6-bf4d-4aa0-a3e7-90bc1f9ab087 | https://portal.gdc.cancer.gov/files/9bc77be6-bf4d-4aa0-a3e7-90bc1f9ab087 |
| 9cc0d8b4-652a-4d7b-8821-46dc1442097e | https://portal.gdc.cancer.gov/files/9cc0d8b4-652a-4d7b-8821-46dc1442097e |
| 9d333f19-a52e-4218-8ae8-b5876d7839d2 | https://portal.gdc.cancer.gov/files/9d333f19-a52e-4218-8ae8-b5876d7839d2 |
| 9e4fda92-023e-4405-b5ed-6d57fa2b5b8a | https://portal.gdc.cancer.gov/files/9e4fda92-023e-4405-b5ed-6d57fa2b5b8a |
| 9e88b031-abcc-43fb-b49a-5debab1276b5 | https://portal.gdc.cancer.gov/files/9e88b031-abcc-43fb-b49a-5debab1276b5 |
| 9ebb875a-484b-4b77-aa66-2f727cb3eb7a | https://portal.gdc.cancer.gov/files/9ebb875a-484b-4b77-aa66-2f727cb3eb7a |
| 9ece0f6e-7b10-478f-8644-0f4198f2c09c | https://portal.gdc.cancer.gov/files/9ece0f6e-7b10-478f-8644-0f4198f2c09c |
| 9f2f2efc-0c29-4c3e-82aa-8b2755f237f9 | https://portal.gdc.cancer.gov/files/9f2f2efc-0c29-4c3e-82aa-8b2755f237f9 |
| 9f3e5801-6094-4439-b993-10bba8921da8 | https://portal.gdc.cancer.gov/files/9f3e5801-6094-4439-b993-10bba8921da8 |
| 9fa7d342-2062-4a94-8cdf-928b86b98c5b | https://portal.gdc.cancer.gov/files/9fa7d342-2062-4a94-8cdf-928b86b98c5b |
| a22db950-b799-4529-b972-4340641ebcf2 | https://portal.gdc.cancer.gov/files/a22db950-b799-4529-b972-4340641ebcf2 |
| a231db46-6db3-43ef-8209-22f5c3c013d6 | https://portal.gdc.cancer.gov/files/a231db46-6db3-43ef-8209-22f5c3c013d6 |
| a4634aa3-6ace-473b-8f60-132d32997ce3 | https://portal.gdc.cancer.gov/files/a4634aa3-6ace-473b-8f60-132d32997ce3 |
| a53b8623-b659-446a-ab87-65b6361a17cd | https://portal.gdc.cancer.gov/files/a53b8623-b659-446a-ab87-65b6361a17cd |
| a68f63db-0ddc-4dea-87a9-a56eabaefb65 | https://portal.gdc.cancer.gov/files/a68f63db-0ddc-4dea-87a9-a56eabaefb65 |
| a76565ca-2a44-4ce0-8139-40c00ecdf84a | https://portal.gdc.cancer.gov/files/a76565ca-2a44-4ce0-8139-40c00ecdf84a |
| a7b0c166-d148-41cb-8242-54d8995dac87 | https://portal.gdc.cancer.gov/files/a7b0c166-d148-41cb-8242-54d8995dac87 |
| a7c4c791-8baf-49db-998f-19898bfac217 | https://portal.gdc.cancer.gov/files/a7c4c791-8baf-49db-998f-19898bfac217 |
| a8355223-868d-4341-be88-0af16acc19d3 | https://portal.gdc.cancer.gov/files/a8355223-868d-4341-be88-0af16acc19d3 |
| a9cfbc5e-776c-4865-be20-d26741ce5c78 | https://portal.gdc.cancer.gov/files/a9cfbc5e-776c-4865-be20-d26741ce5c78 |
| a9dbf9fe-fef3-4cd7-9bb2-dcc26628566f | https://portal.gdc.cancer.gov/files/a9dbf9fe-fef3-4cd7-9bb2-dcc26628566f |
| a9ebd2a0-200c-4be1-8fc2-26d5408ee04a | https://portal.gdc.cancer.gov/files/a9ebd2a0-200c-4be1-8fc2-26d5408ee04a |
| aa987424-2d82-42ae-a319-10b36194675c | https://portal.gdc.cancer.gov/files/aa987424-2d82-42ae-a319-10b36194675c |
| ac2c8fb2-a069-41ef-9daa-b10faf870149 | https://portal.gdc.cancer.gov/files/ac2c8fb2-a069-41ef-9daa-b10faf870149 |
| ac431eeb-97c7-4c44-aabe-350bda67d59d | https://portal.gdc.cancer.gov/files/ac431eeb-97c7-4c44-aabe-350bda67d59d |
| ac75e490-0291-4133-92aa-ac4d330f58cf | https://portal.gdc.cancer.gov/files/ac75e490-0291-4133-92aa-ac4d330f58cf |
| ad30a245-b70d-44de-b3bf-b4dc4cbc2d18 | https://portal.gdc.cancer.gov/files/ad30a245-b70d-44de-b3bf-b4dc4cbc2d18 |
| aeab8782-8817-494f-8566-3fde7d8ed88d | https://portal.gdc.cancer.gov/files/aeab8782-8817-494f-8566-3fde7d8ed88d |
| afd5435d-07a2-4c95-b2b6-4a02c2a79d9c | https://portal.gdc.cancer.gov/files/afd5435d-07a2-4c95-b2b6-4a02c2a79d9c |
| b01c07c1-bb94-41fe-8bab-f00b40105709 | https://portal.gdc.cancer.gov/files/b01c07c1-bb94-41fe-8bab-f00b40105709 |
| b0ab5d25-e993-48fe-b538-699300a90ef1 | https://portal.gdc.cancer.gov/files/b0ab5d25-e993-48fe-b538-699300a90ef1 |
| b17f1eb3-14c8-45da-a580-59a5e77d9a33 | https://portal.gdc.cancer.gov/files/b17f1eb3-14c8-45da-a580-59a5e77d9a33 |
| b1d5a388-ec54-4ffb-9f5a-105faeff104f | https://portal.gdc.cancer.gov/files/b1d5a388-ec54-4ffb-9f5a-105faeff104f |
| b242b4fc-5d69-42c7-9a07-bdd47f4668cf | https://portal.gdc.cancer.gov/files/b242b4fc-5d69-42c7-9a07-bdd47f4668cf |
| b2465eaf-7eeb-4203-aefd-beebca8a1fea | https://portal.gdc.cancer.gov/files/b2465eaf-7eeb-4203-aefd-beebca8a1fea |
| b2580f86-7417-4ef2-901f-0f4011119ed8 | https://portal.gdc.cancer.gov/files/b2580f86-7417-4ef2-901f-0f4011119ed8 |
| b2646119-db5e-4591-827d-ad38195b822e | https://portal.gdc.cancer.gov/files/b2646119-db5e-4591-827d-ad38195b822e |
| b3d3844d-00ce-415e-83a5-936804ffa66c | https://portal.gdc.cancer.gov/files/b3d3844d-00ce-415e-83a5-936804ffa66c |
| b4b878d7-e613-41db-b33d-2679036a30c5 | https://portal.gdc.cancer.gov/files/b4b878d7-e613-41db-b33d-2679036a30c5 |
| b5ce86e3-f0ee-45c0-bab8-30ad531e3fec | https://portal.gdc.cancer.gov/files/b5ce86e3-f0ee-45c0-bab8-30ad531e3fec |
| b6ac2916-a8b3-4415-9b98-eb0e5a6e58af | https://portal.gdc.cancer.gov/files/b6ac2916-a8b3-4415-9b98-eb0e5a6e58af |
| ba134c1a-820f-4507-973e-7000a815ebc0 | https://portal.gdc.cancer.gov/files/ba134c1a-820f-4507-973e-7000a815ebc0 |
| ba700885-85dc-47fb-b8fb-c5af6b8284e9 | https://portal.gdc.cancer.gov/files/ba700885-85dc-47fb-b8fb-c5af6b8284e9 |
| ba8582e9-496b-42d8-8eca-189d3369bce7 | https://portal.gdc.cancer.gov/files/ba8582e9-496b-42d8-8eca-189d3369bce7 |
| bae83cb3-fb86-4648-802d-504d2e110c1e | https://portal.gdc.cancer.gov/files/bae83cb3-fb86-4648-802d-504d2e110c1e |
| bb642fb4-07dd-44b3-b5b5-3f3d6976d677 | https://portal.gdc.cancer.gov/files/bb642fb4-07dd-44b3-b5b5-3f3d6976d677 |
| bc3b8a18-5d9b-4e2d-ab82-05fb36991a2e | https://portal.gdc.cancer.gov/files/bc3b8a18-5d9b-4e2d-ab82-05fb36991a2e |
| bca5a800-5af4-437d-9ce4-7571fb046ba5 | https://portal.gdc.cancer.gov/files/bca5a800-5af4-437d-9ce4-7571fb046ba5 |
| bd469f11-1e6e-4ae5-9da4-09fe0fcdde41 | https://portal.gdc.cancer.gov/files/bd469f11-1e6e-4ae5-9da4-09fe0fcdde41 |
| bdd04bd5-dd6e-4078-a149-97ade32ba8dc | https://portal.gdc.cancer.gov/files/bdd04bd5-dd6e-4078-a149-97ade32ba8dc |
| be0308e2-3a8c-4691-8b4d-ea9459255d8c | https://portal.gdc.cancer.gov/files/be0308e2-3a8c-4691-8b4d-ea9459255d8c |
| be7803e0-0af4-43d1-8cc3-0c309c44a275 | https://portal.gdc.cancer.gov/files/be7803e0-0af4-43d1-8cc3-0c309c44a275 |
| bf7f888f-1eb2-4452-98fa-884499cc7ea7 | https://portal.gdc.cancer.gov/files/bf7f888f-1eb2-4452-98fa-884499cc7ea7 |
| c00ab5e4-51b3-4dc3-ae27-106496e90687 | https://portal.gdc.cancer.gov/files/c00ab5e4-51b3-4dc3-ae27-106496e90687 |
| c04adf74-2502-451b-ad91-13ebbceb2383 | https://portal.gdc.cancer.gov/files/c04adf74-2502-451b-ad91-13ebbceb2383 |
| c144fb4d-180e-4736-bbe9-3f7bfe02e188 | https://portal.gdc.cancer.gov/files/c144fb4d-180e-4736-bbe9-3f7bfe02e188 |
| c1566f1c-3b79-4803-8a62-23de06619811 | https://portal.gdc.cancer.gov/files/c1566f1c-3b79-4803-8a62-23de06619811 |
| c15f8331-b65f-4f7a-8d4d-d796dfcaf14d | https://portal.gdc.cancer.gov/files/c15f8331-b65f-4f7a-8d4d-d796dfcaf14d |
| c1b59cec-ca9f-4eff-acde-f1be4a0d623a | https://portal.gdc.cancer.gov/files/c1b59cec-ca9f-4eff-acde-f1be4a0d623a |
| c1b5df5c-b7fd-486b-8f49-1f748af9a079 | https://portal.gdc.cancer.gov/files/c1b5df5c-b7fd-486b-8f49-1f748af9a079 |
| c1ea600a-ac08-467b-b540-01b681ad8d9b | https://portal.gdc.cancer.gov/files/c1ea600a-ac08-467b-b540-01b681ad8d9b |
| c3dc14bf-1f6b-4284-b303-5caf8f121c03 | https://portal.gdc.cancer.gov/files/c3dc14bf-1f6b-4284-b303-5caf8f121c03 |
| c41fffbd-d5be-4b28-b0f4-2aaf7cb23555 | https://portal.gdc.cancer.gov/files/c41fffbd-d5be-4b28-b0f4-2aaf7cb23555 |
| c4e97f5b-9da4-4bb4-9ab5-9a9affec4e27 | https://portal.gdc.cancer.gov/files/c4e97f5b-9da4-4bb4-9ab5-9a9affec4e27 |
| c54bfdec-b24e-48b9-8fb0-c2d4e083d939 | https://portal.gdc.cancer.gov/files/c54bfdec-b24e-48b9-8fb0-c2d4e083d939 |
| c55ad7ab-27f1-49b5-844c-00daa90a9e55 | https://portal.gdc.cancer.gov/files/c55ad7ab-27f1-49b5-844c-00daa90a9e55 |
| c585dac6-771a-4663-a7d6-5c7d7ccae32a | https://portal.gdc.cancer.gov/files/c585dac6-771a-4663-a7d6-5c7d7ccae32a |
| c5a36c36-b9eb-4d09-94bd-9b7b6b0b992e | https://portal.gdc.cancer.gov/files/c5a36c36-b9eb-4d09-94bd-9b7b6b0b992e |
| c610d382-879b-43ae-8a0d-7ca2a27f990d | https://portal.gdc.cancer.gov/files/c610d382-879b-43ae-8a0d-7ca2a27f990d |
| c62d541d-71d6-461c-b316-6e4a4238d050 | https://portal.gdc.cancer.gov/files/c62d541d-71d6-461c-b316-6e4a4238d050 |
| c7405859-3e6d-4af2-8bdc-7f31d6441c35 | https://portal.gdc.cancer.gov/files/c7405859-3e6d-4af2-8bdc-7f31d6441c35 |
| c76da465-d5aa-4593-bd2e-ab983638ce7f | https://portal.gdc.cancer.gov/files/c76da465-d5aa-4593-bd2e-ab983638ce7f |
| c7a64911-e1b0-4615-9521-98d4cd4a9882 | https://portal.gdc.cancer.gov/files/c7a64911-e1b0-4615-9521-98d4cd4a9882 |
| c8a17ec0-e9fb-4edc-9904-de8b6871a066 | https://portal.gdc.cancer.gov/files/c8a17ec0-e9fb-4edc-9904-de8b6871a066 |
| c8c77aec-ba87-48bc-a56f-6e1ad2afe332 | https://portal.gdc.cancer.gov/files/c8c77aec-ba87-48bc-a56f-6e1ad2afe332 |
| ca9b8cf8-1db3-429b-b624-54762d25288c | https://portal.gdc.cancer.gov/files/ca9b8cf8-1db3-429b-b624-54762d25288c |
| caa31405-d6b1-467e-849a-b5c97f674e6a | https://portal.gdc.cancer.gov/files/caa31405-d6b1-467e-849a-b5c97f674e6a |
| cc0010d2-52db-4b15-a750-dd57d148520b | https://portal.gdc.cancer.gov/files/cc0010d2-52db-4b15-a750-dd57d148520b |
| cc1ab143-14a1-49f5-88d3-e386c8d6c0b1 | https://portal.gdc.cancer.gov/files/cc1ab143-14a1-49f5-88d3-e386c8d6c0b1 |
| cc46ac99-00d9-43e3-813c-ffc2f611ea32 | https://portal.gdc.cancer.gov/files/cc46ac99-00d9-43e3-813c-ffc2f611ea32 |
| cdca623f-7431-4d68-b54d-f2fd13ecfbf9 | https://portal.gdc.cancer.gov/files/cdca623f-7431-4d68-b54d-f2fd13ecfbf9 |
| ce1ff3ae-dcdd-4444-9a00-e74d018b833d | https://portal.gdc.cancer.gov/files/ce1ff3ae-dcdd-4444-9a00-e74d018b833d |
| d139256a-c9e8-417b-90af-c466a58d459d | https://portal.gdc.cancer.gov/files/d139256a-c9e8-417b-90af-c466a58d459d |
| d16e6c9d-cb8e-43d4-886a-6656b4cca09b | https://portal.gdc.cancer.gov/files/d16e6c9d-cb8e-43d4-886a-6656b4cca09b |
| d1a4787d-ee61-4667-95fb-5450536fa03c | https://portal.gdc.cancer.gov/files/d1a4787d-ee61-4667-95fb-5450536fa03c |
| d1b00628-da8a-4b57-a162-97129cf1ba73 | https://portal.gdc.cancer.gov/files/d1b00628-da8a-4b57-a162-97129cf1ba73 |
| d24185af-562f-4365-b9f9-49c6a0e06644 | https://portal.gdc.cancer.gov/files/d24185af-562f-4365-b9f9-49c6a0e06644 |
| d2fea0af-ffc7-45c7-a6cc-6cc7379df6fb | https://portal.gdc.cancer.gov/files/d2fea0af-ffc7-45c7-a6cc-6cc7379df6fb |
| d38093cf-9e44-4328-950a-37b81cf775ca | https://portal.gdc.cancer.gov/files/d38093cf-9e44-4328-950a-37b81cf775ca |
| d3fff383-01ff-4a06-9b7c-7f3542e5f24b | https://portal.gdc.cancer.gov/files/d3fff383-01ff-4a06-9b7c-7f3542e5f24b |
| d425acd5-869a-4c71-ba4a-4871f07d8b24 | https://portal.gdc.cancer.gov/files/d425acd5-869a-4c71-ba4a-4871f07d8b24 |
| d4426c56-9349-4770-b13c-17a2173015a0 | https://portal.gdc.cancer.gov/files/d4426c56-9349-4770-b13c-17a2173015a0 |
| d4aaf7f0-4944-46c7-a755-38976d004915 | https://portal.gdc.cancer.gov/files/d4aaf7f0-4944-46c7-a755-38976d004915 |
| d6919e3b-f539-4786-90f6-b93cd0da99b2 | https://portal.gdc.cancer.gov/files/d6919e3b-f539-4786-90f6-b93cd0da99b2 |
| d7c8e660-ab7f-4f68-9b88-5464f5670375 | https://portal.gdc.cancer.gov/files/d7c8e660-ab7f-4f68-9b88-5464f5670375 |
| d7e635d5-7330-491c-986c-f2737ec5d7bd | https://portal.gdc.cancer.gov/files/d7e635d5-7330-491c-986c-f2737ec5d7bd |
| d80985cc-be01-452c-931c-65fccbe783d9 | https://portal.gdc.cancer.gov/files/d80985cc-be01-452c-931c-65fccbe783d9 |
| d9364568-54c7-4c51-bbb1-75d4a58c39e7 | https://portal.gdc.cancer.gov/files/d9364568-54c7-4c51-bbb1-75d4a58c39e7 |
| da5a6e23-37a0-474b-8f6a-539b56f6ac74 | https://portal.gdc.cancer.gov/files/da5a6e23-37a0-474b-8f6a-539b56f6ac74 |
| db6f39e9-6134-47fe-92c7-35d71fd19e68 | https://portal.gdc.cancer.gov/files/db6f39e9-6134-47fe-92c7-35d71fd19e68 |
| dcffbcbe-e490-4bcf-95db-d454bf8df300 | https://portal.gdc.cancer.gov/files/dcffbcbe-e490-4bcf-95db-d454bf8df300 |
| ddc63fc0-30c0-4585-bf5d-97bee2b3a7a0 | https://portal.gdc.cancer.gov/files/ddc63fc0-30c0-4585-bf5d-97bee2b3a7a0 |
| ddeb287b-8d2f-40f2-89dc-def570237300 | https://portal.gdc.cancer.gov/files/ddeb287b-8d2f-40f2-89dc-def570237300 |
| df0c36cd-5eae-480f-871e-1aef2a418350 | https://portal.gdc.cancer.gov/files/df0c36cd-5eae-480f-871e-1aef2a418350 |
| df6ab599-42be-4cb8-a35c-34bdcd4809f7 | https://portal.gdc.cancer.gov/files/df6ab599-42be-4cb8-a35c-34bdcd4809f7 |
| df736f4c-b9d6-4805-bd7d-7bc6d92174d7 | https://portal.gdc.cancer.gov/files/df736f4c-b9d6-4805-bd7d-7bc6d92174d7 |
| e0b785ae-608a-43a9-805e-8adfbda809a4 | https://portal.gdc.cancer.gov/files/e0b785ae-608a-43a9-805e-8adfbda809a4 |
| e106f526-1eda-40aa-80e8-ac417b0a8fb5 | https://portal.gdc.cancer.gov/files/e106f526-1eda-40aa-80e8-ac417b0a8fb5 |
| e1677731-3f36-462c-8dc1-75e98a4df270 | https://portal.gdc.cancer.gov/files/e1677731-3f36-462c-8dc1-75e98a4df270 |
| e1ab994c-401a-4b92-ae85-59a421b89b52 | https://portal.gdc.cancer.gov/files/e1ab994c-401a-4b92-ae85-59a421b89b52 |
| e1f2a612-49d9-4935-9791-cfd9abbf7f92 | https://portal.gdc.cancer.gov/files/e1f2a612-49d9-4935-9791-cfd9abbf7f92 |
| e20b808b-81cd-41ce-98eb-e2dc3db7fece | https://portal.gdc.cancer.gov/files/e20b808b-81cd-41ce-98eb-e2dc3db7fece |
| e3657009-4f9e-4632-a458-2bf17ec93fbd | https://portal.gdc.cancer.gov/files/e3657009-4f9e-4632-a458-2bf17ec93fbd |
| e5c65ba0-d937-4ed9-aa81-99bc48717a2d | https://portal.gdc.cancer.gov/files/e5c65ba0-d937-4ed9-aa81-99bc48717a2d |
| e5d09324-3744-41d8-9339-ede59d27086d | https://portal.gdc.cancer.gov/files/e5d09324-3744-41d8-9339-ede59d27086d |
| e5f2fe00-70ca-4fc8-bfd4-d34bfee2e11a | https://portal.gdc.cancer.gov/files/e5f2fe00-70ca-4fc8-bfd4-d34bfee2e11a |
| e67eb79f-6860-49ca-8cf2-bda65255fb98 | https://portal.gdc.cancer.gov/files/e67eb79f-6860-49ca-8cf2-bda65255fb98 |
| e6acd0ab-16ba-468e-b356-5e9f0ba9be67 | https://portal.gdc.cancer.gov/files/e6acd0ab-16ba-468e-b356-5e9f0ba9be67 |
| e7e3047d-15bc-4dcc-aaca-86e5e2f45441 | https://portal.gdc.cancer.gov/files/e7e3047d-15bc-4dcc-aaca-86e5e2f45441 |
| e820ebb7-91de-45f6-b03d-f97fca659059 | https://portal.gdc.cancer.gov/files/e820ebb7-91de-45f6-b03d-f97fca659059 |
| e8627623-e4a5-46a1-8914-6b41bd84635d | https://portal.gdc.cancer.gov/files/e8627623-e4a5-46a1-8914-6b41bd84635d |
| e86ffb5d-3328-48f1-8311-bfba3d21c85d | https://portal.gdc.cancer.gov/files/e86ffb5d-3328-48f1-8311-bfba3d21c85d |
| e9320bf6-7e13-4cdb-bde3-a64c581622d4 | https://portal.gdc.cancer.gov/files/e9320bf6-7e13-4cdb-bde3-a64c581622d4 |
| e9a08dd9-4a87-44d5-bc76-eb714ac62d09 | https://portal.gdc.cancer.gov/files/e9a08dd9-4a87-44d5-bc76-eb714ac62d09 |
| e9c3fdc0-ea70-491c-8370-c86f37ff815f | https://portal.gdc.cancer.gov/files/e9c3fdc0-ea70-491c-8370-c86f37ff815f |
| e9d20052-96ee-4b64-b61b-cbdcbd341446 | https://portal.gdc.cancer.gov/files/e9d20052-96ee-4b64-b61b-cbdcbd341446 |
| eb65b8e1-81e1-4d4e-bddc-fa9b005fcc12 | https://portal.gdc.cancer.gov/files/eb65b8e1-81e1-4d4e-bddc-fa9b005fcc12 |
| eb9d8b0e-a024-43b2-943f-3aca44561e1d | https://portal.gdc.cancer.gov/files/eb9d8b0e-a024-43b2-943f-3aca44561e1d |
| ebb6d650-3376-438d-a66c-9b866551be5a | https://portal.gdc.cancer.gov/files/ebb6d650-3376-438d-a66c-9b866551be5a |
| ebd7bdc4-c7c0-46c4-835b-8afc2a6c2615 | https://portal.gdc.cancer.gov/files/ebd7bdc4-c7c0-46c4-835b-8afc2a6c2615 |
| ebd9a9d0-c711-42e3-a382-9ef1fb5b7184 | https://portal.gdc.cancer.gov/files/ebd9a9d0-c711-42e3-a382-9ef1fb5b7184 |
| ec255ed5-156b-4e5b-bbb7-d7841395fefe | https://portal.gdc.cancer.gov/files/ec255ed5-156b-4e5b-bbb7-d7841395fefe |
| ed8eb2e9-ef8f-4b72-9c01-3504a578b500 | https://portal.gdc.cancer.gov/files/ed8eb2e9-ef8f-4b72-9c01-3504a578b500 |
| f031e82a-99ac-46fc-abeb-7ad9bd535812 | https://portal.gdc.cancer.gov/files/f031e82a-99ac-46fc-abeb-7ad9bd535812 |
| f12570e9-0af7-4882-8cd5-33171e3537b0 | https://portal.gdc.cancer.gov/files/f12570e9-0af7-4882-8cd5-33171e3537b0 |
| f154e063-d06b-43a9-9447-0aa933b420ba | https://portal.gdc.cancer.gov/files/f154e063-d06b-43a9-9447-0aa933b420ba |
| f2f970cd-cf1c-43e3-8817-3a5aecab8ae3 | https://portal.gdc.cancer.gov/files/f2f970cd-cf1c-43e3-8817-3a5aecab8ae3 |
| f3ad08a9-3fa8-43a1-b7a1-82d548ad1475 | https://portal.gdc.cancer.gov/files/f3ad08a9-3fa8-43a1-b7a1-82d548ad1475 |
| f3cc686f-c4e3-4d83-839c-dcb2f71ab9f7 | https://portal.gdc.cancer.gov/files/f3cc686f-c4e3-4d83-839c-dcb2f71ab9f7 |
| f5f24dbe-e9a3-4a88-8286-dfeb8bc10d87 | https://portal.gdc.cancer.gov/files/f5f24dbe-e9a3-4a88-8286-dfeb8bc10d87 |
| f609a9c2-1c53-4bbb-9b40-ce736514e7de | https://portal.gdc.cancer.gov/files/f609a9c2-1c53-4bbb-9b40-ce736514e7de |
| f6c89e33-7ba1-43f3-89b2-927ea8162978 | https://portal.gdc.cancer.gov/files/f6c89e33-7ba1-43f3-89b2-927ea8162978 |
| f7297699-af69-426e-9087-c3112a92d85c | https://portal.gdc.cancer.gov/files/f7297699-af69-426e-9087-c3112a92d85c |
| f8a5ba3d-cecf-45fd-8b92-14ca47945d1c | https://portal.gdc.cancer.gov/files/f8a5ba3d-cecf-45fd-8b92-14ca47945d1c |
| f930f74f-0f88-4289-8bda-7ebd1dc4ea67 | https://portal.gdc.cancer.gov/files/f930f74f-0f88-4289-8bda-7ebd1dc4ea67 |
| f9903ebc-4434-40d9-ad03-20ca825a666e | https://portal.gdc.cancer.gov/files/f9903ebc-4434-40d9-ad03-20ca825a666e |
| fa515a26-a43e-4b6e-8120-07a9c3b6352e | https://portal.gdc.cancer.gov/files/fa515a26-a43e-4b6e-8120-07a9c3b6352e |
| fa95f8cd-d608-430e-a8b3-68a05a5051e2 | https://portal.gdc.cancer.gov/files/fa95f8cd-d608-430e-a8b3-68a05a5051e2 |
| fad707d3-c11c-4aec-a5d8-53c4622011d1 | https://portal.gdc.cancer.gov/files/fad707d3-c11c-4aec-a5d8-53c4622011d1 |
| fb310e6b-1b46-4a67-8a1a-16d9be837dc6 | https://portal.gdc.cancer.gov/files/fb310e6b-1b46-4a67-8a1a-16d9be837dc6 |
| fcf07cf2-437a-4178-a405-c34d800cf75f | https://portal.gdc.cancer.gov/files/fcf07cf2-437a-4178-a405-c34d800cf75f |
| fcff3742-b3b9-4860-931f-819d85164bc7 | https://portal.gdc.cancer.gov/files/fcff3742-b3b9-4860-931f-819d85164bc7 |
| fd08f2ae-5b8a-45cf-a635-6101abb1e287 | https://portal.gdc.cancer.gov/files/fd08f2ae-5b8a-45cf-a635-6101abb1e287 |
| fd229d04-f96d-4e4f-bef7-24bbb9e1fffc | https://portal.gdc.cancer.gov/files/fd229d04-f96d-4e4f-bef7-24bbb9e1fffc |
| fd50b935-b9e1-4e0d-bedb-a357adc2b57e | https://portal.gdc.cancer.gov/files/fd50b935-b9e1-4e0d-bedb-a357adc2b57e |
| fe9e4c81-7149-4cfd-b1a2-60884268988b | https://portal.gdc.cancer.gov/files/fe9e4c81-7149-4cfd-b1a2-60884268988b |
| feb06cd1-e6f5-4e39-9b19-a6479f751c38 | https://portal.gdc.cancer.gov/files/feb06cd1-e6f5-4e39-9b19-a6479f751c38 |
| ff12abd3-0f45-4063-afa7-fa5cad973159 | https://portal.gdc.cancer.gov/files/ff12abd3-0f45-4063-afa7-fa5cad973159 |
| ff54fafe-1168-4921-af2c-2be600c9430e | https://portal.gdc.cancer.gov/files/ff54fafe-1168-4921-af2c-2be600c9430e |
