# Supplementary material for: Prognostic prediction and immune infiltration analysis based on ferroptosis and EMT state in hepatocellular carcinoma
Source: Front Immunol. 2022 Dec 15;13:1076045. doi: 10.3389/fimmu.2022.1076045 (PMC9797854; doi:10.3389/fimmu.2022.1076045)
Supplement: Supplementary file 4 [file Table_3.docx]

**Supplementary table 3** The clinical data files (n=377) of HCC patients from the TCGA website.

| id | links |
| --- | --- |
| 004d6594-95ce-494c-9760-828f9885bbae | <https://portal.gdc.cancer.gov/files/004d6594-95ce-494c-9760-828f9885bbae> |
| 00a9a7f4-06eb-40fb-8d3a-66f5f5d315f7 | <https://portal.gdc.cancer.gov/files/00a9a7f4-06eb-40fb-8d3a-66f5f5d315f7> |
| 010f9d46-fd87-4b69-9eb9-b5b6fd34cf42 | <https://portal.gdc.cancer.gov/files/010f9d46-fd87-4b69-9eb9-b5b6fd34cf42> |
| 015e736d-7097-49d6-9a0b-eb78b932da11 | https://portal.gdc.cancer.gov/files/015e736d-7097-49d6-9a0b-eb78b932da11 |
| 040002dd-7797-48bd-9e91-1d75d8eb756a | https://portal.gdc.cancer.gov/files/040002dd-7797-48bd-9e91-1d75d8eb756a |
| 045cbaae-5088-4dab-84b2-4cf4fae94a3d | https://portal.gdc.cancer.gov/files/045cbaae-5088-4dab-84b2-4cf4fae94a3d |
| 04f27de4-ab30-430e-b8c6-20d1e83da4e9 | https://portal.gdc.cancer.gov/files/04f27de4-ab30-430e-b8c6-20d1e83da4e9 |
| 0659fc68-a533-4d96-84ba-1aaa4002cd2e | https://portal.gdc.cancer.gov/files/0659fc68-a533-4d96-84ba-1aaa4002cd2e |
| 08131208-2f43-4b7c-a891-9acbd7eaa353 | https://portal.gdc.cancer.gov/files/08131208-2f43-4b7c-a891-9acbd7eaa353 |
| 092c7219-c33c-4a5b-9c4a-33067000d40a | https://portal.gdc.cancer.gov/files/092c7219-c33c-4a5b-9c4a-33067000d40a |
| 09b41222-958c-4e50-8e52-fc989d2916f3 | https://portal.gdc.cancer.gov/files/09b41222-958c-4e50-8e52-fc989d2916f3 |
| 0ad5f933-2032-4be5-8813-ab087e06fac6 | https://portal.gdc.cancer.gov/files/0ad5f933-2032-4be5-8813-ab087e06fac6 |
| 0ba6354e-0fce-42c5-afdd-1ea20644283e | https://portal.gdc.cancer.gov/files/0ba6354e-0fce-42c5-afdd-1ea20644283e |
| 0e7d3a4c-1bc0-42a6-9617-54e14718bff7 | https://portal.gdc.cancer.gov/files/0e7d3a4c-1bc0-42a6-9617-54e14718bff7 |
| 0f278d5f-5bec-42f7-ab55-92117ec2a2bc | https://portal.gdc.cancer.gov/files/0f278d5f-5bec-42f7-ab55-92117ec2a2bc |
| 1183f22b-fcc3-48e9-a2b3-be84b73b1b99 | https://portal.gdc.cancer.gov/files/1183f22b-fcc3-48e9-a2b3-be84b73b1b99 |
| 11d5b694-caf7-41b0-bc50-ee410ce6791c | https://portal.gdc.cancer.gov/files/11d5b694-caf7-41b0-bc50-ee410ce6791c |
| 12096b18-ff08-4366-b2b7-fca0eabea733 | https://portal.gdc.cancer.gov/files/12096b18-ff08-4366-b2b7-fca0eabea733 |
| 13a518cf-a57e-4018-8fd5-70fa5fc4a29f | https://portal.gdc.cancer.gov/files/13a518cf-a57e-4018-8fd5-70fa5fc4a29f |
| 13d65949-24a5-46c1-817e-8845394f8e0e | <https://portal.gdc.cancer.gov/files/13d65949-24a5-46c1-817e-8845394f8e0e> |
| 14b085bb-444f-48ac-b205-85bd34c4f20a | https://portal.gdc.cancer.gov/files/14b085bb-444f-48ac-b205-85bd34c4f20a |
| 14bf3e0d-05c4-4f6f-9af8-96374c942d7a | https://portal.gdc.cancer.gov/files/14bf3e0d-05c4-4f6f-9af8-96374c942d7a |
| 157e77a9-910b-47fa-a9ec-6d1ac9c48f9d | https://portal.gdc.cancer.gov/files/157e77a9-910b-47fa-a9ec-6d1ac9c48f9d |
| 15a30f2e-0799-498e-8d9d-ca37dec7b2aa | https://portal.gdc.cancer.gov/files/15a30f2e-0799-498e-8d9d-ca37dec7b2aa |
| 169ace81-27a2-48eb-a5fc-f32f4ca9d5b8 | https://portal.gdc.cancer.gov/files/169ace81-27a2-48eb-a5fc-f32f4ca9d5b8 |
| 16e5cb18-f2ae-4a18-8936-38ab8cf0ff3d | https://portal.gdc.cancer.gov/files/16e5cb18-f2ae-4a18-8936-38ab8cf0ff3d |
| 17880fee-3e71-4d8e-93bd-5f6b1466fcb3 | https://portal.gdc.cancer.gov/files/17880fee-3e71-4d8e-93bd-5f6b1466fcb3 |
| 18bb9b2f-8804-4686-b585-901f95b7bd33 | https://portal.gdc.cancer.gov/files/18bb9b2f-8804-4686-b585-901f95b7bd33 |
| 18de3f55-a2b3-495a-a802-a4f6705242cc | https://portal.gdc.cancer.gov/files/18de3f55-a2b3-495a-a802-a4f6705242cc |
| 18e9e0bb-849d-4aed-982c-8b3b978cdfe3 | https://portal.gdc.cancer.gov/files/18e9e0bb-849d-4aed-982c-8b3b978cdfe3 |
| 199b2c04-8ac7-459a-8913-23c91e955a54 | https://portal.gdc.cancer.gov/files/199b2c04-8ac7-459a-8913-23c91e955a54 |
| 19d2dcd0-7778-4f39-9955-83fc374d6349 | https://portal.gdc.cancer.gov/files/19d2dcd0-7778-4f39-9955-83fc374d6349 |
| 1ac66576-1b8d-4d07-a716-4f6717b77b54 | https://portal.gdc.cancer.gov/files/1ac66576-1b8d-4d07-a716-4f6717b77b54 |
| 1b6f262a-6c6f-47b8-8994-7eae924c00bd | https://portal.gdc.cancer.gov/files/1b6f262a-6c6f-47b8-8994-7eae924c00bd |
| 1bc780db-505b-4ef0-aea5-d768b2ffc151 | https://portal.gdc.cancer.gov/files/1bc780db-505b-4ef0-aea5-d768b2ffc151 |
| 1c92315c-b055-4b3c-81cb-0c7de194682b | https://portal.gdc.cancer.gov/files/1c92315c-b055-4b3c-81cb-0c7de194682b |
| 1cc2e93c-cd14-485e-b031-bd296a851226 | https://portal.gdc.cancer.gov/files/1cc2e93c-cd14-485e-b031-bd296a851226 |
| 1d85713a-1995-4763-a2ae-d8978605ccf4 | https://portal.gdc.cancer.gov/files/1d85713a-1995-4763-a2ae-d8978605ccf4 |
| 1f58ca10-bf1d-4ad9-b15d-453572228e8f | https://portal.gdc.cancer.gov/files/1f58ca10-bf1d-4ad9-b15d-453572228e8f |
| 1fe27356-27b8-420f-acea-e9798a1bfa81 | https://portal.gdc.cancer.gov/files/1fe27356-27b8-420f-acea-e9798a1bfa81 |
| 2036c158-f105-46ff-98c4-b0cf9a1baeda | https://portal.gdc.cancer.gov/files/2036c158-f105-46ff-98c4-b0cf9a1baeda |
| 2036c620-c00e-4939-877c-1f86294a5226 | https://portal.gdc.cancer.gov/files/2036c620-c00e-4939-877c-1f86294a5226 |
| 20c2e09b-a887-4044-9e32-8e732910031b | https://portal.gdc.cancer.gov/files/20c2e09b-a887-4044-9e32-8e732910031b |
| 2178dc75-09b9-40ef-ad5c-097048f50fee | https://portal.gdc.cancer.gov/files/2178dc75-09b9-40ef-ad5c-097048f50fee |
| 21a48c0b-fee9-45ce-8eff-353204674914 | https://portal.gdc.cancer.gov/files/21a48c0b-fee9-45ce-8eff-353204674914 |
| 2265433f-de7f-4829-aae5-7cbf5aa9f093 | https://portal.gdc.cancer.gov/files/2265433f-de7f-4829-aae5-7cbf5aa9f093 |
| 23b2ab2e-a211-48ab-95a5-e2c360418cc8 | https://portal.gdc.cancer.gov/files/23b2ab2e-a211-48ab-95a5-e2c360418cc8 |
| 23e7252a-9a4f-480b-bf22-f2bf92154094 | https://portal.gdc.cancer.gov/files/23e7252a-9a4f-480b-bf22-f2bf92154094 |
| 254198a9-bfe4-4523-874b-94bf4d96a361 | https://portal.gdc.cancer.gov/files/254198a9-bfe4-4523-874b-94bf4d96a361 |
| 27436f0c-2c49-48ce-9b6a-d3077d89bea3 | https://portal.gdc.cancer.gov/files/27436f0c-2c49-48ce-9b6a-d3077d89bea3 |
| 2851017f-325e-4004-8c7e-fb7cc5589856 | https://portal.gdc.cancer.gov/files/2851017f-325e-4004-8c7e-fb7cc5589856 |
| 28537c5c-2a18-420c-b992-897ae5336de7 | https://portal.gdc.cancer.gov/files/28537c5c-2a18-420c-b992-897ae5336de7 |
| 297557e9-fea4-4367-a16a-00d12ac49de9 | https://portal.gdc.cancer.gov/files/297557e9-fea4-4367-a16a-00d12ac49de9 |
| 2a2815b5-d110-43f0-8cb5-45099ca4a8bb | https://portal.gdc.cancer.gov/files/2a2815b5-d110-43f0-8cb5-45099ca4a8bb |
| 2a471698-f4eb-4c4b-8a31-46d4134a8d16 | https://portal.gdc.cancer.gov/files/2a471698-f4eb-4c4b-8a31-46d4134a8d16 |
| 2e08a16b-30e3-4e86-835b-933a83e11d4a | https://portal.gdc.cancer.gov/files/2e08a16b-30e3-4e86-835b-933a83e11d4a |
| 2e9c92cc-888a-4c76-b373-dc60583343e0 | https://portal.gdc.cancer.gov/files/2e9c92cc-888a-4c76-b373-dc60583343e0 |
| 2f25fca6-347e-4b34-a36a-0ae55744f4b8 | https://portal.gdc.cancer.gov/files/2f25fca6-347e-4b34-a36a-0ae55744f4b8 |
| 2f42421c-d6a2-450a-8379-7abf1f3bb3d1 | https://portal.gdc.cancer.gov/files/2f42421c-d6a2-450a-8379-7abf1f3bb3d1 |
| 2f58bad5-b366-4def-acac-e243f24da6ab | https://portal.gdc.cancer.gov/files/2f58bad5-b366-4def-acac-e243f24da6ab |
| 31c8971d-123e-4acd-8732-183ef494f703 | https://portal.gdc.cancer.gov/files/31c8971d-123e-4acd-8732-183ef494f703 |
| 31f6aba1-31ed-43d4-bcca-2c2a1a2a90fc | https://portal.gdc.cancer.gov/files/31f6aba1-31ed-43d4-bcca-2c2a1a2a90fc |
| 31f856d3-afa7-4732-8a7e-393d73f70c2c | https://portal.gdc.cancer.gov/files/31f856d3-afa7-4732-8a7e-393d73f70c2c |
| 32506165-a07f-45a6-a1b5-30a3dac7f5ee | https://portal.gdc.cancer.gov/files/32506165-a07f-45a6-a1b5-30a3dac7f5ee |
| 32687e5b-f160-43c0-abcd-ac8c1b2ea220 | https://portal.gdc.cancer.gov/files/32687e5b-f160-43c0-abcd-ac8c1b2ea220 |
| 348e5988-2410-43bd-b3a4-6f85675da574 | https://portal.gdc.cancer.gov/files/348e5988-2410-43bd-b3a4-6f85675da574 |
| 3567d75b-6132-4140-9e50-9ce48c84f4d8 | https://portal.gdc.cancer.gov/files/3567d75b-6132-4140-9e50-9ce48c84f4d8 |
| 35a9e905-7790-4cc4-bd96-b546e13d4f89 | https://portal.gdc.cancer.gov/files/35a9e905-7790-4cc4-bd96-b546e13d4f89 |
| 361716fc-9b08-4670-8928-e54d27abd5cb | https://portal.gdc.cancer.gov/files/361716fc-9b08-4670-8928-e54d27abd5cb |
| 36301b4b-c072-4fc8-9e3d-87c5ced8e3c8 | https://portal.gdc.cancer.gov/files/36301b4b-c072-4fc8-9e3d-87c5ced8e3c8 |
| 3666580a-e7d9-48fe-be92-8b43b8dc012d | https://portal.gdc.cancer.gov/files/3666580a-e7d9-48fe-be92-8b43b8dc012d |
| 36fbb49c-d0f1-43d2-86ea-084d2f79300c | https://portal.gdc.cancer.gov/files/36fbb49c-d0f1-43d2-86ea-084d2f79300c |
| 3733bd53-5442-44be-923f-87351c6bbe9f | https://portal.gdc.cancer.gov/files/3733bd53-5442-44be-923f-87351c6bbe9f |
| 38257927-d9c5-4d10-a774-61684bad59a4 | https://portal.gdc.cancer.gov/files/38257927-d9c5-4d10-a774-61684bad59a4 |
| 3833c121-4683-47f5-a948-714774de5a44 | https://portal.gdc.cancer.gov/files/3833c121-4683-47f5-a948-714774de5a44 |
| 3b0704d2-3405-4256-917f-fd0ae05c7065 | https://portal.gdc.cancer.gov/files/3b0704d2-3405-4256-917f-fd0ae05c7065 |
| 3b0e3a11-33c9-4616-b76c-4cb1406de806 | https://portal.gdc.cancer.gov/files/3b0e3a11-33c9-4616-b76c-4cb1406de806 |
| 3baa8c85-abe5-4c98-bb00-0f966335cd5a | https://portal.gdc.cancer.gov/files/3baa8c85-abe5-4c98-bb00-0f966335cd5a |
| 3bc8d577-fa61-455f-9a26-f4037635ce89 | https://portal.gdc.cancer.gov/files/3bc8d577-fa61-455f-9a26-f4037635ce89 |
| 3c2d478b-8294-4c98-aedc-2789864a42b0 | https://portal.gdc.cancer.gov/files/3c2d478b-8294-4c98-aedc-2789864a42b0 |
| 3c6ee8cb-df36-4e1b-8346-17b01bf54aa0 | https://portal.gdc.cancer.gov/files/3c6ee8cb-df36-4e1b-8346-17b01bf54aa0 |
| 3cd7b0cf-2c29-4355-9216-503756a2977d | https://portal.gdc.cancer.gov/files/3cd7b0cf-2c29-4355-9216-503756a2977d |
| 3d6628d1-c990-4657-81a5-2e9d93cfaeff | https://portal.gdc.cancer.gov/files/3d6628d1-c990-4657-81a5-2e9d93cfaeff |
| 4030a2b2-4026-4d11-a1f3-c8c89cab6892 | https://portal.gdc.cancer.gov/files/4030a2b2-4026-4d11-a1f3-c8c89cab6892 |
| 4068c318-e969-4e12-9856-4cabb00dadd9 | https://portal.gdc.cancer.gov/files/4068c318-e969-4e12-9856-4cabb00dadd9 |
| 40b19bf1-239d-4c33-9dd7-e22887ca07f3 | https://portal.gdc.cancer.gov/files/40b19bf1-239d-4c33-9dd7-e22887ca07f3 |
| 40ba6512-cf6a-41df-8672-04f5e2f1f630 | https://portal.gdc.cancer.gov/files/40ba6512-cf6a-41df-8672-04f5e2f1f630 |
| 43361fd0-8d46-43da-b0d5-9b81e0dbb5f2 | https://portal.gdc.cancer.gov/files/43361fd0-8d46-43da-b0d5-9b81e0dbb5f2 |
| 434c5fda-e58d-4fc9-a3d0-d8d39d429cdc | https://portal.gdc.cancer.gov/files/434c5fda-e58d-4fc9-a3d0-d8d39d429cdc |
| 44504ef2-154d-43dd-b20a-d0daf25d18ab | https://portal.gdc.cancer.gov/files/44504ef2-154d-43dd-b20a-d0daf25d18ab |
| 454565d0-36eb-4e14-a671-fa98845ce0c1 | https://portal.gdc.cancer.gov/files/454565d0-36eb-4e14-a671-fa98845ce0c1 |
| 45cb599a-11b3-4a84-afd6-004ed01aab3c | https://portal.gdc.cancer.gov/files/45cb599a-11b3-4a84-afd6-004ed01aab3c |
| 47506bea-df46-4d96-882a-664a6e3277fe | https://portal.gdc.cancer.gov/files/47506bea-df46-4d96-882a-664a6e3277fe |
| 48160afb-cca2-4025-8075-1e0ee735f5f2 | https://portal.gdc.cancer.gov/files/48160afb-cca2-4025-8075-1e0ee735f5f2 |
| 4865d2a2-b066-4537-8faf-fd1ad5290c01 | https://portal.gdc.cancer.gov/files/4865d2a2-b066-4537-8faf-fd1ad5290c01 |
| 4a238239-3f17-4cd1-889e-41a86ddd36ac | https://portal.gdc.cancer.gov/files/4a238239-3f17-4cd1-889e-41a86ddd36ac |
| 4bf01e41-5422-41c0-af81-6f88f939f341 | https://portal.gdc.cancer.gov/files/4bf01e41-5422-41c0-af81-6f88f939f341 |
| 4e3d47ea-94df-444c-95ec-4c806ae0e8fb | https://portal.gdc.cancer.gov/files/4e3d47ea-94df-444c-95ec-4c806ae0e8fb |
| 4eafc9fc-cd23-469f-a6db-9e7afb38e6ea | https://portal.gdc.cancer.gov/files/4eafc9fc-cd23-469f-a6db-9e7afb38e6ea |
| 4f24ca90-84f2-4b4b-a684-f4d2bfd1c5fb | https://portal.gdc.cancer.gov/files/4f24ca90-84f2-4b4b-a684-f4d2bfd1c5fb |
| 4f8064d0-39a7-4af3-9f45-f3002a18fbcf | https://portal.gdc.cancer.gov/files/4f8064d0-39a7-4af3-9f45-f3002a18fbcf |
| 513110fa-5de6-4ff6-acf4-2c7e0252b6c6 | https://portal.gdc.cancer.gov/files/513110fa-5de6-4ff6-acf4-2c7e0252b6c6 |
| 51f288b8-2b73-4281-a750-511c55e2f88b | https://portal.gdc.cancer.gov/files/51f288b8-2b73-4281-a750-511c55e2f88b |
| 52360311-c7a6-4e29-8941-2ceaf1469233 | https://portal.gdc.cancer.gov/files/52360311-c7a6-4e29-8941-2ceaf1469233 |
| 528ff77d-0b54-4813-9fa0-b9b268b2e4ec | https://portal.gdc.cancer.gov/files/528ff77d-0b54-4813-9fa0-b9b268b2e4ec |
| 5292a521-8b15-41fb-9149-5e40e4f8d9e8 | https://portal.gdc.cancer.gov/files/5292a521-8b15-41fb-9149-5e40e4f8d9e8 |
| 53a6e0e5-90ab-4f7e-9ba1-7eab8feca98e | https://portal.gdc.cancer.gov/files/53a6e0e5-90ab-4f7e-9ba1-7eab8feca98e |
| 53ab51c5-a2f2-4ffe-877b-9c9ae282e8ac | https://portal.gdc.cancer.gov/files/53ab51c5-a2f2-4ffe-877b-9c9ae282e8ac |
| 54e01386-e773-4f20-b4e9-7ff9d902441d | https://portal.gdc.cancer.gov/files/54e01386-e773-4f20-b4e9-7ff9d902441d |
| 54ef72fc-bc0a-41ea-8bc4-636b380370db | https://portal.gdc.cancer.gov/files/54ef72fc-bc0a-41ea-8bc4-636b380370db |
| 551c071b-b794-4a55-af98-4c5191fcce00 | https://portal.gdc.cancer.gov/files/551c071b-b794-4a55-af98-4c5191fcce00 |
| 552bd81b-be8a-4b3b-ae0f-0b09668d0ab1 | https://portal.gdc.cancer.gov/files/552bd81b-be8a-4b3b-ae0f-0b09668d0ab1 |
| 554e07f8-de0e-4910-be55-4c705dc9b6c2 | https://portal.gdc.cancer.gov/files/554e07f8-de0e-4910-be55-4c705dc9b6c2 |
| 557ea5ef-3206-4ab6-b447-a2b39446ae48 | https://portal.gdc.cancer.gov/files/557ea5ef-3206-4ab6-b447-a2b39446ae48 |
| 55ffe94d-3e78-4816-a123-118731311824 | https://portal.gdc.cancer.gov/files/55ffe94d-3e78-4816-a123-118731311824 |
| 56097a08-776a-43b7-b4ce-0c6f94868ee7 | https://portal.gdc.cancer.gov/files/56097a08-776a-43b7-b4ce-0c6f94868ee7 |
| 56902d42-c4fe-4070-944b-a2acac99bb7f | https://portal.gdc.cancer.gov/files/56902d42-c4fe-4070-944b-a2acac99bb7f |
| 58faa8f8-0d2f-4de6-8856-d5543dda6eea | https://portal.gdc.cancer.gov/files/58faa8f8-0d2f-4de6-8856-d5543dda6eea |
| 594e5659-5850-4043-9090-ae5a0deafc89 | https://portal.gdc.cancer.gov/files/594e5659-5850-4043-9090-ae5a0deafc89 |
| 59c6c181-6fff-4da4-b61d-82e5c91d939c | https://portal.gdc.cancer.gov/files/59c6c181-6fff-4da4-b61d-82e5c91d939c |
| 5a2bf6a3-7420-43d0-9c90-da99168ee402 | https://portal.gdc.cancer.gov/files/5a2bf6a3-7420-43d0-9c90-da99168ee402 |
| 5a64da83-dfd6-4cd5-8526-dbd5f28c5629 | https://portal.gdc.cancer.gov/files/5a64da83-dfd6-4cd5-8526-dbd5f28c5629 |
| 5a8e2f5b-0160-4f04-8ccc-8ca96968a7f6 | https://portal.gdc.cancer.gov/files/5a8e2f5b-0160-4f04-8ccc-8ca96968a7f6 |
| 5c132009-890d-4c66-a3c0-0f8a85261b77 | https://portal.gdc.cancer.gov/files/5c132009-890d-4c66-a3c0-0f8a85261b77 |
| 5e16d7ae-05b9-4469-9710-bce32121cc29 | https://portal.gdc.cancer.gov/files/5e16d7ae-05b9-4469-9710-bce32121cc29 |
| 5f164953-8b78-4ce0-a1d9-be97ad7d5186 | https://portal.gdc.cancer.gov/files/5f164953-8b78-4ce0-a1d9-be97ad7d5186 |
| 6103bdba-e596-49db-bbda-e585ca49a5d2 | https://portal.gdc.cancer.gov/files/6103bdba-e596-49db-bbda-e585ca49a5d2 |
| 613c082d-6daf-4d70-b2bd-6910412e1108 | https://portal.gdc.cancer.gov/files/613c082d-6daf-4d70-b2bd-6910412e1108 |
| 61769966-bcd0-428a-abf8-c39f3103141a | https://portal.gdc.cancer.gov/files/61769966-bcd0-428a-abf8-c39f3103141a |
| 6193be0c-43cf-4629-9ed5-ebab7b1f1a6a | https://portal.gdc.cancer.gov/files/6193be0c-43cf-4629-9ed5-ebab7b1f1a6a |
| 61d7ab82-8c7f-4b47-9ebd-ee202256f446 | https://portal.gdc.cancer.gov/files/61d7ab82-8c7f-4b47-9ebd-ee202256f446 |
| 62407d77-6b0b-4231-af58-06ef6b7d363b | https://portal.gdc.cancer.gov/files/62407d77-6b0b-4231-af58-06ef6b7d363b |
| 628b95d6-c9cd-4613-a3e8-92e9ecc14a6f | https://portal.gdc.cancer.gov/files/628b95d6-c9cd-4613-a3e8-92e9ecc14a6f |
| 631ce446-b882-4f64-9784-3a9be6f5ef74 | https://portal.gdc.cancer.gov/files/631ce446-b882-4f64-9784-3a9be6f5ef74 |
| 6321e188-9167-448b-aad7-c5e366172016 | https://portal.gdc.cancer.gov/files/6321e188-9167-448b-aad7-c5e366172016 |
| 6330af27-8341-463a-ae45-d471a19af5dc | https://portal.gdc.cancer.gov/files/6330af27-8341-463a-ae45-d471a19af5dc |
| 645bfa68-25fd-406c-af4c-0ddcca92e744 | https://portal.gdc.cancer.gov/files/645bfa68-25fd-406c-af4c-0ddcca92e744 |
| 6473cb58-44b3-497c-b8e4-79218dccb657 | https://portal.gdc.cancer.gov/files/6473cb58-44b3-497c-b8e4-79218dccb657 |
| 65c4e2c7-8418-48c6-ba10-a044582d33ed | https://portal.gdc.cancer.gov/files/65c4e2c7-8418-48c6-ba10-a044582d33ed |
| 65de7782-2cf6-4a58-bc10-3e0b5eb11923 | https://portal.gdc.cancer.gov/files/65de7782-2cf6-4a58-bc10-3e0b5eb11923 |
| 6674c2c3-a2a7-4231-b9b4-59d475303eaa | https://portal.gdc.cancer.gov/files/6674c2c3-a2a7-4231-b9b4-59d475303eaa |
| 672ee1c9-fa75-42a5-912f-200de1536296 | https://portal.gdc.cancer.gov/files/672ee1c9-fa75-42a5-912f-200de1536296 |
| 673fdc08-3cc0-41f3-b929-fd6891023374 | https://portal.gdc.cancer.gov/files/673fdc08-3cc0-41f3-b929-fd6891023374 |
| 67b71118-136c-407d-a572-daf7ef345fc9 | https://portal.gdc.cancer.gov/files/67b71118-136c-407d-a572-daf7ef345fc9 |
| 67f88e42-216c-4387-960a-3256f5a1c3fd | https://portal.gdc.cancer.gov/files/67f88e42-216c-4387-960a-3256f5a1c3fd |
| 6904247d-56bf-4a0b-a318-a0cfb39d54de | https://portal.gdc.cancer.gov/files/6904247d-56bf-4a0b-a318-a0cfb39d54de |
| 692c76e6-7735-46d2-ae9f-a51a26418092 | https://portal.gdc.cancer.gov/files/692c76e6-7735-46d2-ae9f-a51a26418092 |
| 695be2a5-b838-4b7e-978d-5deeb5abab41 | https://portal.gdc.cancer.gov/files/695be2a5-b838-4b7e-978d-5deeb5abab41 |
| 69efabf3-dc51-403c-8984-b4253d0cff64 | https://portal.gdc.cancer.gov/files/69efabf3-dc51-403c-8984-b4253d0cff64 |
| 6b83908e-9bb0-42fe-af69-641bff5c3000 | https://portal.gdc.cancer.gov/files/6b83908e-9bb0-42fe-af69-641bff5c3000 |
| 6bb6bdd3-b0a4-4815-9593-a69b5a7d560c | https://portal.gdc.cancer.gov/files/6bb6bdd3-b0a4-4815-9593-a69b5a7d560c |
| 6cbd25bc-5145-49d9-b848-f1490bcb3092 | https://portal.gdc.cancer.gov/files/6cbd25bc-5145-49d9-b848-f1490bcb3092 |
| 6d19542b-3caa-49df-8ff1-156b1af2d3c9 | https://portal.gdc.cancer.gov/files/6d19542b-3caa-49df-8ff1-156b1af2d3c9 |
| 6d3aaf86-aba3-4341-8180-2dc5cd577356 | https://portal.gdc.cancer.gov/files/6d3aaf86-aba3-4341-8180-2dc5cd577356 |
| 6d47fe87-ed4c-463a-ae5b-2241d11b60cf | https://portal.gdc.cancer.gov/files/6d47fe87-ed4c-463a-ae5b-2241d11b60cf |
| 6ec05727-61e8-4acf-872f-f9782223565b | https://portal.gdc.cancer.gov/files/6ec05727-61e8-4acf-872f-f9782223565b |
| 6f0a4569-883a-48b3-a05e-33e667d09d99 | https://portal.gdc.cancer.gov/files/6f0a4569-883a-48b3-a05e-33e667d09d99 |
| 6f881286-3e20-4762-83e6-4c93f5383233 | https://portal.gdc.cancer.gov/files/6f881286-3e20-4762-83e6-4c93f5383233 |
| 6fe1dbfd-0310-4845-8d72-cdd464fce1bc | https://portal.gdc.cancer.gov/files/6fe1dbfd-0310-4845-8d72-cdd464fce1bc |
| 717323eb-f2ea-4228-9866-1e8992b410e2 | https://portal.gdc.cancer.gov/files/717323eb-f2ea-4228-9866-1e8992b410e2 |
| 71f052a2-9fb9-4f40-a7b0-224ce9c0c791 | https://portal.gdc.cancer.gov/files/71f052a2-9fb9-4f40-a7b0-224ce9c0c791 |
| 7209c651-73bc-4c5e-83e7-d2186915fa9f | https://portal.gdc.cancer.gov/files/7209c651-73bc-4c5e-83e7-d2186915fa9f |
| 72734c5d-1d55-4b8b-828e-a6add01cb131 | https://portal.gdc.cancer.gov/files/72734c5d-1d55-4b8b-828e-a6add01cb131 |
| 728eb336-d8e9-4030-abec-81d949ab7825 | https://portal.gdc.cancer.gov/files/728eb336-d8e9-4030-abec-81d949ab7825 |
| 7293b9db-416b-475e-b0ef-178c86b22582 | https://portal.gdc.cancer.gov/files/7293b9db-416b-475e-b0ef-178c86b22582 |
| 7472e636-4b83-4c56-bd35-76dfa1c08652 | https://portal.gdc.cancer.gov/files/7472e636-4b83-4c56-bd35-76dfa1c08652 |
| 757a247d-1538-4dc1-af55-a631a0d0c275 | https://portal.gdc.cancer.gov/files/757a247d-1538-4dc1-af55-a631a0d0c275 |
| 766bd94b-61e3-4f6d-a120-24106333252d | https://portal.gdc.cancer.gov/files/766bd94b-61e3-4f6d-a120-24106333252d |
| 76c230ce-a633-4bd6-b121-52668fe92d72 | https://portal.gdc.cancer.gov/files/76c230ce-a633-4bd6-b121-52668fe92d72 |
| 76c3ca99-7d4f-4f58-a026-563fc4724232 | https://portal.gdc.cancer.gov/files/76c3ca99-7d4f-4f58-a026-563fc4724232 |
| 77035a83-ca2f-4fa6-ab6a-afd0a5b779e5 | https://portal.gdc.cancer.gov/files/77035a83-ca2f-4fa6-ab6a-afd0a5b779e5 |
| 77306e98-fcb6-4052-bfba-cb4645280b19 | https://portal.gdc.cancer.gov/files/77306e98-fcb6-4052-bfba-cb4645280b19 |
| 781cf8da-d6a7-4dbe-9c91-419753f175ba | https://portal.gdc.cancer.gov/files/781cf8da-d6a7-4dbe-9c91-419753f175ba |
| 7adf17c6-eb53-42ad-8393-0c8d9ddabed9 | https://portal.gdc.cancer.gov/files/7adf17c6-eb53-42ad-8393-0c8d9ddabed9 |
| 7b82162c-8059-4326-afa9-1f96237e050e | https://portal.gdc.cancer.gov/files/7b82162c-8059-4326-afa9-1f96237e050e |
| 7b890ad8-511c-4256-b2cb-bb53471e22fd | https://portal.gdc.cancer.gov/files/7b890ad8-511c-4256-b2cb-bb53471e22fd |
| 7b8b406b-539a-4732-b214-cbc10c16ed55 | https://portal.gdc.cancer.gov/files/7b8b406b-539a-4732-b214-cbc10c16ed55 |
| 7ba0a8df-d5fd-48b7-8c31-9ded7c1a1948 | https://portal.gdc.cancer.gov/files/7ba0a8df-d5fd-48b7-8c31-9ded7c1a1948 |
| 7c17af06-2775-47b1-832b-c07ce1b61fb0 | https://portal.gdc.cancer.gov/files/7c17af06-2775-47b1-832b-c07ce1b61fb0 |
| 7cded4a3-2fee-4142-ad23-d6da4660adb0 | https://portal.gdc.cancer.gov/files/7cded4a3-2fee-4142-ad23-d6da4660adb0 |
| 7d41c2e2-6755-471a-aafd-7e2be5b87804 | https://portal.gdc.cancer.gov/files/7d41c2e2-6755-471a-aafd-7e2be5b87804 |
| 7df4e088-87c8-4ced-bc11-cb5f3dd4a9e0 | https://portal.gdc.cancer.gov/files/7df4e088-87c8-4ced-bc11-cb5f3dd4a9e0 |
| 7fe62c4f-03d6-4e14-b2c7-93bca6763bc4 | https://portal.gdc.cancer.gov/files/7fe62c4f-03d6-4e14-b2c7-93bca6763bc4 |
| 8030cb85-093f-4239-92d7-2739f5f5061a | https://portal.gdc.cancer.gov/files/8030cb85-093f-4239-92d7-2739f5f5061a |
| 807f276b-509b-4d4f-80ab-f7189c9f98a4 | https://portal.gdc.cancer.gov/files/807f276b-509b-4d4f-80ab-f7189c9f98a4 |
| 80daefe1-ffa9-4210-8684-323ebbe2b0fc | https://portal.gdc.cancer.gov/files/80daefe1-ffa9-4210-8684-323ebbe2b0fc |
| 8120a9c8-89bd-4ecb-a16a-b552e7ebade5 | https://portal.gdc.cancer.gov/files/8120a9c8-89bd-4ecb-a16a-b552e7ebade5 |
| 83790403-696c-4ad8-bd8a-20188fe8809d | https://portal.gdc.cancer.gov/files/83790403-696c-4ad8-bd8a-20188fe8809d |
| 8603af45-7a92-4d4f-975a-f4f8827824ff | https://portal.gdc.cancer.gov/files/8603af45-7a92-4d4f-975a-f4f8827824ff |
| 8605f3fc-b6da-4fff-8bb7-5aa8110f2aa3 | https://portal.gdc.cancer.gov/files/8605f3fc-b6da-4fff-8bb7-5aa8110f2aa3 |
| 86ae80d1-91a6-4c16-bba6-7ae4f1cc380d | https://portal.gdc.cancer.gov/files/86ae80d1-91a6-4c16-bba6-7ae4f1cc380d |
| 87fde345-e223-46f0-8fff-ecb31f51e12e | https://portal.gdc.cancer.gov/files/87fde345-e223-46f0-8fff-ecb31f51e12e |
| 88669db2-291f-4cf4-bdac-0b08243714a5 | https://portal.gdc.cancer.gov/files/88669db2-291f-4cf4-bdac-0b08243714a5 |
| 889b5805-701b-463b-9033-d31caedcd8a9 | https://portal.gdc.cancer.gov/files/889b5805-701b-463b-9033-d31caedcd8a9 |
| 8b2db0ed-d3db-42aa-a035-822632c5e1d6 | https://portal.gdc.cancer.gov/files/8b2db0ed-d3db-42aa-a035-822632c5e1d6 |
| 8d2ab605-027b-4302-b80d-88cee6684cbb | https://portal.gdc.cancer.gov/files/8d2ab605-027b-4302-b80d-88cee6684cbb |
| 8e86e1b7-d70d-4dd1-ac86-d344980a3e07 | https://portal.gdc.cancer.gov/files/8e86e1b7-d70d-4dd1-ac86-d344980a3e07 |
| 8ff8f6df-09e3-48e6-9d58-7d6bc8154a00 | https://portal.gdc.cancer.gov/files/8ff8f6df-09e3-48e6-9d58-7d6bc8154a00 |
| 9006e8da-716d-4d04-b383-f20f08c870f1 | https://portal.gdc.cancer.gov/files/9006e8da-716d-4d04-b383-f20f08c870f1 |
| 90193eb6-d10e-472d-9936-5dfda16805cd | https://portal.gdc.cancer.gov/files/90193eb6-d10e-472d-9936-5dfda16805cd |
| 911bb87f-0c71-4108-8c49-f56ad993aabf | https://portal.gdc.cancer.gov/files/911bb87f-0c71-4108-8c49-f56ad993aabf |
| 9164e835-918a-4483-83b3-6c39183bcf9a | https://portal.gdc.cancer.gov/files/9164e835-918a-4483-83b3-6c39183bcf9a |
| 91c6b658-b76c-4985-b364-ca4568e5ec68 | https://portal.gdc.cancer.gov/files/91c6b658-b76c-4985-b364-ca4568e5ec68 |
| 9211672d-1649-4786-98ae-2ff4a2a2bec9 | https://portal.gdc.cancer.gov/files/9211672d-1649-4786-98ae-2ff4a2a2bec9 |
| 92d0f433-b875-444d-9aa0-958918880e4c | https://portal.gdc.cancer.gov/files/92d0f433-b875-444d-9aa0-958918880e4c |
| 92d47ef6-2cd6-4599-b157-ab211ec968fa | https://portal.gdc.cancer.gov/files/92d47ef6-2cd6-4599-b157-ab211ec968fa |
| 9344b98a-5635-40cc-8dcf-e7bdfb9bde84 | https://portal.gdc.cancer.gov/files/9344b98a-5635-40cc-8dcf-e7bdfb9bde84 |
| 93fbb716-6e3e-4784-bce1-ed78bfd90686 | https://portal.gdc.cancer.gov/files/93fbb716-6e3e-4784-bce1-ed78bfd90686 |
| 945621af-6110-457e-b2bb-af6ce1d72352 | https://portal.gdc.cancer.gov/files/945621af-6110-457e-b2bb-af6ce1d72352 |
| 94ebf073-9c9d-4a35-b095-4fbd8171ebff | https://portal.gdc.cancer.gov/files/94ebf073-9c9d-4a35-b095-4fbd8171ebff |
| 959b1969-9eea-42af-8f46-d9b4ded060b3 | https://portal.gdc.cancer.gov/files/959b1969-9eea-42af-8f46-d9b4ded060b3 |
| 95cf4de6-30e8-42fd-a799-8664a4193286 | https://portal.gdc.cancer.gov/files/95cf4de6-30e8-42fd-a799-8664a4193286 |
| 9609e3f3-661d-48d2-a1d6-a51e4f83159e | https://portal.gdc.cancer.gov/files/9609e3f3-661d-48d2-a1d6-a51e4f83159e |
| 96ef9201-465c-4dfd-833f-e2f4bdb5ef65 | https://portal.gdc.cancer.gov/files/96ef9201-465c-4dfd-833f-e2f4bdb5ef65 |
| 985f21e7-6ccd-4b2d-a81d-e0106a3ef72a | https://portal.gdc.cancer.gov/files/985f21e7-6ccd-4b2d-a81d-e0106a3ef72a |
| 99b5f28a-033f-40c6-8c5f-469325b6bb37 | https://portal.gdc.cancer.gov/files/99b5f28a-033f-40c6-8c5f-469325b6bb37 |
| 9a015df6-d594-402a-9b87-ebf66c144ceb | https://portal.gdc.cancer.gov/files/9a015df6-d594-402a-9b87-ebf66c144ceb |
| 9b8e893f-3cfb-450c-bd42-d9c819d1dd57 | https://portal.gdc.cancer.gov/files/9b8e893f-3cfb-450c-bd42-d9c819d1dd57 |
| 9cbfb435-4ed0-4780-8750-5e72dce19891 | https://portal.gdc.cancer.gov/files/9cbfb435-4ed0-4780-8750-5e72dce19891 |
| 9dc96dfd-b44e-4682-8bfd-5088ab38bea5 | https://portal.gdc.cancer.gov/files/9dc96dfd-b44e-4682-8bfd-5088ab38bea5 |
| 9e2facc1-8e9b-4fc6-8f55-dc589e12b73d | https://portal.gdc.cancer.gov/files/9e2facc1-8e9b-4fc6-8f55-dc589e12b73d |
| 9e6b7d3f-0093-4c05-a3c6-63259a128ece | https://portal.gdc.cancer.gov/files/9e6b7d3f-0093-4c05-a3c6-63259a128ece |
| 9f204c48-6ab7-492e-91b3-0b7d65a23141 | https://portal.gdc.cancer.gov/files/9f204c48-6ab7-492e-91b3-0b7d65a23141 |
| 9fe92d43-d850-43ed-b222-6c8b17307faf | https://portal.gdc.cancer.gov/files/9fe92d43-d850-43ed-b222-6c8b17307faf |
| a16bc24b-0395-48ed-a160-f0311f97d49d | https://portal.gdc.cancer.gov/files/a16bc24b-0395-48ed-a160-f0311f97d49d |
| a1add3e1-3e1c-4b7f-8feb-61f05f1ef0e3 | https://portal.gdc.cancer.gov/files/a1add3e1-3e1c-4b7f-8feb-61f05f1ef0e3 |
| a24d3e36-0791-4bfb-94ad-f592cd72c5b4 | https://portal.gdc.cancer.gov/files/a24d3e36-0791-4bfb-94ad-f592cd72c5b4 |
| a2a561aa-0a43-45fb-92be-06a974066e2d | https://portal.gdc.cancer.gov/files/a2a561aa-0a43-45fb-92be-06a974066e2d |
| a338dad0-868b-42fc-9f30-1dd70ab6d7e8 | https://portal.gdc.cancer.gov/files/a338dad0-868b-42fc-9f30-1dd70ab6d7e8 |
| a3d9dae7-871c-450f-8646-f39cbfbbb1e4 | https://portal.gdc.cancer.gov/files/a3d9dae7-871c-450f-8646-f39cbfbbb1e4 |
| a4a438c0-759b-48ff-afd1-5dcac0da4639 | https://portal.gdc.cancer.gov/files/a4a438c0-759b-48ff-afd1-5dcac0da4639 |
| a4b6946c-4c48-423f-87c7-cb1a7f92985a | https://portal.gdc.cancer.gov/files/a4b6946c-4c48-423f-87c7-cb1a7f92985a |
| a51027e4-4c22-47ff-b659-d5fb333bb12b | https://portal.gdc.cancer.gov/files/a51027e4-4c22-47ff-b659-d5fb333bb12b |
| a5386459-de03-41ba-9949-21e5bd3adb42 | https://portal.gdc.cancer.gov/files/a5386459-de03-41ba-9949-21e5bd3adb42 |
| a5645f54-56d9-4cc6-a694-712c336d0013 | https://portal.gdc.cancer.gov/files/a5645f54-56d9-4cc6-a694-712c336d0013 |
| a57b3b6a-d4c5-4bd2-a6a3-11e604587645 | https://portal.gdc.cancer.gov/files/a57b3b6a-d4c5-4bd2-a6a3-11e604587645 |
| a5d96805-d4cf-40b3-bde1-9a0fa8bbb617 | https://portal.gdc.cancer.gov/files/a5d96805-d4cf-40b3-bde1-9a0fa8bbb617 |
| a5ecffca-561f-4493-a00f-a2a798c72a2e | https://portal.gdc.cancer.gov/files/a5ecffca-561f-4493-a00f-a2a798c72a2e |
| a626ce03-2f34-48e2-8c13-c5ba87e43f59 | https://portal.gdc.cancer.gov/files/a626ce03-2f34-48e2-8c13-c5ba87e43f59 |
| a7ec4958-96d9-4e39-b9a5-0a669ce537ac | https://portal.gdc.cancer.gov/files/a7ec4958-96d9-4e39-b9a5-0a669ce537ac |
| a825b2a6-90ac-4bc4-a8ff-e2927ee8ee11 | https://portal.gdc.cancer.gov/files/a825b2a6-90ac-4bc4-a8ff-e2927ee8ee11 |
| a897d038-176c-4cd4-a121-c568539776bb | https://portal.gdc.cancer.gov/files/a897d038-176c-4cd4-a121-c568539776bb |
| a8cfe5bb-4a97-48d2-a399-c741a0c68389 | https://portal.gdc.cancer.gov/files/a8cfe5bb-4a97-48d2-a399-c741a0c68389 |
| aac7cc14-0158-40b6-8281-ea732aa12ff0 | https://portal.gdc.cancer.gov/files/aac7cc14-0158-40b6-8281-ea732aa12ff0 |
| aaf1c822-1773-4164-9157-68e49f59cc60 | https://portal.gdc.cancer.gov/files/aaf1c822-1773-4164-9157-68e49f59cc60 |
| ab854d2f-ad34-4912-88e9-c24e55a021e6 | https://portal.gdc.cancer.gov/files/ab854d2f-ad34-4912-88e9-c24e55a021e6 |
| abb12a26-edd1-4908-9499-8604c1b3f592 | https://portal.gdc.cancer.gov/files/abb12a26-edd1-4908-9499-8604c1b3f592 |
| abbde3ee-4baf-4e94-b5ab-44b23d51f410 | https://portal.gdc.cancer.gov/files/abbde3ee-4baf-4e94-b5ab-44b23d51f410 |
| abf0a507-8303-4d29-8fc4-d39178a4d2d0 | https://portal.gdc.cancer.gov/files/abf0a507-8303-4d29-8fc4-d39178a4d2d0 |
| aea013c4-50b6-458d-b78f-5bc84f4ccd10 | https://portal.gdc.cancer.gov/files/aea013c4-50b6-458d-b78f-5bc84f4ccd10 |
| af1f0367-9ae8-4299-b9c8-dda91153e26c | https://portal.gdc.cancer.gov/files/af1f0367-9ae8-4299-b9c8-dda91153e26c |
| af873590-36ff-4947-ab8e-e07f734a2009 | https://portal.gdc.cancer.gov/files/af873590-36ff-4947-ab8e-e07f734a2009 |
| b00ea70b-7afc-4514-af8c-6e4451093120 | https://portal.gdc.cancer.gov/files/b00ea70b-7afc-4514-af8c-6e4451093120 |
| b1356ec6-5ae7-48b1-942f-a6941db8e996 | https://portal.gdc.cancer.gov/files/b1356ec6-5ae7-48b1-942f-a6941db8e996 |
| b1958f44-3cfa-4115-b715-944a23cbe24f | https://portal.gdc.cancer.gov/files/b1958f44-3cfa-4115-b715-944a23cbe24f |
| b2275997-88fc-4b27-a93a-21fe63482efa | https://portal.gdc.cancer.gov/files/b2275997-88fc-4b27-a93a-21fe63482efa |
| b25654c8-46ae-4b95-8406-6141ff5c1dda | https://portal.gdc.cancer.gov/files/b25654c8-46ae-4b95-8406-6141ff5c1dda |
| b30d7b80-85ab-4aab-961c-27a071cde022 | https://portal.gdc.cancer.gov/files/b30d7b80-85ab-4aab-961c-27a071cde022 |
| b453eaa5-c870-4b57-89bc-c2795967141d | https://portal.gdc.cancer.gov/files/b453eaa5-c870-4b57-89bc-c2795967141d |
| b49a5e18-5eca-4ba0-8eda-2489c3d6fe62 | https://portal.gdc.cancer.gov/files/b49a5e18-5eca-4ba0-8eda-2489c3d6fe62 |
| b4be9dcd-84ea-4ee1-ba70-24002c04c013 | https://portal.gdc.cancer.gov/files/b4be9dcd-84ea-4ee1-ba70-24002c04c013 |
| b4f73fdb-f757-4c42-8cd6-67892b87408d | https://portal.gdc.cancer.gov/files/b4f73fdb-f757-4c42-8cd6-67892b87408d |
| b546c8c7-9b30-4903-8752-541e4402f9ab | https://portal.gdc.cancer.gov/files/b546c8c7-9b30-4903-8752-541e4402f9ab |
| b5ab168b-a617-4152-9ac9-58287b94e44e | https://portal.gdc.cancer.gov/files/b5ab168b-a617-4152-9ac9-58287b94e44e |
| b661aca1-348e-4a67-a7ff-e8a8923db238 | https://portal.gdc.cancer.gov/files/b661aca1-348e-4a67-a7ff-e8a8923db238 |
| b84f6894-75fe-42de-874a-95eba81116b5 | https://portal.gdc.cancer.gov/files/b84f6894-75fe-42de-874a-95eba81116b5 |
| b86c1d0a-c3fe-4006-a056-7fe75a8adf2e | https://portal.gdc.cancer.gov/files/b86c1d0a-c3fe-4006-a056-7fe75a8adf2e |
| b86e779a-0194-4277-b2a8-68b76e76be9d | https://portal.gdc.cancer.gov/files/b86e779a-0194-4277-b2a8-68b76e76be9d |
| b8f5ce02-1f81-4af0-b252-4bdf249d6bba | https://portal.gdc.cancer.gov/files/b8f5ce02-1f81-4af0-b252-4bdf249d6bba |
| ba17c2a4-a8f3-4947-bd31-893aac096943 | https://portal.gdc.cancer.gov/files/ba17c2a4-a8f3-4947-bd31-893aac096943 |
| ba684bfb-0121-4e6d-80a4-e160a1fe4008 | https://portal.gdc.cancer.gov/files/ba684bfb-0121-4e6d-80a4-e160a1fe4008 |
| bcdb1b4f-738a-470f-b445-e645dea28831 | https://portal.gdc.cancer.gov/files/bcdb1b4f-738a-470f-b445-e645dea28831 |
| bd89eb5a-4e0a-474c-b9fe-1a2a50749310 | https://portal.gdc.cancer.gov/files/bd89eb5a-4e0a-474c-b9fe-1a2a50749310 |
| bd939197-9484-4a48-8611-8b1d23b8d198 | https://portal.gdc.cancer.gov/files/bd939197-9484-4a48-8611-8b1d23b8d198 |
| beb48cb5-d3e2-45d3-bb3d-6994bb8a7fca | https://portal.gdc.cancer.gov/files/beb48cb5-d3e2-45d3-bb3d-6994bb8a7fca |
| c029d08f-a8e2-47b3-8768-fa944a0d1899 | https://portal.gdc.cancer.gov/files/c029d08f-a8e2-47b3-8768-fa944a0d1899 |
| c1d89b0b-5f79-4769-88c3-156abae25998 | https://portal.gdc.cancer.gov/files/c1d89b0b-5f79-4769-88c3-156abae25998 |
| c341cc55-7e29-4a27-93d4-ad470aaf08a2 | https://portal.gdc.cancer.gov/files/c341cc55-7e29-4a27-93d4-ad470aaf08a2 |
| c37933d5-e904-4177-a252-0684cb5dc019 | https://portal.gdc.cancer.gov/files/c37933d5-e904-4177-a252-0684cb5dc019 |
| c4c82cd5-1b45-4ea3-937b-e20c9081cb51 | https://portal.gdc.cancer.gov/files/c4c82cd5-1b45-4ea3-937b-e20c9081cb51 |
| c4e00540-c80a-43e9-9a6c-964283699be7 | https://portal.gdc.cancer.gov/files/c4e00540-c80a-43e9-9a6c-964283699be7 |
| c5076feb-831e-4c93-b8a3-ce85c196ac3a | https://portal.gdc.cancer.gov/files/c5076feb-831e-4c93-b8a3-ce85c196ac3a |
| c717350c-918f-43bf-940a-b1a5e8426a1b | https://portal.gdc.cancer.gov/files/c717350c-918f-43bf-940a-b1a5e8426a1b |
| c7ed1e9a-9649-4254-9132-5dc7ad7fc285 | https://portal.gdc.cancer.gov/files/c7ed1e9a-9649-4254-9132-5dc7ad7fc285 |
| c7f27515-e164-48c5-a2c9-762f9e8361b4 | https://portal.gdc.cancer.gov/files/c7f27515-e164-48c5-a2c9-762f9e8361b4 |
| c80025cc-87e8-4aea-a051-dc16e4a4c22c | https://portal.gdc.cancer.gov/files/c80025cc-87e8-4aea-a051-dc16e4a4c22c |
| c8f2283c-2eca-4027-9a5c-6a38533e58cf | https://portal.gdc.cancer.gov/files/c8f2283c-2eca-4027-9a5c-6a38533e58cf |
| c976993e-6c63-46bd-bd75-a3839da5b2c1 | https://portal.gdc.cancer.gov/files/c976993e-6c63-46bd-bd75-a3839da5b2c1 |
| c9965399-7b87-49fe-9b74-18e2b8666a66 | https://portal.gdc.cancer.gov/files/c9965399-7b87-49fe-9b74-18e2b8666a66 |
| c9c6122a-0432-46e1-b538-4650b5f896ef | https://portal.gdc.cancer.gov/files/c9c6122a-0432-46e1-b538-4650b5f896ef |
| caf7132d-c0cf-43d5-ad64-71b721ca5e04 | https://portal.gdc.cancer.gov/files/caf7132d-c0cf-43d5-ad64-71b721ca5e04 |
| cafb16b1-4f7c-4203-9acc-15bc147b9a3e | https://portal.gdc.cancer.gov/files/cafb16b1-4f7c-4203-9acc-15bc147b9a3e |
| cb0398c8-832f-4224-b69f-ae34dc0188c0 | https://portal.gdc.cancer.gov/files/cb0398c8-832f-4224-b69f-ae34dc0188c0 |
| cb208a43-3c6f-4afa-8ec1-44588ee97893 | https://portal.gdc.cancer.gov/files/cb208a43-3c6f-4afa-8ec1-44588ee97893 |
| cbaedd9f-0ec7-4eea-9de1-45c39102b46d | https://portal.gdc.cancer.gov/files/cbaedd9f-0ec7-4eea-9de1-45c39102b46d |
| cbcc52d9-11dd-49f7-b57e-3cd695ade61f | https://portal.gdc.cancer.gov/files/cbcc52d9-11dd-49f7-b57e-3cd695ade61f |
| cbe1cf7e-5a6e-495f-abd0-97c15852b7b4 | https://portal.gdc.cancer.gov/files/cbe1cf7e-5a6e-495f-abd0-97c15852b7b4 |
| cc58aa12-3394-409c-a746-3e8778e6c044 | https://portal.gdc.cancer.gov/files/cc58aa12-3394-409c-a746-3e8778e6c044 |
| cc7dac88-7689-4cd4-8cbc-53083b49713f | https://portal.gdc.cancer.gov/files/cc7dac88-7689-4cd4-8cbc-53083b49713f |
| ccfd06b0-e27a-4023-87f7-870d6d6c93c5 | https://portal.gdc.cancer.gov/files/ccfd06b0-e27a-4023-87f7-870d6d6c93c5 |
| cd54927f-3f15-4e13-9865-4145732a87b9 | https://portal.gdc.cancer.gov/files/cd54927f-3f15-4e13-9865-4145732a87b9 |
| cec61710-e12a-4e52-809f-58f0b054b1ae | https://portal.gdc.cancer.gov/files/cec61710-e12a-4e52-809f-58f0b054b1ae |
| d0cf0124-3a28-42f6-9629-cb0792d8c47c | https://portal.gdc.cancer.gov/files/d0cf0124-3a28-42f6-9629-cb0792d8c47c |
| d149b731-1265-4dc1-8384-a69f8849552d | https://portal.gdc.cancer.gov/files/d149b731-1265-4dc1-8384-a69f8849552d |
| d2605ecf-1b3b-43bd-8435-f6bb8ead5491 | https://portal.gdc.cancer.gov/files/d2605ecf-1b3b-43bd-8435-f6bb8ead5491 |
| d2af4e85-fb4b-481d-803b-645e3b1cb7db | https://portal.gdc.cancer.gov/files/d2af4e85-fb4b-481d-803b-645e3b1cb7db |
| d34d0c9f-a642-4baa-b9e9-5155cafb36cb | https://portal.gdc.cancer.gov/files/d34d0c9f-a642-4baa-b9e9-5155cafb36cb |
| d3581b98-3cfc-455d-ba2a-5428e2118270 | https://portal.gdc.cancer.gov/files/d3581b98-3cfc-455d-ba2a-5428e2118270 |
| d39cec3b-99eb-4010-8a5a-af551e650bf5 | https://portal.gdc.cancer.gov/files/d39cec3b-99eb-4010-8a5a-af551e650bf5 |
| d4a06034-228a-450b-bd4e-718d7dce7786 | https://portal.gdc.cancer.gov/files/d4a06034-228a-450b-bd4e-718d7dce7786 |
| d61c0d0c-23df-4db0-8153-f383be0a54d6 | https://portal.gdc.cancer.gov/files/d61c0d0c-23df-4db0-8153-f383be0a54d6 |
| d7371312-6cfd-4f30-b026-bca837ee32fd | https://portal.gdc.cancer.gov/files/d7371312-6cfd-4f30-b026-bca837ee32fd |
| d7cd35ed-081b-4597-8665-3fc23d8a81e3 | https://portal.gdc.cancer.gov/files/d7cd35ed-081b-4597-8665-3fc23d8a81e3 |
| d939c38a-6e77-4ee7-8f8f-1107d8dc5408 | https://portal.gdc.cancer.gov/files/d939c38a-6e77-4ee7-8f8f-1107d8dc5408 |
| d9fa5400-083c-4c09-9b35-93dee3968134 | https://portal.gdc.cancer.gov/files/d9fa5400-083c-4c09-9b35-93dee3968134 |
| da2bc67e-2f85-4717-9a0c-59caa7c918d9 | https://portal.gdc.cancer.gov/files/da2bc67e-2f85-4717-9a0c-59caa7c918d9 |
| db30c205-5a70-4e04-991e-5f76a0f09c8c | https://portal.gdc.cancer.gov/files/db30c205-5a70-4e04-991e-5f76a0f09c8c |
| db82b8d0-0392-421e-8dce-39872cf93caa | https://portal.gdc.cancer.gov/files/db82b8d0-0392-421e-8dce-39872cf93caa |
| dc5a1c72-96cf-473f-8a92-57ea10250230 | https://portal.gdc.cancer.gov/files/dc5a1c72-96cf-473f-8a92-57ea10250230 |
| dde88420-ba9a-409b-935f-8deae5b3581a | https://portal.gdc.cancer.gov/files/dde88420-ba9a-409b-935f-8deae5b3581a |
| de26cd8c-3b04-4376-a860-336acc18de12 | https://portal.gdc.cancer.gov/files/de26cd8c-3b04-4376-a860-336acc18de12 |
| df47d711-d515-404b-b912-1697f57b97f8 | https://portal.gdc.cancer.gov/files/df47d711-d515-404b-b912-1697f57b97f8 |
| df74ca57-4f5c-4303-8fd9-1eb0445d377c | https://portal.gdc.cancer.gov/files/df74ca57-4f5c-4303-8fd9-1eb0445d377c |
| e0cf808e-f2c5-4755-9d57-d26d638c5668 | https://portal.gdc.cancer.gov/files/e0cf808e-f2c5-4755-9d57-d26d638c5668 |
| e12fafa6-6e40-47b3-9232-84b3b6c72ee7 | https://portal.gdc.cancer.gov/files/e12fafa6-6e40-47b3-9232-84b3b6c72ee7 |
| e197651e-db2b-4eba-8634-e73194ebdef8 | https://portal.gdc.cancer.gov/files/e197651e-db2b-4eba-8634-e73194ebdef8 |
| e1d552e6-301b-45f6-b505-3b02c4b243e8 | https://portal.gdc.cancer.gov/files/e1d552e6-301b-45f6-b505-3b02c4b243e8 |
| e2423df3-bbd4-4fcc-a6db-bb54eb62ede9 | https://portal.gdc.cancer.gov/files/e2423df3-bbd4-4fcc-a6db-bb54eb62ede9 |
| e3a5cc08-ee0f-44b2-885d-07725b32eb17 | https://portal.gdc.cancer.gov/files/e3a5cc08-ee0f-44b2-885d-07725b32eb17 |
| e44f6812-bc25-456f-b78a-43b7db2a2180 | https://portal.gdc.cancer.gov/files/e44f6812-bc25-456f-b78a-43b7db2a2180 |
| e45a74a7-a35e-4bf5-861b-b6fa20e7d692 | https://portal.gdc.cancer.gov/files/e45a74a7-a35e-4bf5-861b-b6fa20e7d692 |
| e4f087a6-57d7-43d9-a0ac-17761795a467 | https://portal.gdc.cancer.gov/files/e4f087a6-57d7-43d9-a0ac-17761795a467 |
| e5cf0968-7f8a-47c4-a0b2-e15586bcd9c3 | https://portal.gdc.cancer.gov/files/e5cf0968-7f8a-47c4-a0b2-e15586bcd9c3 |
| e68e4c57-b9be-4580-a5d8-2a7fb39c1dd2 | https://portal.gdc.cancer.gov/files/e68e4c57-b9be-4580-a5d8-2a7fb39c1dd2 |
| e7ec48de-8c9e-4cdf-a6bf-ba5b51b7e08d | https://portal.gdc.cancer.gov/files/e7ec48de-8c9e-4cdf-a6bf-ba5b51b7e08d |
| e9177c5c-609e-44b3-903e-669f1adecdc7 | https://portal.gdc.cancer.gov/files/e9177c5c-609e-44b3-903e-669f1adecdc7 |
| eb9f8d28-1972-4ece-90a0-7f31f7eef259 | https://portal.gdc.cancer.gov/files/eb9f8d28-1972-4ece-90a0-7f31f7eef259 |
| ebf470dc-a43d-4635-bbd2-7b79f0ee1652 | https://portal.gdc.cancer.gov/files/ebf470dc-a43d-4635-bbd2-7b79f0ee1652 |
| ec83bf2b-43f4-4ee2-8275-ac195cf061b4 | https://portal.gdc.cancer.gov/files/ec83bf2b-43f4-4ee2-8275-ac195cf061b4 |
| ecd1b4c1-12e9-4fbe-8bb3-af05da62271f | https://portal.gdc.cancer.gov/files/ecd1b4c1-12e9-4fbe-8bb3-af05da62271f |
| ed56526b-1386-4701-912a-320288d3aed1 | https://portal.gdc.cancer.gov/files/ed56526b-1386-4701-912a-320288d3aed1 |
| ed59c0b9-8fbd-4ef1-8483-efe5429c13cf | https://portal.gdc.cancer.gov/files/ed59c0b9-8fbd-4ef1-8483-efe5429c13cf |
| edbbe745-0d2c-4bca-951b-e53c20756fcd | https://portal.gdc.cancer.gov/files/edbbe745-0d2c-4bca-951b-e53c20756fcd |
| eed590d5-3784-4dac-8cea-4c6ce7a57321 | https://portal.gdc.cancer.gov/files/eed590d5-3784-4dac-8cea-4c6ce7a57321 |
| ef84638e-e040-4cbd-a506-5b78d4a74754 | https://portal.gdc.cancer.gov/files/ef84638e-e040-4cbd-a506-5b78d4a74754 |
| f046fc7e-04b2-4a68-af02-6125a7043d88 | https://portal.gdc.cancer.gov/files/f046fc7e-04b2-4a68-af02-6125a7043d88 |
| f0f6c497-4fef-4502-a5a2-42284ed792fa | https://portal.gdc.cancer.gov/files/f0f6c497-4fef-4502-a5a2-42284ed792fa |
| f1360de4-ce7b-46d2-af27-3fcd2ec9685c | https://portal.gdc.cancer.gov/files/f1360de4-ce7b-46d2-af27-3fcd2ec9685c |
| f22afe0d-0733-4640-b95f-7bc17fcd3e72 | https://portal.gdc.cancer.gov/files/f22afe0d-0733-4640-b95f-7bc17fcd3e72 |
| f2668e01-d02f-409a-92e3-27a2936d68ac | https://portal.gdc.cancer.gov/files/f2668e01-d02f-409a-92e3-27a2936d68ac |
| f2c5c5b9-1d8f-4609-818a-a5efa9d8db9f | https://portal.gdc.cancer.gov/files/f2c5c5b9-1d8f-4609-818a-a5efa9d8db9f |
| f3fefe95-3d18-4c65-bc3a-b6db22696552 | https://portal.gdc.cancer.gov/files/f3fefe95-3d18-4c65-bc3a-b6db22696552 |
| f41757eb-7766-49e4-9813-11742c3baf84 | https://portal.gdc.cancer.gov/files/f41757eb-7766-49e4-9813-11742c3baf84 |
| f43f89b7-5c84-4736-9b7c-80e48e3fd683 | https://portal.gdc.cancer.gov/files/f43f89b7-5c84-4736-9b7c-80e48e3fd683 |
| f4e5eb72-f21d-4a87-8237-83ded624f82e | https://portal.gdc.cancer.gov/files/f4e5eb72-f21d-4a87-8237-83ded624f82e |
| f53c28eb-97a7-4347-bad1-e7afa85b5a94 | https://portal.gdc.cancer.gov/files/f53c28eb-97a7-4347-bad1-e7afa85b5a94 |
| f5df9c67-2968-4fdf-a8f3-272af435eae5 | https://portal.gdc.cancer.gov/files/f5df9c67-2968-4fdf-a8f3-272af435eae5 |
| f66f10b1-474a-47a9-9452-537a663b732b | https://portal.gdc.cancer.gov/files/f66f10b1-474a-47a9-9452-537a663b732b |
| f68b398c-a141-4dea-a11b-824853c4e74c | https://portal.gdc.cancer.gov/files/f68b398c-a141-4dea-a11b-824853c4e74c |
| f6c99710-f900-4488-9ebb-fff65bf199c7 | https://portal.gdc.cancer.gov/files/f6c99710-f900-4488-9ebb-fff65bf199c7 |
| f6f7e125-a2b3-45f6-bbbd-6b840d33d8d9 | https://portal.gdc.cancer.gov/files/f6f7e125-a2b3-45f6-bbbd-6b840d33d8d9 |
| f7c0bf7a-3ee9-4969-b615-fa4682e067ae | https://portal.gdc.cancer.gov/files/f7c0bf7a-3ee9-4969-b615-fa4682e067ae |
| f94de29d-9127-4e49-bb34-a1fb554dcbbb | https://portal.gdc.cancer.gov/files/f94de29d-9127-4e49-bb34-a1fb554dcbbb |
| fa93550f-8849-45ca-81bf-eb7d2e4b3e9b | https://portal.gdc.cancer.gov/files/fa93550f-8849-45ca-81bf-eb7d2e4b3e9b |
| fb06e192-2cb7-46e4-a8be-b4ab6ae50ffb | https://portal.gdc.cancer.gov/files/fb06e192-2cb7-46e4-a8be-b4ab6ae50ffb |
| fb8b8859-048b-4f70-8f0a-9aff70afebf8 | https://portal.gdc.cancer.gov/files/fb8b8859-048b-4f70-8f0a-9aff70afebf8 |
| fbd79fc2-02c8-444f-af1f-2b63f28f5126 | https://portal.gdc.cancer.gov/files/fbd79fc2-02c8-444f-af1f-2b63f28f5126 |
| fbdafcb0-98ff-4c52-bfe5-be7d54ff9d9e | https://portal.gdc.cancer.gov/files/fbdafcb0-98ff-4c52-bfe5-be7d54ff9d9e |
| fc1bab6c-9e7d-4479-8f65-064f46cae30f | https://portal.gdc.cancer.gov/files/fc1bab6c-9e7d-4479-8f65-064f46cae30f |
| fc9fd510-778e-40ea-9add-114e00b35e35 | https://portal.gdc.cancer.gov/files/fc9fd510-778e-40ea-9add-114e00b35e35 |
| fd9f3f66-16c2-491d-ab2e-5fdcea1d3333 | https://portal.gdc.cancer.gov/files/fd9f3f66-16c2-491d-ab2e-5fdcea1d3333 |
| fdc042a1-e443-4077-bd54-d4f131bf35a0 | https://portal.gdc.cancer.gov/files/fdc042a1-e443-4077-bd54-d4f131bf35a0 |
| fe254c35-b0ab-4527-93b7-3ffd928400a8 | https://portal.gdc.cancer.gov/files/fe254c35-b0ab-4527-93b7-3ffd928400a8 |
| fe4306d6-5713-436a-bcd5-ae538bb0ee4a | https://portal.gdc.cancer.gov/files/fe4306d6-5713-436a-bcd5-ae538bb0ee4a |
| ff4305ae-ba2a-4ecf-919c-0047bf7a27e0 | https://portal.gdc.cancer.gov/files/ff4305ae-ba2a-4ecf-919c-0047bf7a27e0 |
| ffc2c146-fe1d-4233-8d92-c207637d7bf2 | https://portal.gdc.cancer.gov/files/ffc2c146-fe1d-4233-8d92-c207637d7bf2 |
| fffd310d-a49e-44c6-b6de-07cda0255156 | https://portal.gdc.cancer.gov/files/fffd310d-a49e-44c6-b6de-07cda0255156 |
