# Supplementary material for: Prognostic prediction and immune infiltration analysis based on ferroptosis and EMT state in hepatocellular carcinoma
Source: Front Immunol. 2022 Dec 15;13:1076045. doi: 10.3389/fimmu.2022.1076045 (PMC9797854; doi:10.3389/fimmu.2022.1076045)
Supplement: Supplementary file 5 [file Table_4.docx]

**Supplementary table 4** The donor id (n=232) of HCC patients from the ICGC website.

| Donor id | links |
| --- | --- |
| DO45299 | <https://dcc.icgc.org/donors/DO45299> |
| DO48682 | <https://dcc.icgc.org/donors/DO48682> |
| DO48719 | <https://dcc.icgc.org/donors/DO48719> |
| DO45096 | https://dcc.icgc.org/donors/DO45096 |
| DO50793 | https://dcc.icgc.org/donors/DO50793 |
| DO50814 | https://dcc.icgc.org/donors/DO50814 |
| DO23543 | https://dcc.icgc.org/donors/DO23543 |
| DO45139 | https://dcc.icgc.org/donors/DO45139 |
| DO45237 | https://dcc.icgc.org/donors/DO45237 |
| DO45127 | https://dcc.icgc.org/donors/DO45127 |
| DO50844 | https://dcc.icgc.org/donors/DO50844 |
| DO23516 | https://dcc.icgc.org/donors/DO23516 |
| DO50804 | https://dcc.icgc.org/donors/DO50804 |
| DO45119 | https://dcc.icgc.org/donors/DO45119 |
| DO23508 | https://dcc.icgc.org/donors/DO23508 |
| DO48742 | https://dcc.icgc.org/donors/DO48742 |
| DO45267 | https://dcc.icgc.org/donors/DO45267 |
| DO45145 | https://dcc.icgc.org/donors/DO45145 |
| DO45141 | https://dcc.icgc.org/donors/DO45141 |
| DO48732 | https://dcc.icgc.org/donors/DO48732 |
| DO45091 | https://dcc.icgc.org/donors/DO45091 |
| DO23551 | https://dcc.icgc.org/donors/DO23551 |
| DO50803 | https://dcc.icgc.org/donors/DO50803 |
| DO45169 | https://dcc.icgc.org/donors/DO45169 |
| DO48706 | https://dcc.icgc.org/donors/DO48706 |
| DO45281 | https://dcc.icgc.org/donors/DO45281 |
| DO23525 | https://dcc.icgc.org/donors/DO23525 |
| DO50825 | https://dcc.icgc.org/donors/DO50825 |
| DO48716 | https://dcc.icgc.org/donors/DO48716 |
| DO50818 | https://dcc.icgc.org/donors/DO50818 |
| DO50798 | https://dcc.icgc.org/donors/DO50798 |
| DO23537 | https://dcc.icgc.org/donors/DO23537 |
| DO45273 | https://dcc.icgc.org/donors/DO45273 |
| DO23513 | https://dcc.icgc.org/donors/DO23513 |
| DO45094 | https://dcc.icgc.org/donors/DO45094 |
| DO23536 | https://dcc.icgc.org/donors/DO23536 |
| DO45259 | https://dcc.icgc.org/donors/DO45259 |
| DO50817 | https://dcc.icgc.org/donors/DO50817 |
| DO45163 | https://dcc.icgc.org/donors/DO45163 |
| DO45225 | https://dcc.icgc.org/donors/DO45225 |
| DO50776 | https://dcc.icgc.org/donors/DO50776 |
| DO45185 | https://dcc.icgc.org/donors/DO45185 |
| DO23540 | https://dcc.icgc.org/donors/DO23540 |
| DO50859 | https://dcc.icgc.org/donors/DO50859 |
| DO50805 | https://dcc.icgc.org/donors/DO50805 |
| DO23544 | https://dcc.icgc.org/donors/DO23544 |
| DO45111 | https://dcc.icgc.org/donors/DO45111 |
| DO48672 | https://dcc.icgc.org/donors/DO48672 |
| DO45115 | https://dcc.icgc.org/donors/DO45115 |
| DO45199 | https://dcc.icgc.org/donors/DO45199 |
| DO23545 | https://dcc.icgc.org/donors/DO23545 |
| DO45203 | https://dcc.icgc.org/donors/DO45203 |
| DO45207 | https://dcc.icgc.org/donors/DO45207 |
| DO48721 | https://dcc.icgc.org/donors/DO48721 |
| DO45153 | https://dcc.icgc.org/donors/DO45153 |
| DO48701 | https://dcc.icgc.org/donors/DO48701 |
| DO23533 | https://dcc.icgc.org/donors/DO23533 |
| DO50815 | https://dcc.icgc.org/donors/DO50815 |
| DO48677 | https://dcc.icgc.org/donors/DO48677 |
| DO23519 | https://dcc.icgc.org/donors/DO23519 |
| DO45193 | https://dcc.icgc.org/donors/DO45193 |
| DO45105 | https://dcc.icgc.org/donors/DO45105 |
| DO45117 | https://dcc.icgc.org/donors/DO45117 |
| DO45155 | https://dcc.icgc.org/donors/DO45155 |
| DO45173 | https://dcc.icgc.org/donors/DO45173 |
| DO48751 | https://dcc.icgc.org/donors/DO48751 |
| DO50806 | https://dcc.icgc.org/donors/DO50806 |
| DO23541 | https://dcc.icgc.org/donors/DO23541 |
| DO48741 | https://dcc.icgc.org/donors/DO48741 |
| DO48679 | https://dcc.icgc.org/donors/DO48679 |
| DO50813 | https://dcc.icgc.org/donors/DO50813 |
| DO23521 | https://dcc.icgc.org/donors/DO23521 |
| DO23509 | https://dcc.icgc.org/donors/DO23509 |
| DO48674 | https://dcc.icgc.org/donors/DO48674 |
| DO45161 | https://dcc.icgc.org/donors/DO45161 |
| DO50816 | https://dcc.icgc.org/donors/DO50816 |
| DO45092 | https://dcc.icgc.org/donors/DO45092 |
| DO48723 | https://dcc.icgc.org/donors/DO48723 |
| DO45277 | https://dcc.icgc.org/donors/DO45277 |
| DO48746 | https://dcc.icgc.org/donors/DO48746 |
| DO50832 | https://dcc.icgc.org/donors/DO50832 |
| DO48737 | https://dcc.icgc.org/donors/DO48737 |
| DO23542 | https://dcc.icgc.org/donors/DO23542 |
| DO50807 | https://dcc.icgc.org/donors/DO50807 |
| DO48760 | https://dcc.icgc.org/donors/DO48760 |
| DO23517 | https://dcc.icgc.org/donors/DO23517 |
| DO45269 | https://dcc.icgc.org/donors/DO45269 |
| DO45097 | https://dcc.icgc.org/donors/DO45097 |
| DO48695 | https://dcc.icgc.org/donors/DO48695 |
| DO23539 | https://dcc.icgc.org/donors/DO23539 |
| DO45133 | https://dcc.icgc.org/donors/DO45133 |
| DO45227 | https://dcc.icgc.org/donors/DO45227 |
| DO45213 | https://dcc.icgc.org/donors/DO45213 |
| DO48757 | https://dcc.icgc.org/donors/DO48757 |
| DO23512 | https://dcc.icgc.org/donors/DO23512 |
| DO45181 | https://dcc.icgc.org/donors/DO45181 |
| DO23514 | https://dcc.icgc.org/donors/DO23514 |
| DO23534 | https://dcc.icgc.org/donors/DO23534 |
| DO48681 | https://dcc.icgc.org/donors/DO48681 |
| DO50857 | https://dcc.icgc.org/donors/DO50857 |
| DO45197 | https://dcc.icgc.org/donors/DO45197 |
| DO50811 | https://dcc.icgc.org/donors/DO50811 |
| DO50822 | https://dcc.icgc.org/donors/DO50822 |
| DO45201 | https://dcc.icgc.org/donors/DO45201 |
| DO45175 | https://dcc.icgc.org/donors/DO45175 |
| DO45265 | https://dcc.icgc.org/donors/DO45265 |
| DO45107 | https://dcc.icgc.org/donors/DO45107 |
| DO45287 | https://dcc.icgc.org/donors/DO45287 |
| DO45159 | https://dcc.icgc.org/donors/DO45159 |
| DO23518 | https://dcc.icgc.org/donors/DO23518 |
| DO45205 | https://dcc.icgc.org/donors/DO45205 |
| DO50799 | https://dcc.icgc.org/donors/DO50799 |
| DO45123 | https://dcc.icgc.org/donors/DO45123 |
| DO45301 | https://dcc.icgc.org/donors/DO45301 |
| DO50802 | https://dcc.icgc.org/donors/DO50802 |
| DO23548 | https://dcc.icgc.org/donors/DO23548 |
| DO45229 | https://dcc.icgc.org/donors/DO45229 |
| DO48733 | https://dcc.icgc.org/donors/DO48733 |
| DO23527 | https://dcc.icgc.org/donors/DO23527 |
| DO23549 | https://dcc.icgc.org/donors/DO23549 |
| DO45179 | https://dcc.icgc.org/donors/DO45179 |
| DO23550 | https://dcc.icgc.org/donors/DO23550 |
| DO45221 | https://dcc.icgc.org/donors/DO45221 |
| DO50789 | https://dcc.icgc.org/donors/DO50789 |
| DO45129 | https://dcc.icgc.org/donors/DO45129 |
| DO50829 | https://dcc.icgc.org/donors/DO50829 |
| DO48743 | https://dcc.icgc.org/donors/DO48743 |
| DO50820 | https://dcc.icgc.org/donors/DO50820 |
| DO50808 | https://dcc.icgc.org/donors/DO50808 |
| DO23535 | https://dcc.icgc.org/donors/DO23535 |
| DO45209 | https://dcc.icgc.org/donors/DO45209 |
| DO50783 | https://dcc.icgc.org/donors/DO50783 |
| DO45093 | https://dcc.icgc.org/donors/DO45093 |
| DO50850 | https://dcc.icgc.org/donors/DO50850 |
| DO50800 | https://dcc.icgc.org/donors/DO50800 |
| DO45147 | https://dcc.icgc.org/donors/DO45147 |
| DO45243 | https://dcc.icgc.org/donors/DO45243 |
| DO48727 | https://dcc.icgc.org/donors/DO48727 |
| DO23546 | https://dcc.icgc.org/donors/DO23546 |
| DO23538 | https://dcc.icgc.org/donors/DO23538 |
| DO45261 | https://dcc.icgc.org/donors/DO45261 |
| DO45165 | https://dcc.icgc.org/donors/DO45165 |
| DO45101 | https://dcc.icgc.org/donors/DO45101 |
| DO45241 | https://dcc.icgc.org/donors/DO45241 |
| DO48730 | https://dcc.icgc.org/donors/DO48730 |
| DO23510 | https://dcc.icgc.org/donors/DO23510 |
| DO23524 | https://dcc.icgc.org/donors/DO23524 |
| DO45149 | https://dcc.icgc.org/donors/DO45149 |
| DO45217 | https://dcc.icgc.org/donors/DO45217 |
| DO48759 | https://dcc.icgc.org/donors/DO48759 |
| DO23515 | https://dcc.icgc.org/donors/DO23515 |
| DO48761 | https://dcc.icgc.org/donors/DO48761 |
| DO48715 | https://dcc.icgc.org/donors/DO48715 |
| DO50787 | https://dcc.icgc.org/donors/DO50787 |
| DO45177 | https://dcc.icgc.org/donors/DO45177 |
| DO45231 | https://dcc.icgc.org/donors/DO45231 |
| DO23511 | https://dcc.icgc.org/donors/DO23511 |
| DO50774 | https://dcc.icgc.org/donors/DO50774 |
| DO45171 | https://dcc.icgc.org/donors/DO45171 |
| DO45183 | https://dcc.icgc.org/donors/DO45183 |
| DO45283 | https://dcc.icgc.org/donors/DO45283 |
| DO23552 | https://dcc.icgc.org/donors/DO23552 |
| DO45235 | https://dcc.icgc.org/donors/DO45235 |
| DO50791 | https://dcc.icgc.org/donors/DO50791 |
| DO48704 | https://dcc.icgc.org/donors/DO48704 |
| DO50840 | https://dcc.icgc.org/donors/DO50840 |
| DO48697 | https://dcc.icgc.org/donors/DO48697 |
| DO45095 | https://dcc.icgc.org/donors/DO45095 |
| DO45275 | https://dcc.icgc.org/donors/DO45275 |
| DO45255 | https://dcc.icgc.org/donors/DO45255 |
| DO45131 | https://dcc.icgc.org/donors/DO45131 |
| DO45219 | https://dcc.icgc.org/donors/DO45219 |
| DO45099 | https://dcc.icgc.org/donors/DO45099 |
| DO45263 | https://dcc.icgc.org/donors/DO45263 |
| DO45257 | https://dcc.icgc.org/donors/DO45257 |
| DO23532 | https://dcc.icgc.org/donors/DO23532 |
| DO45233 | https://dcc.icgc.org/donors/DO45233 |
| DO50785 | https://dcc.icgc.org/donors/DO50785 |
| DO23526 | https://dcc.icgc.org/donors/DO23526 |
| DO45211 | https://dcc.icgc.org/donors/DO45211 |
| DO50819 | https://dcc.icgc.org/donors/DO50819 |
| DO45125 | https://dcc.icgc.org/donors/DO45125 |
| DO45143 | https://dcc.icgc.org/donors/DO45143 |
| DO50839 | https://dcc.icgc.org/donors/DO50839 |
| DO45215 | https://dcc.icgc.org/donors/DO45215 |
| DO50855 | https://dcc.icgc.org/donors/DO50855 |
| DO23529 | https://dcc.icgc.org/donors/DO23529 |
| DO45189 | https://dcc.icgc.org/donors/DO45189 |
| DO45253 | https://dcc.icgc.org/donors/DO45253 |
| DO50809 | https://dcc.icgc.org/donors/DO50809 |
| DO48728 | https://dcc.icgc.org/donors/DO48728 |
| DO45245 | https://dcc.icgc.org/donors/DO45245 |
| DO45121 | https://dcc.icgc.org/donors/DO45121 |
| DO45109 | https://dcc.icgc.org/donors/DO45109 |
| DO45223 | https://dcc.icgc.org/donors/DO45223 |
| DO50778 | https://dcc.icgc.org/donors/DO50778 |
| DO50845 | https://dcc.icgc.org/donors/DO50845 |
| DO48712 | https://dcc.icgc.org/donors/DO48712 |
| DO48747 | https://dcc.icgc.org/donors/DO48747 |
| DO45103 | https://dcc.icgc.org/donors/DO45103 |
| DO45191 | https://dcc.icgc.org/donors/DO45191 |
| DO45137 | https://dcc.icgc.org/donors/DO45137 |
| DO45113 | https://dcc.icgc.org/donors/DO45113 |
| DO45135 | https://dcc.icgc.org/donors/DO45135 |
| DO45247 | https://dcc.icgc.org/donors/DO45247 |
| DO48720 | https://dcc.icgc.org/donors/DO48720 |
| DO45239 | https://dcc.icgc.org/donors/DO45239 |
| DO48700 | https://dcc.icgc.org/donors/DO48700 |
| DO45157 | https://dcc.icgc.org/donors/DO45157 |
| DO48725 | https://dcc.icgc.org/donors/DO48725 |
| DO48736 | https://dcc.icgc.org/donors/DO48736 |
| DO48693 | https://dcc.icgc.org/donors/DO48693 |
| DO23528 | https://dcc.icgc.org/donors/DO23528 |
| DO45305 | https://dcc.icgc.org/donors/DO45305 |
| DO45307 | https://dcc.icgc.org/donors/DO45307 |
| DO45289 | https://dcc.icgc.org/donors/DO45289 |
| DO23547 | https://dcc.icgc.org/donors/DO23547 |
| DO45297 | https://dcc.icgc.org/donors/DO45297 |
| DO45279 | https://dcc.icgc.org/donors/DO45279 |
| DO45251 | https://dcc.icgc.org/donors/DO45251 |
| DO45187 | https://dcc.icgc.org/donors/DO45187 |
| DO48717 | https://dcc.icgc.org/donors/DO48717 |
| DO45303 | https://dcc.icgc.org/donors/DO45303 |
| DO45167 | https://dcc.icgc.org/donors/DO45167 |
| DO45195 | https://dcc.icgc.org/donors/DO45195 |
| DO23530 | https://dcc.icgc.org/donors/DO23530 |
| DO45285 | https://dcc.icgc.org/donors/DO45285 |
| DO45249 | https://dcc.icgc.org/donors/DO45249 |
| DO23523 | https://dcc.icgc.org/donors/DO23523 |
| DO23531 | https://dcc.icgc.org/donors/DO23531 |
| DO227801 | https://dcc.icgc.org/donors/DO227801 |
| DO227643 | https://dcc.icgc.org/donors/DO227643 |
