# Supplementary material for: Prognostic prediction and immune infiltration analysis based on ferroptosis and EMT state in hepatocellular carcinoma
Source: Front Immunol. 2022 Dec 15;13:1076045. doi: 10.3389/fimmu.2022.1076045 (PMC9797854; doi:10.3389/fimmu.2022.1076045)
Supplement: Supplementary file 6 [file Table_5.docx]

**Supplementary table 5** The model coefficients of FEPM trained from this study.

| Genes | Coefficients |
| --- | --- |
| PPARGC1A | -0.06833 |
| MMP1 | 0.045755 |
| STMN1 | 0.0279 |
| EZH2 | 0.108808 |
| STC2 | 0.060998 |
| KRT17 | 0.063728 |
| SPP1 | 0.046481 |
| SLC7A11 | 0.057713 |
| BSG | 0.040005 |
| MYCN | 0.165103 |
| SQSTM1 | 0.070954 |
| SRXN1 | 0.478141 |
| HOXD9 | 0.170427 |
